# REST reduction is essential for hypoxia-induced neuroendocrine differentiation of prostate cancer cells by activating autophagy signaling

**DOI:** 10.18632/oncotarget.8433

**Published:** 2016-03-28

**Authors:** Tzu-Ping Lin, Yi-Ting Chang, Sung-Yuan Lee, Mel Campbell, Tien-Chiao Wang, Shu-Huei Shen, Hsiao-Jen Chung, Yen-Hwa Chang, Allen W. Chiu, Chin-Chen Pan, Chi-Hung Lin, Cheng-Ying Chu, Hsing-Jien Kung, Chia-Yang Cheng, Pei-Ching Chang

**Affiliations:** ^1^ Institute of Clinical Medicine, National Yang Ming University, Taipei, Taiwan, R.O.C; ^2^ Department of Urology, School of Medicine, and Shu-Tien Urological Research Center, National Yang-Ming University, Taipei, Taiwan, R.O.C; ^3^ Department of Urology, Taipei Veterans General Hospital, Taipei, Taiwan, R.O.C; ^4^ UC Davis Cancer Center, University of California, Davis, CA, USA; ^5^ Department of Radiology, Taipei Veterans General Hospital, National Yang-Ming University, Taipei, Taiwan, R.O.C; ^6^ Department of Pathology, Taipei Veterans General Hospital, National Yang-Ming University, Taipei, Taiwan, R.O.C; ^7^ Department of Biochemistry and Molecular Medicine, University of California, Davis, CA, USA; ^8^ Institute for Translational Medicine, College of Medical Science and Technology, Taipei Medical University, Taipei City, Taiwan, R.O.C; ^9^ Division of Molecular and Genomic Medicine, National Health Research Institutes, Zhunan, Miaoli County, Taiwan, R.O.C; ^10^ Graduate Institute of Biomedical Electronics and Bioinformatics, National Taiwan University, Taipei, Taiwan, R.O.C; ^11^ Center for Infectious Disease and Cancer Research, Kaohsiung Medical University, Kaohsiung, Taiwan, R.O.C; ^12^ Institute of Microbiology and Immunology, National Yang-Ming University, Taipei, Taiwan, R.O.C

**Keywords:** REST, hypoxia, neuroendocrine differentiation, prostate cancer, autophagy

## Abstract

Prostate cancer (PCa) with neuroendocrine differentiation (NED) is tightly associated with hormone refractory PCa (HRPC), an aggressive form of cancer that is nearly impossible to treat. Determining the mechanism of the development of NED may yield novel therapeutic strategies for HRPC. Here, we first demonstrate that repressor element-1 silencing transcription factor (REST), a transcriptional repressor of neuronal genes that has been implicated in androgen-deprivation and IL-6 induced NED, is essential for hypoxia-induced NED of PCa cells. Bioinformatics analysis of transcriptome profiles of REST knockdown during hypoxia treatment demonstrated that REST is a master regulator of hypoxia-induced genes. Gene set enrichment analysis (GSEA) of hypoxia and REST knockdown co-upregulated genes revealed their correlation with HRPC. Consistently, gene ontology (GO) analysis showed that REST reduction potential associated with hypoxia-induced tumorigenesis, NE development, and AMPK pathway activation. Emerging reports have revealed that AMPK activation is a potential mechanism for hypoxia-induced autophagy. In line with this, we demonstrate that REST knockdown alone is capable of activating AMPK and autophagy activation is essential for hypoxia-induced NED of PCa cells. Here, making using of *in vitro* cell-based assay for NED, we reveal a new role for the transcriptional repressor REST in hypoxia-induced NED and characterized a sequential molecular mechanism downstream of REST resulting in AMPK phosphorylation and autophagy activation, which may be a common signaling pathway leading to NED of PCa.

## INTRODUCTION

Prostate cancer (PCa) is a leading cause of cancer morbidity and mortality in men. Its incidence is exponentially correlated with aging. The mainstay of treatment for PCa is androgen deprivation therapy (ADT) via surgical or chemical castration strategies, which unfortunately is not curative, as hormone-refractory (or castration-resistant) tumors eventually developed within few years after successful treatment by ADT [1]. Hormone refractory PCa (HRPC) is invariably lethal regardless of aggressive treatment [2]. Following the global population aging, the incidence rate of HRPC is rapidly increasing worldwide. Understanding of the mechanisms leading the development of HRPC will largely improve diagnostic, prognostic as well as therapeutic purposes. One promising but understudied feature of HRPC is its association with a neuroendocrine (NE) phenotype. NE differentiation (NED) is a process observed during the progression of PCa into castration-resistant state [3]. Androgen-independent NE-like cells are highly chemoresistant and secrete multiple cytokines to promote proliferation of neighboring non-NE cancer cells [4]. Identifying novel intervention strategies for the prevention or treatment of NE PCa is of great importance to improve the prognosis of HRPC. Though significant effort has been devoted to study the emergence of NE-like cells, the underlying mechanisms responsible for NED have yet to be fully identified. Multiple signaling pathways have been shown to induce NED in PCa cells, including androgen deprivation [5, 6] interleukin-6 (IL-6) treatment [7] and the recently identified contribution of hypoxia [8, 9].

Hypoxia is a characteristic feature of advanced solid tumors and strongly associates with malignant tumor progression and resistance to chemotherapy [10]. It also important for PCa development as evidence has shown a low oxygen level in PCa tumors [11] and a correlation of hypoxia with clinical stage [12], malignant progression [13], and radioresistance [14]. In addition to primary PCa, the rapid decrease of blood flow in prostate [15, 16] and in androgen-sensitive Shionogi tumor [17] under androgen deprivation has strengthened the potential link of hypoxia with ADT treatment. Emerging studies have suggested that hypoxia facilitates the malignancy of PCa cells by increasing androgen-independence [18, 19], the typical phenotype for HRPC. Interestingly, recent reports have also shown that hypoxia can induce NED through down-regulation of Notch signaling [8], a signaling pathway that is also required for normal prostate development [20, 21]. However, there is no direct evidence showing the capability of preventing hypoxia-induced NED by reactivation of Notch-signaling. Together, this evidence indicates an association of hypoxia with the malignant transformation of PCa into androgen-independent NE tumor. However, little is known about the mechanisms that underlie hypoxia-induced NED.

Two recent reports including ours have shown that autophagy activation in PCa LNCaP cells is essential for the NED of PCa cells [22, 23]. Autophagy is a regulated degradation pathway that cells use to recycle long-lived proteins and organelles to survive metabolic stress, such as starvation [24–26] and hypoxia [27, 28]. A growing body of research supports the notion that autophagy is hijacked by cancer cells, and in turn, its activation serves as a survival mechanism to protect malignant cells from unfavorable environments. In PCa, ADT treatment leads to autophagy activation, which in turn overcomes the metabolic stress of androgen withdraw and facilitates cell survival [29, 30] and cancer progression into HRPC [31]. Autophagy activation under hypoxia also contributes to radio- and chemo-resistance of tumor cells [32, 33]. However, in PCa, all the knowledge of hypoxia-induced autophagy is limited to its function as a “survival mechanism” in androgen-dependent PCa cells. The potential roles and underlying mechanisms by which autophagy mediates hypoxia-induced NED of PCa cells has never been studied. The regulatory mechanism of autophagy can be classified into two broad categories: mTOR-dependent and -independent pathways, based on the dependency of mTOR, the key negative regulator of the autophagy pathway. AMPK signaling is a minor mechanism upstream of mTOR that links the intracellular energy status to autophagy activation during energy stress [34]. Interestingly, autophagy activation by hypoxia in tumor cells [35], including PCa, is largely dependent on the AMPK pathway [30, 36]. Our recent report also showed that AMPK activation is essential for autophagy activation during IL-6-induced NED of PCa cells [30].

A recent study showed that repressor element-1 silencing transcription factor (REST), also known as neuron restrictive silencing factor (NRSF), is a mediator of androgen receptor (AR) and inhibits androgen-deprivation induced NED [37]. Our recent report also showed that IL-6, a pleiotropic cytokine that is significantly upregulated in PCa patients' serum undergoing ADT treatment, induces NED through down-regulation of REST [23]. REST was originally identified as a critical transcription repressor that plays an important role in silencing neuronal gene expression in neural progenitor and non-neuronal cells [38–40]. Whilst emerging evidence shows that REST is involved in neurobiology, it also functions as a regulator for oncogenic transformation [41–43]. The level of REST protein is tightly regulated by β-TrCP ubiquitin ligase through a ubiquitin-proteasomal degradation mechanism [37, 42, 44]. Although post-translational control mediated by ubiquitylation has been implicated as important in regulating protein levels of REST, the transcription/translation regulation of REST has also been identified in the Wnt signaling pathway through the β-catenin-TCF complex [45]. A more recent study also showed that REST can be down-regulated by hypoxia-induced microRNAs (miR) in advanced PCa [9]. In addition to neural and NED genes, our recent report showed that REST also targets autophagy-related genes. Most interestingly, we found that REST knockdown alone enhances autophagy activation [23]. Here, we sought to determine whether REST protein down-regulation is also involved in hypoxia-induced NED and autophagy activation.

To address our hypothesis, the effects of down-regulation of REST under hypoxic stimuli and the ability of REST overexpression to inhibit hypoxia-induced NED were determined in androgen-sensitive LNCaP cells. Consistent with our hypothesis, REST protein was decreased under hypoxia in a time-dependent manner and hypoxia-induced neurite extension was significantly inhibited by REST overexpression. Bioinformatic analysis of the genes upregulated by hypoxia and REST knockdown showed that these genes were significantly enriched in the AMPK signaling pathway. Consistent with the bioinformatic observations from RNA-seq data, AMPK activation and mTOR inhibition was demonstrated in REST knockdown as well as in hypoxia treatment. Most importantly, our study demonstrates for the first time that autophagy plays a crucial role in hypoxia-induced NED.

## RESULTS

### REST is crucial for hypoxia-induced NED

**Figure 1:**
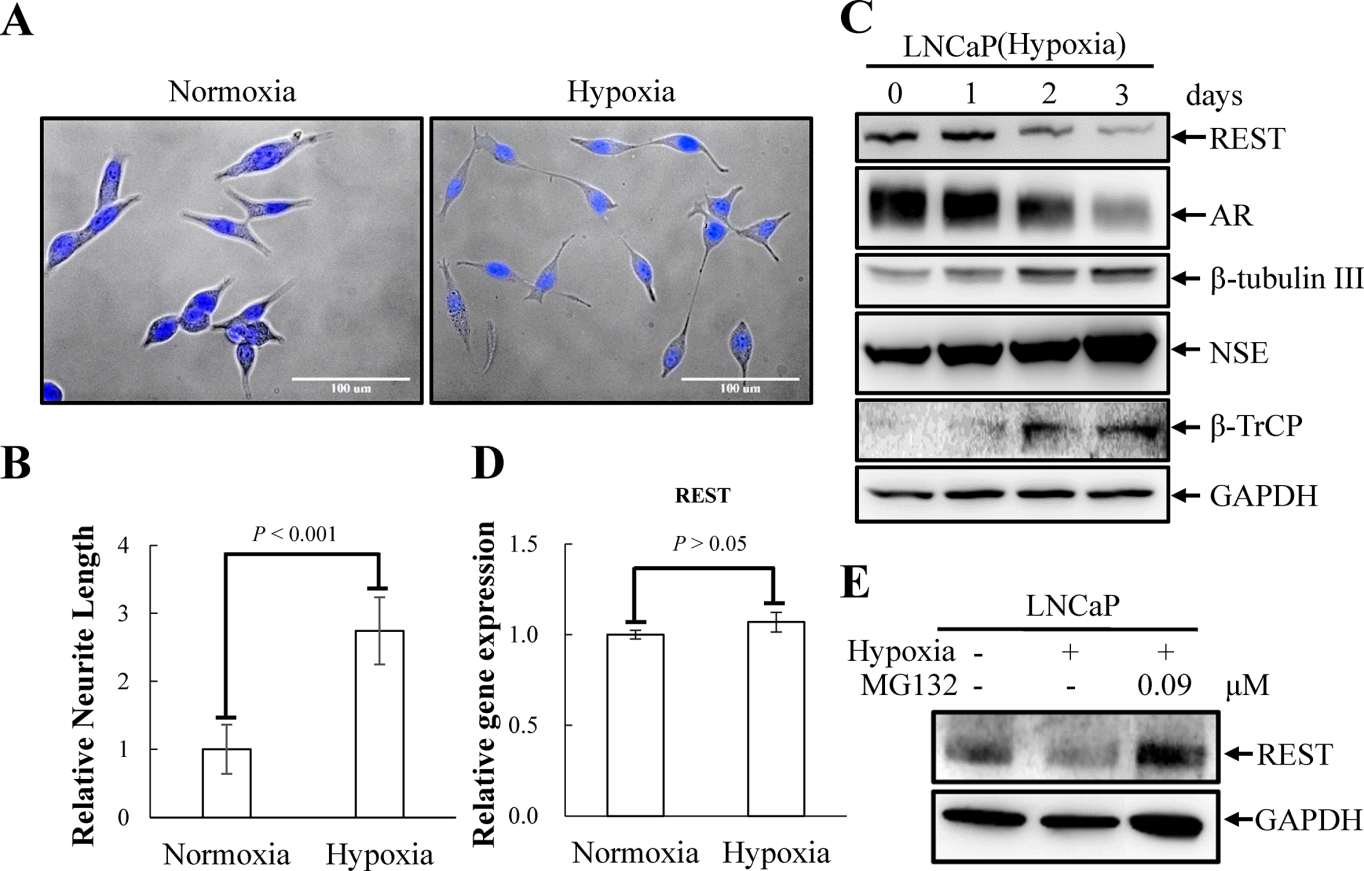
Hypoxia induces NED of LNCaP cells concomitant with down-regulation REST protein levels but not REST mRNA (**A**) LNCaP cells were treated with hypoxia (2% O_2_) for 3 days. Representative photos of control and hypoxia-treated cells were stained with Hoechst. (**B**) The induced neurite length was assessed using brightfield microscopy images (40× magnification) and quantified by the average from 10 microscopic fields; bars, SD. (**C**) Total cell lysates (TCLs) were prepared from LNCaP cells treated as described in (A) for 1, 2 and 3 days and then immunoblotted to detect REST, AR, β-tubulin III, NSE, and β-TrCP. GAPDH was used as the loading control. (**D**) RT-qPCR analysis of total RNA from LNCaP cells treated as described in (A). The relative mRNA level of REST was normalized with B2M. Values from 3 independent experiments are reported as mean ± SD. (**E**) LNCaP cells were treated with hypoxia for 3 days in the presence or absence of 0.09 μM MG-132. The expression of REST was detected by immunoblotting using anti-REST antibody. GAPDH was used as the loading control.

**Figure 2:**
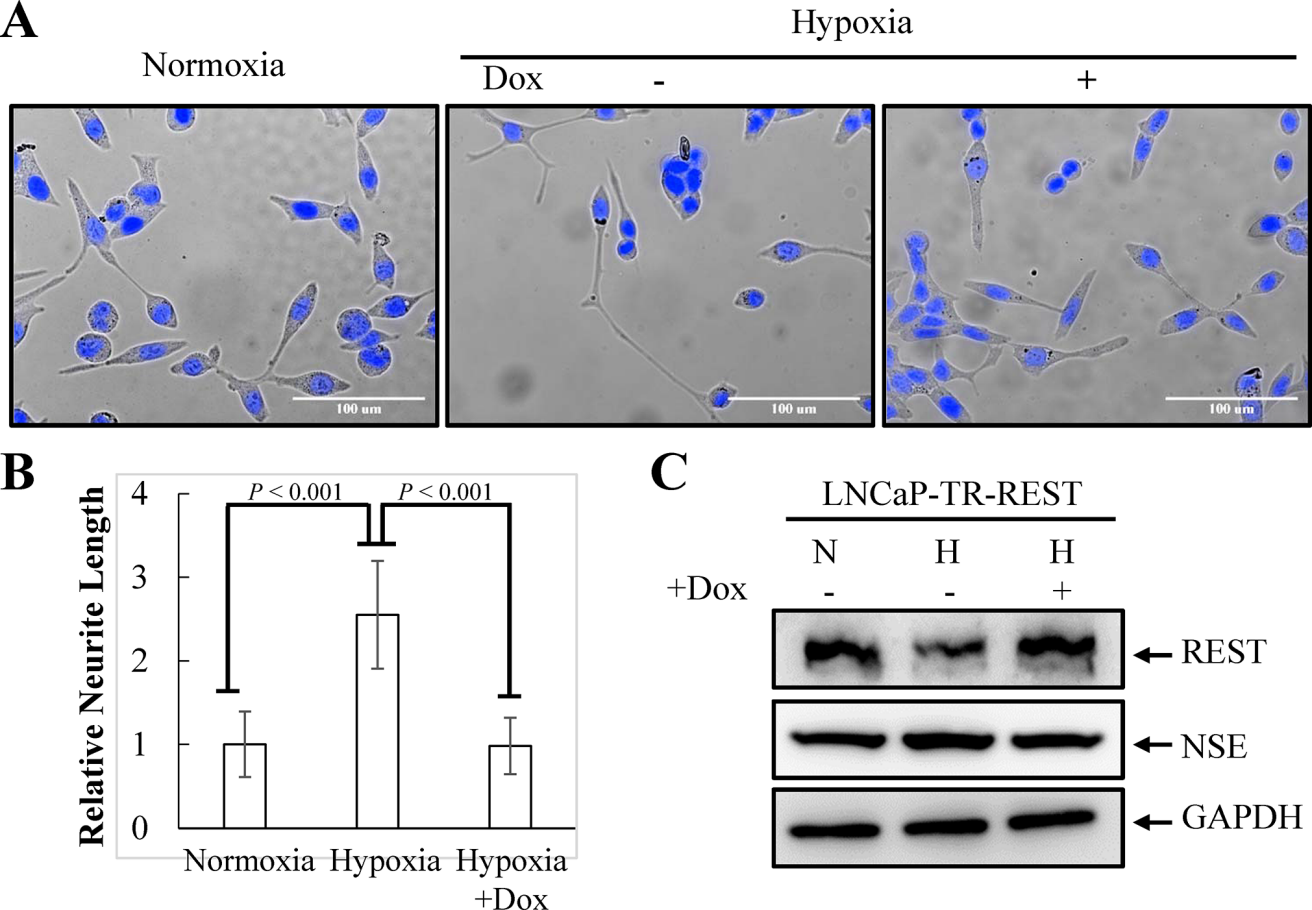
Inhibition of neurite elongation by REST overexpression (**A**) LNCaP-TR-REST cells were treated with 0.001 μg/ml Dox under normoxia or hypoxia (2% O_2_) conditions for 4 days. LNCaP-TR-REST cells without Dox treatment under normoxia was used as control. Representative photos of control and hypoxia-treated cells with or without REST overexpression were stained with Hoechst. (**B**) The neurite length was assessed using brightfield microscopy images (40× magnification) and quantified by the average from 10 microscopic fields; bars, SD. (**C**) The expression of REST and NSE under hypoxia treatment as described in (A) was confirmed using anti-REST and anti-NSE specific antibodies. GAPDH was used as the loading control.

Hypoxia has recently been shown to induce NED and concomitantly reduce REST in PCa cells [9]. Since our recent report showed that REST is essential for IL-6-induced NED [23], we sought to determine whether REST may function as a crucial factor for NED induced by hypoxia as well. To verify this hypothesis, hypoxia-induced NED was first confirmed in LNCaP cells. These cells are an androgen-dependent human prostate adenocarcinoma cell line that can rapidly acquire the NE phenotype with neuron-like extensions and elevated expression of neuronal markers, such as β-tubulin III and neuron specific enolase (NSE). Consistent with other reports [8, 9], neurite extension was enhanced in LNCaP cells after exposure to hypoxia (2% O_2_) for 3 days (Figure 1A and 1B). In addition, an increase in β-tubulin III and neuron specific enolase (NSE) and a decrease in AR protein expression was observed 2 days after reduction of O_2_ tension (Figure 1C). Secondly, the protein level of REST was analyzed by immunoblotting and a reduction of REST was identified at day 2 after hypoxia exposure (Figure 1C). The temporal opposition between NED markers and REST protein levels support the potential role of REST in mediating hypoxia-induced NED. Recently, Liang *et al.* showed that the down-regulation of REST protein during hypoxic conditions is mediated by transcriptional regulation of microRNAs [9]. Therefore, the mRNA level of REST was examined. However, we did not detect significant change of REST mRNA expression (Figure 1D). Since the expression of REST is well-known to be regulated via ubiquitin ligase β-TrCP in a proteasome-dependent manner [44], our data suggest that the hypoxia-induced down-regulation of REST is at the protein level. Consistently, immunoblotting showed an increase in β-TrCP (Figure 1C). Moreover, the inhibition of protein degradation by proteasome inhibitor MG-132 reversed hypoxia-induced REST down-regulation (Figure 1E). Thirdly, the role of REST in mediating NED was studied by using a REST inducible knockdown cell line, LNCaP-TR-shREST, generated in our previous study [23]. Indeed, REST knockdown alone increased neurite extension and β-tubulin III protein expression and decreased AR of LNCaP cells in a time-dependent manner (Figure S1). Conversely, using a REST inducible cell line, LNCaP-TR-REST [23], hypoxia-induced NED of LNCaP cells was significantly inhibited by REST overexpression (Figure 2). Together, these data demonstrate that REST plays an important role in hypoxia-mediated NED of PCa cells.

### Identification of REST as a novel key factor of hypoxia response

**Figure 3:**
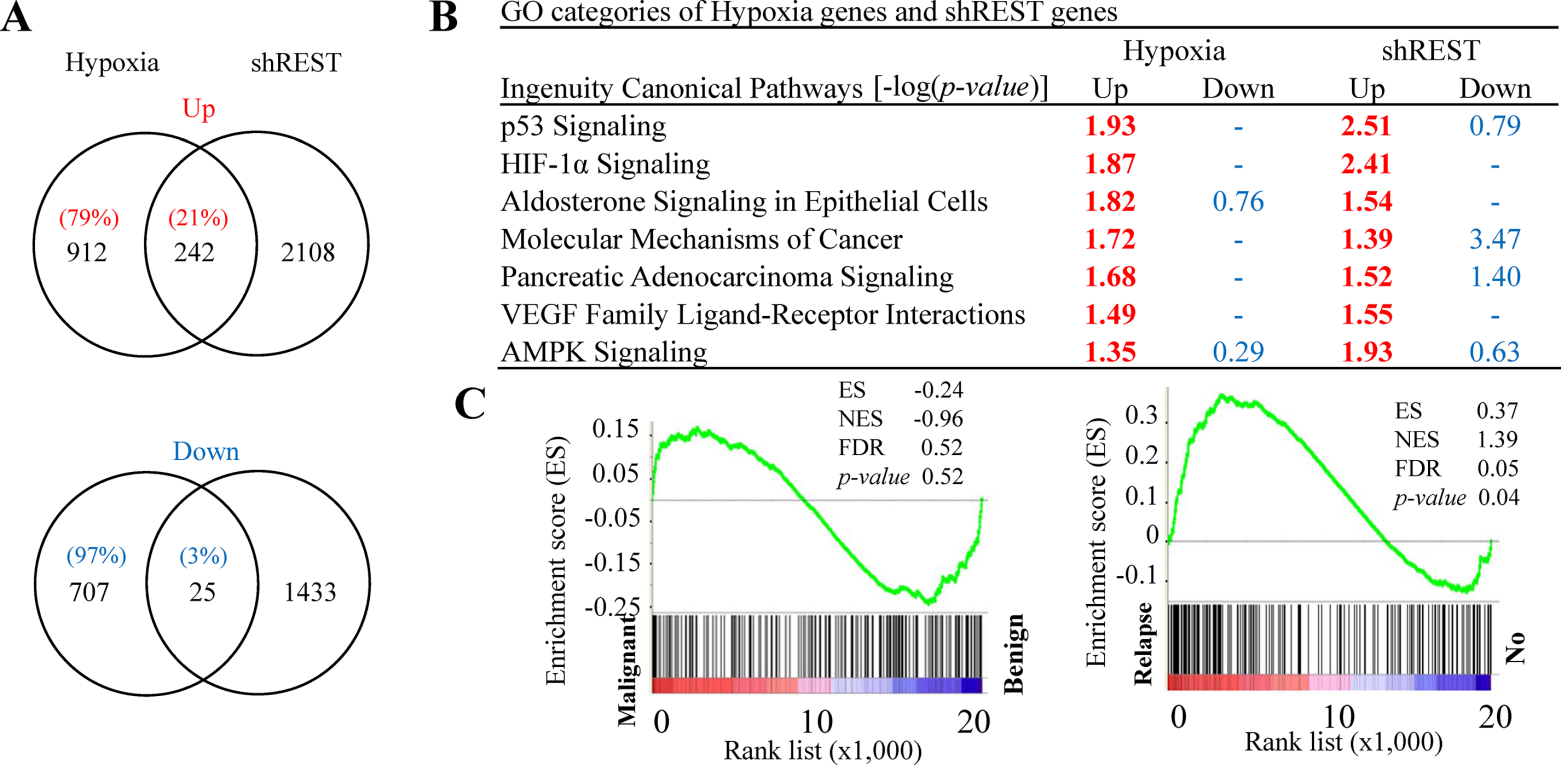
Comparison of RNA-seq data sets between REST knockdown and hypoxia-treated LNCaP cells (**A**) Venn diagrams depict the overlapped up- and down-regulated genes in REST knockdown and hypoxia-treated (2% O_2_) LNCaP cells. Total numbers of genes are listed inside the circles and the percentages of hypoxia-regulated genes are given. (**B**) Ingenuity canonical pathway (IPA) analysis of REST knockdown and hypoxia up- and down-regulated genes. –log (*p* value) > 1.3 was considered statistically significant and labeled in red. (**C**) The GSEA result showing the correlation of REST knockdown and hypoxia co-upregulated genes with PCa relapse (right panel) but not the malignance (left panel).

Given that REST is a transcriptional repressor that orchestrates epigenetic remodeling and suppresses the expression of genes involved in many aspects of physiological regulation, we hypothesized a possible role of down-regulation of REST in up-regulating gene expression in response to hypoxia. To examine this possibility, genome-wide transcriptome analysis was performed using RNA-seq. We analyzed gene expression changes induced by (a) hypoxia treatment for 3 days in comparison to normoxia control and (b) REST knockdown for 3 and 6 days compared with non-induced control. Transcript abundance was calculated as fragments per kilobase of transcript per million mapped reads (FPKM) using Cufflinks. Genes with FPKM > 1 were considered to be expressed and used for further analysis. FPKM with 1.5-fold change was considered as differentially expressed genes. Of the 1154 genes up-regulated by hypoxia (Table S1), 242 genes (~21%) were also up-regulated by REST knockdown for 3 or 6 days (Table S2). However, only 25 genes (~3%) among the 732 hypoxia down-regulated genes (Table S3) were simultaneously down-regulated during REST knockdown (Table S4). As shown in Figure 3A, these results indicate that down-regulation of REST may mediate the transcriptional activation of a specific group of hypoxia-responsive genes after hypoxic stimulation. To identify the function of those genes, we carried out gene ontology (GO) analysis of genes up- and down-regulated in response to hypoxia and REST knockdown by using Ingenuity Pathway Analysis (IPA) software. GO pathway analysis showed that the 1154 up-regulated genes by hypoxic stimulation and 2350 up-regulated genes by REST knockdown were simultaneously significantly enriched in seven ingenuity canonical pathways (Figure 3B). Qi *et al.* have shown the critical role of HIF-1α signaling in neuroendocrine phenotype [46]. The identification of HIF-1α pathway supports the importance of REST in hypoxia signaling and neuroendocrine differentiation. In addition, numerous cancer-related signaling pathways including pancreatic adenocarcinoma, p53, and VEGF were also observed. This suggests the role of REST in mediating hypoxia-induced tumorigenesis in PCa. Aldosterone is a steroid hormone. The identification of aldosterone signaling of epithelial cells implicates the involvement of hypoxia and REST knockdown-co-regulated genes in regulating steroid hormone signaling. To identify common gene networks between the hypoxia and REST knockdown-co-regulated genes and PCa progression, gene set enrichment analysis (GSEA) was performed using microarray datasets from two separate GEO database: (1) GSE55945: benign versus PCa tissues; and (2) GSE33316: LuCaP35 xenografts before versus after castration. Consistent with the high correlation between REST down-regulation in hypoxia and NED of PCa cells, GSEA revealed that the expression of the 242 genes co-upregulated in hypoxia and REST knockdown was correlated with castration (GSE33316) (Figure 3C, left panel) but not the PCa formation (GSE55945) (Figure 3C, right panel).

### Activation of AMPK/mTOR pathway following knockdown of REST

**Figure 4:**
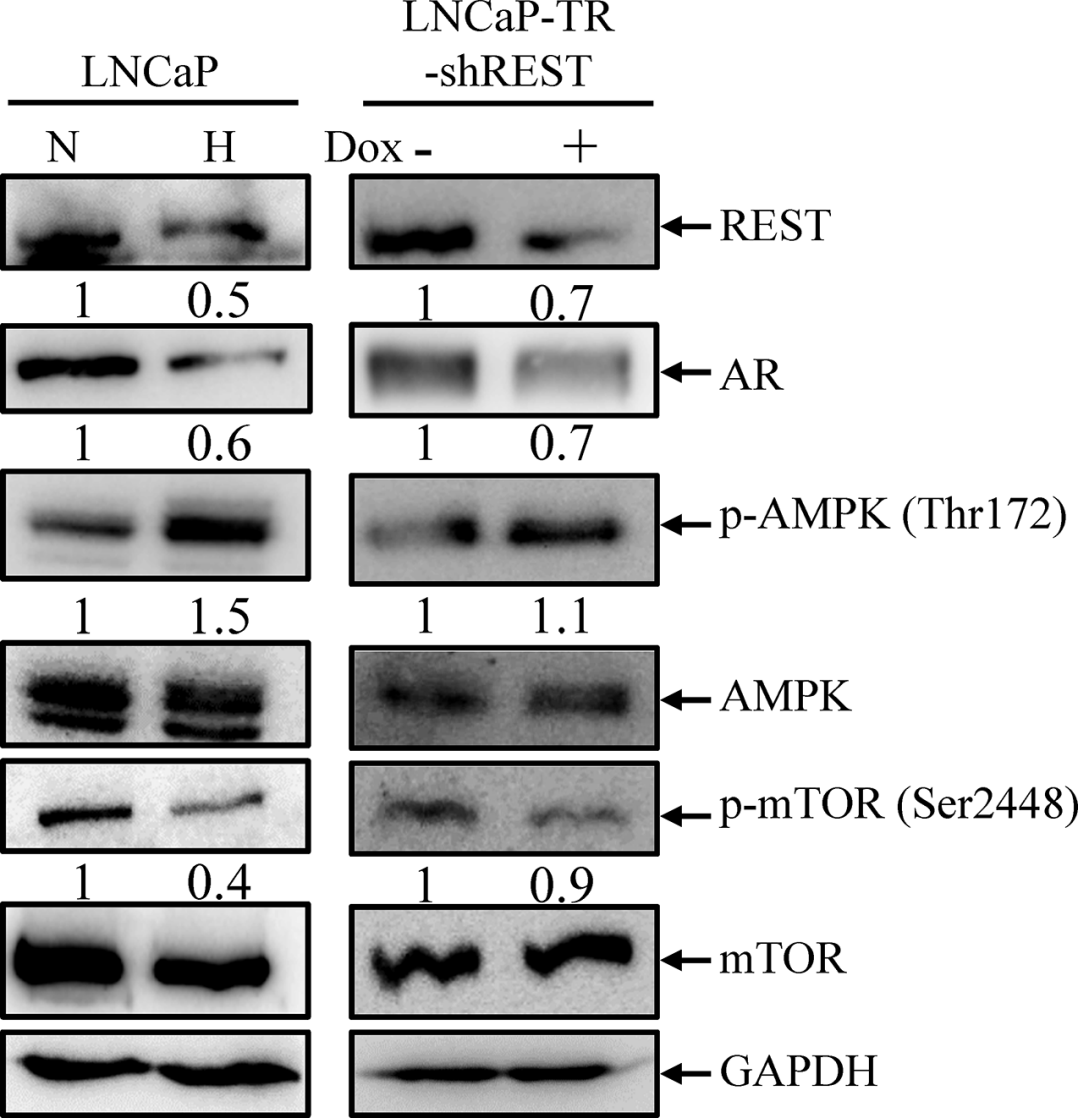
AMPK/mTOR pathway is activated by hypoxia treatment and REST knockdown (**A**) LNCaP cells were treated with normoxia (N) or hypoxia (H) (2% O_2_) for 3 days (left panel). LNCaP-TR-shREST cells were treated with 1 μg/ml Dox for 3 days to knockdown REST (right panel). Total cell lysates (TLCs) were analyzed by immunoblotting using anti-REST, anti-AR, anti-p-AMPK, and anti-p-mTOR antibodies. Total AMPK and mTOR are used as controls. GAPDH was used as loading control. (**B**) The expression of each protein in three independent experiments was quantified; bars, SD.

**Figure 5:**
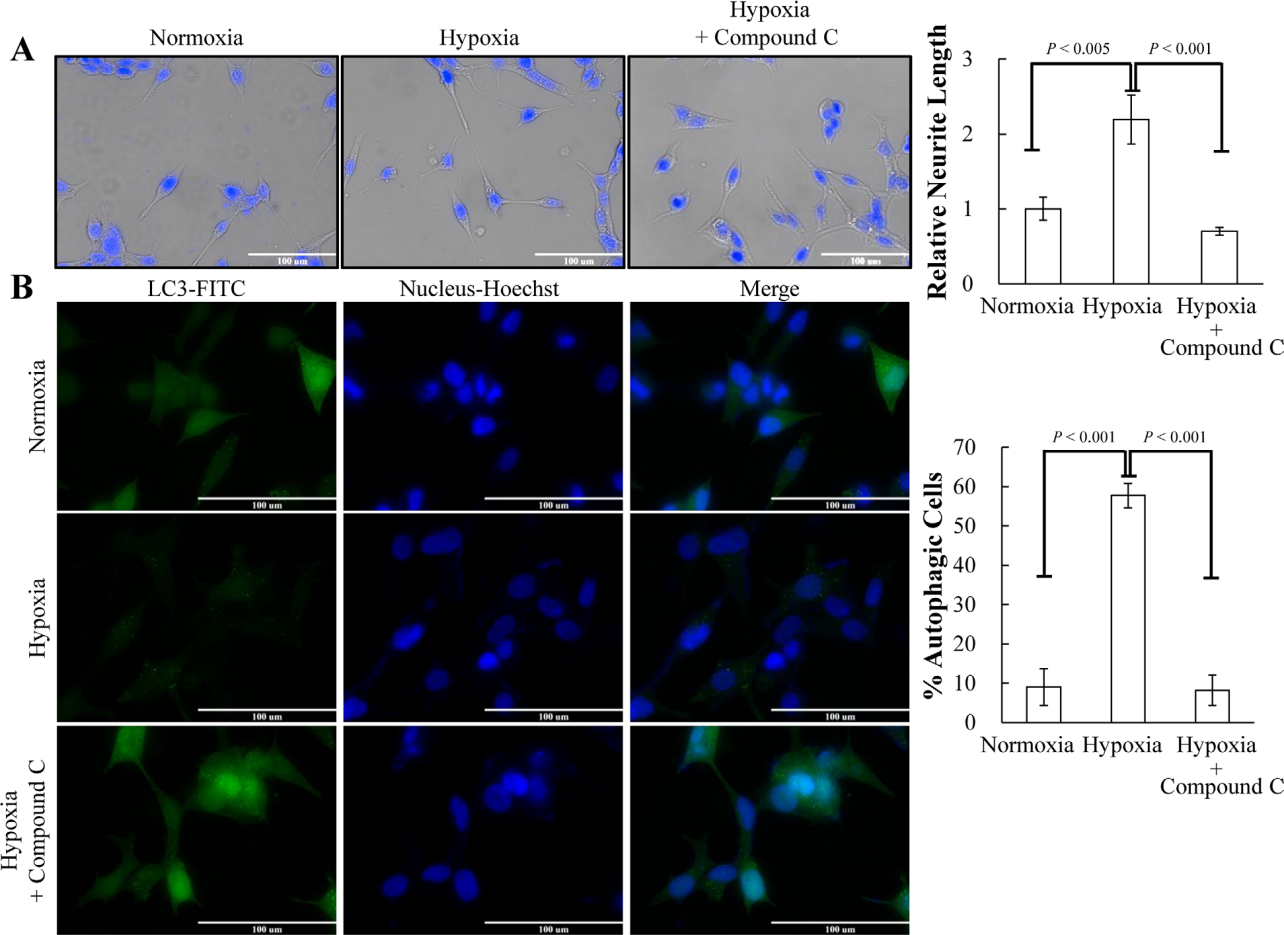
AMPK pathway is essential for hypoxia-induced NED and autophagy activation (**A**) LNCaP cells were treated with hypoxia (12% O_2_) for 3 days in the presence or absence of 2.5 μM compound C. Representative images of normoxia and hypoxia-treated cells with or without compound C were stained with Hoechst (left panel). The neurite length was assessed using brightfield microscopy images (40× magnification) and quantified by the average from 10 microscopic fields; bars, SD (right panel). (**B**) LNCaP-eGFP-LC3 cells were treated with hypoxia for 3 days in the presence or absence of 2.5 μM compound C. Following fixation, the cells were stained with Hoechst (Blue) and analyzed by fluorescence microscopy (FITC, 63× magnification) (left panel). The percentage of cells with punctate eGFP-LC3 expression was calculated using 10 microscopic fields and analyzed by MetaMorph software (right panel).

The most important finding in GO analysis was the concomitant activation of AMPK pathway by hypoxic stimulation and REST knockdown (Figure 3B). AMPK is the most well-known pathway mediating hypoxia-induced autophagy activation [30, 36, 47]. Our recent report also showed that AMPK is essential for IL-6 mediated autophagy activation [23]. To verify the AMPK activation by hypoxia and REST knockdown, LNCaP cells were subjected to hypoxia treatment for 3 days and LNCaP-TR-shREST were subjected to knockdown of REST in the presence of Dox for 3 days. Activity of AMPK and mTOR were monitored by their phosphorylation status. As shown in Figure 4, hypoxia treatment and partial REST knockdown both caused an increase of phospho-AMPK. Total AMPK remained unchanged in both conditions. To determine if the AMPK activation by REST knockdown mediates AMPK-induced mTOR inhibition in PCa cells, phospho-mTOR levels were also examined in these samples. Consistent with the phosphorylation pattern of AMPK, phospho-mTOR decreased in both hypoxia treatment and REST knockdown (Figure 4). Total mTOR was unchanged in both conditions. The expression level of each protein was examined in three independent experiments. Consistently, the levels of phospho-AMPK and phospho-mTOR under hypoxia condition with REST overexpression were comparable to normoxia (Figure S2). The function of AMPK in hypoxia-induced NED of LNCaP cells was further confirmed using AMPK inhibitor, compound C. Compound C significantly reduced the NED (Figure 5A) and autophagy (Figure 5B) induced by hypoxia. Moreover, inhibition of mTOR by rapamycin significantly increased NED of LNCaP cells (Figure S3A) However, rapamycin-induced autophagy activation did not alter the protein level of REST (Figure S3B). These results indicate that REST down-regulation may be one of the underlying mechanisms that contribute to hypoxia-induced AMPK activation and mTOR inhibition.

### Autophagy activation is essential for hypoxia-induced NED

**Figure 6:**
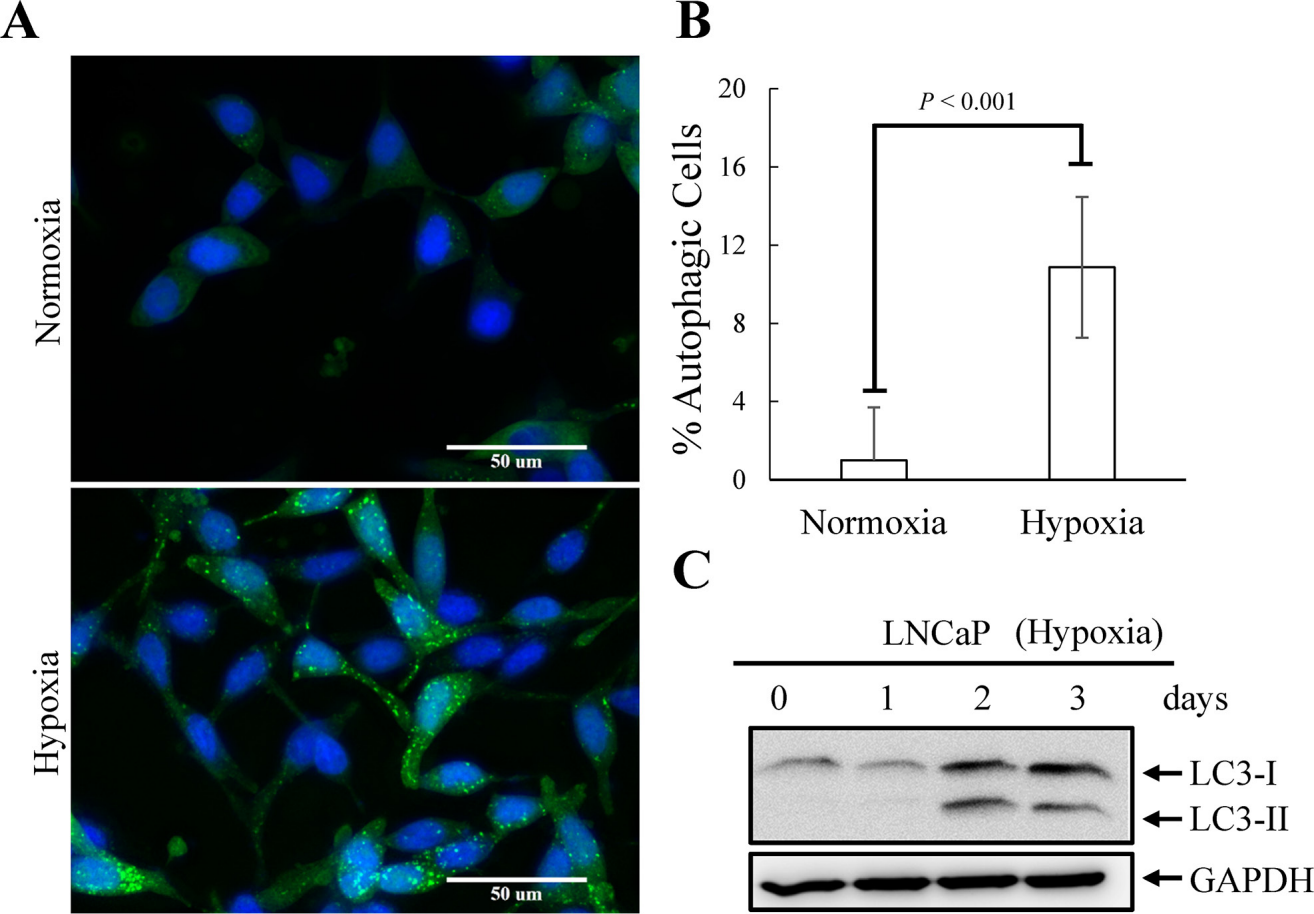
Hypoxia-induced autophagy activation of LNCaP cells (**A**) LNCaP-eGFP-LC3 cells were treated with hypoxia (2% O_2_) for 3 days. Normoxia was used as control. Following fixation, the cells were stained with Hoechst (Blue) and analyzed by fluorescence microscopy (FITC, 63× magnification). (**B**) The percentage of cells with punctate eGFP-LC3 expression was calculated using 10 microscopic fields and analyzed by MetaMorph software. (**C**) TCLs were prepared from LNCaP cells treated with hypoxia for 1, 2 and 3 days and then immunoblotted to detect LC3. GAPDH was used as the loading control.

**Figure 7:**
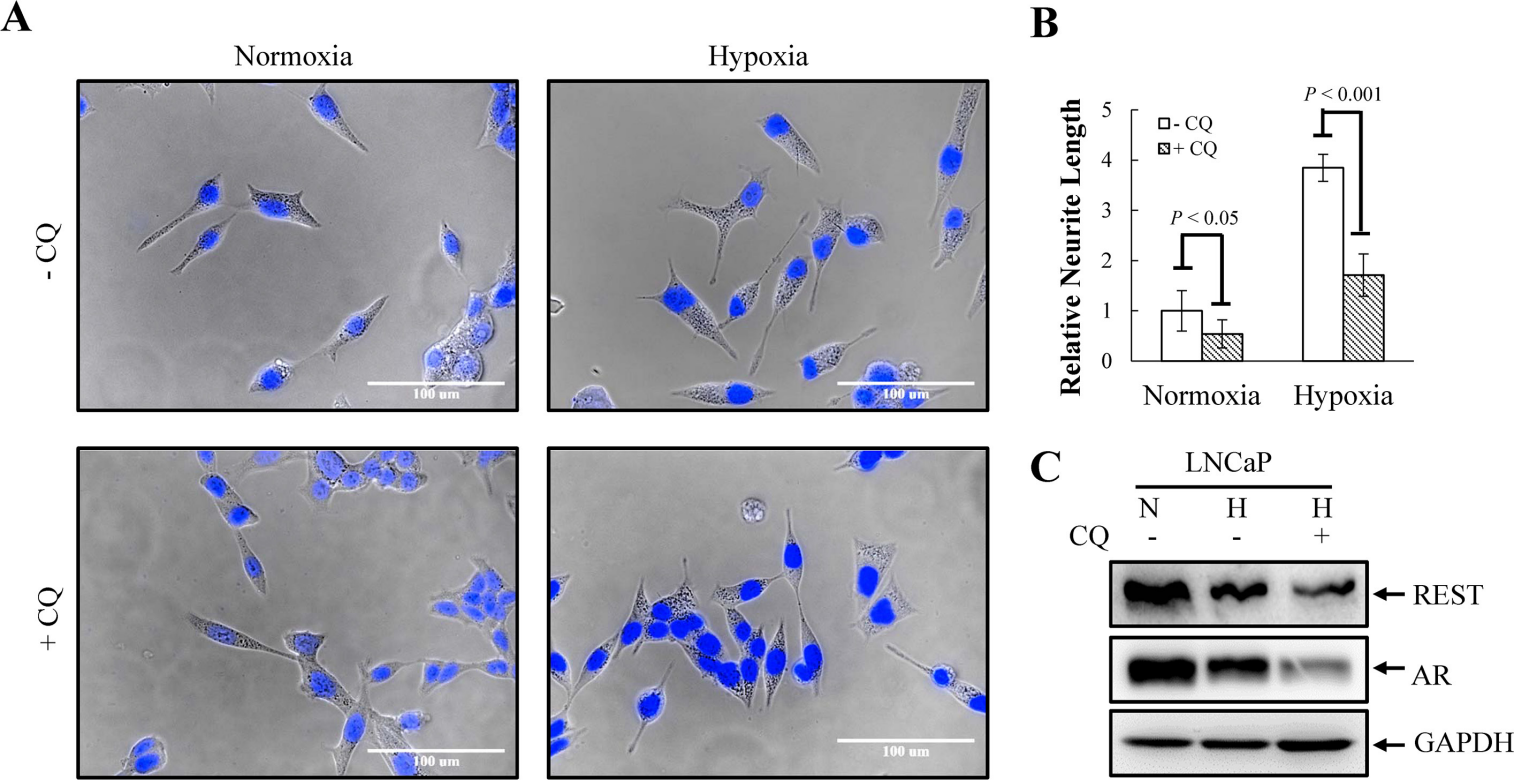
Chemical inhibition of autophagy flux by chloroquine (CQ) suppresses hypoxia-induced NED in LNCaP cells (**A**) LNCaP cells were treated with hypoxia (2% O_2_) in the absence or presence of 50 μM CQ for 3 days. Following nuclear counterstaining with Hoechst (Blue), neurite structures were assessed by microscopy images (40× magnification). (**B**) Neurite lengths were quantified using 10 fields; bars, SD. (**C**) TLCs prepared from LNCaP cells in normoxia or treated with hypoxia in the absence or presence of 50 μM CQ for 3 days were analyzed by immunoblotting using the indicated antibodies.

**Figure 8:**
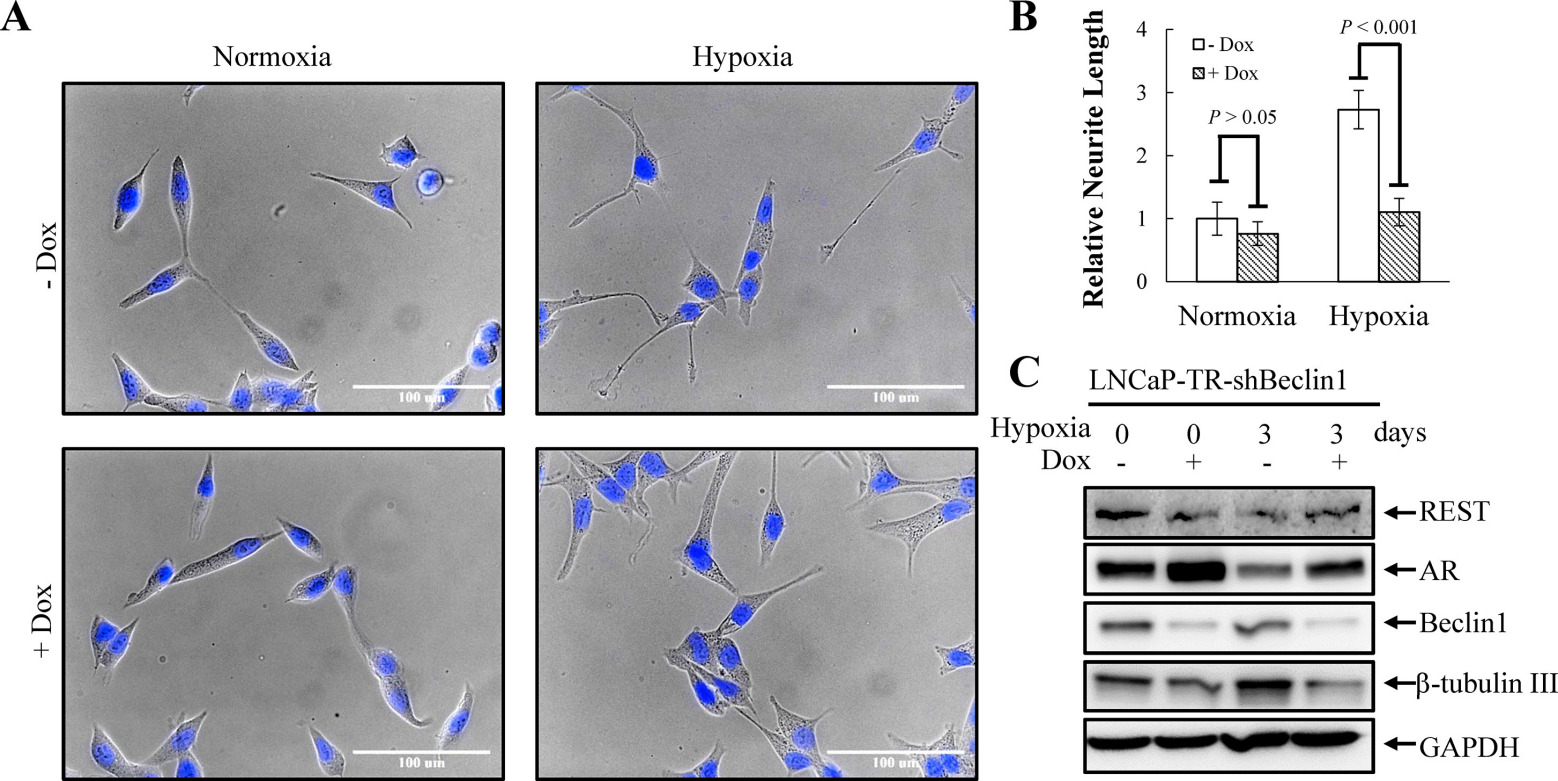
Knockdown of beclin 1 suppresses hypoxia-induced NED (**A**) LNCaP-TR-shBeclin1 cells were treated with 1 μg/ml Dox to induce beclin 1 knockdown for 4 days under normoxia and hypoxia (2% O_2_). Following nuclear counterstaining with Hoechst (Blue), the neurite structures were assessed by microscopy images (40×magnification). (**B**) The neurite length was quantified by 10 microscopic fields; bars, SD. (**C**) TLCs were prepared from LNCaP-TR-shBeclin1 cells treated as described in (A) and analyzed by immunoblotting using the indicated antibodies.

**Figure 9:**
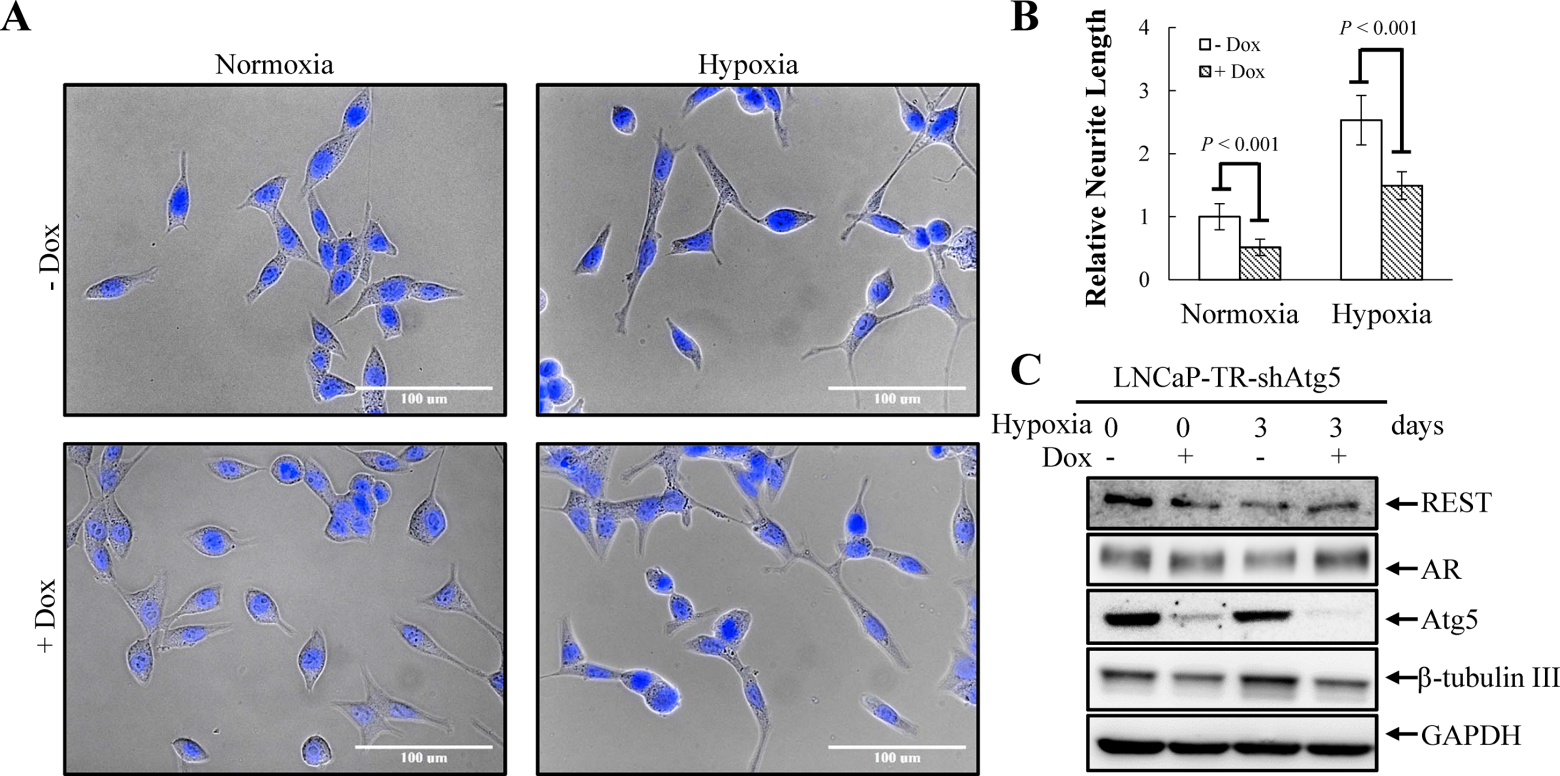
Knockdown of Atg5 suppresses hypoxia-induced NED (**A**) LNCaP-TR-shAtg5 cells were treated with 1 μg/ml Dox to induce Atg5 knockdown for 4 days under normoxia and hypoxia (2% O_2_). Following nuclear counterstaining with Hoechst (Blue), the neurite structures were assessed by microscopy images (40× magnification). (**B**) Neurite lengths were quantified by 10 microscopic fields; bars, SD. (**C**) TLCs were prepared from LNCaP-TR-shAtg5 cells treated as described in (A) and analyzed by immunoblotting using the indicated antibodies.

Tumor hypoxia is well recognized as an indicator of poor prognosis in PCa [48]. Increasing evidence has revealed that enhanced autophagy favors PCa cell survival under hypoxia [30]. However, the role of autophagy in hypoxia-induced NED of PCa has never been studied. To study this, the activation of autophagy by hypoxia was first examined using LNCaP-eGFP-LC3 cell line, a LNCaP derived cell line stably expressing eGFP-LC3 that we generated in our previous study [23]. Autophagic cells were counted as the number of cells with punctate expression eGFP-LC3. Consistent with previous reports, hypoxia increased the numbers of cells undergoing autophagy to 12% (Figure 6A and 6B). The induction of autophagy by hypoxia was confirmed by immunoblotting using the LNCaP cells. Hypoxia significantly increased the conversion of LC3-I to LC3-II (Figure 6C). To determine the dispensability of autophagy induction on hypoxia-induced NED, we inhibited autophagy using chloroquine (CQ). As shown in Figure 7A, CQ strongly inhibited hypoxia-induced NED of LNCaP cells. Quantitation of neurite length using MetaMorph showed there was a significant inhibition of neurite extension (Figure 7B). However, the reduction of REST and AR under hypoxia condition was not reversed by CQ (Figure 7C). Consistent with our hypothesis, this result showed that autophagy activation is downstream of REST. Since chemical inhibition of autophagy by CQ may have non-specific effects, we further confirmed the importance of the autophagic pathway in hypoxia-induced NED of PCa cells using small hairpin RNAs (shRNA)-silencing approach. The LNCaP-TR-shBeclin 1 and shAtg5 cell lines [23] that were able to induce knockdown of beclin 1 (Atg6) and Atg5 after Dox treatment for 4 days were used. In support of our hypothesis, beclin 1 and Atg 5 knockdown cells both display a significantly lower degree of NED than control cells after hypoxia treatment (Figures 8A and 9A). Quantification data showed that both beclin 1 and Atg5 knockdown had a significant inhibitory effect on NED of PCa cells (Figures 8B and 9B). Consistent with the cell morphology data, PCa cell β-tubulin III protein level decreased following inhibition of NED by beclin 1 and Atg5 knockdown (Figures 8C and 9C). Taken together, these data demonstrate that activation of autophagy participates in hypoxia-induced NED of PCa cells.

## DISCUSSION

PCa is exponentially related to age and, therefore, is a major cancer affecting old men. Due to the global trends in population aging, PCa incidence has increased dramatically in recent years. Though primary PCa can be successfully treated by ADT, hormone-refractory tumors eventually occur within few years. HRPC patients are no longer response to ADT treatment and are highly resistant to radio- and chemotherapy. Therefore, HRPC ultimately leads PCa patients entering the lethal stage. Again, the accompaniment with the current global aging issue, HRPC makes PCa a leading cause of cancer death worldwide for males. Therefore, identification of potential mechanisms leading to the development of HRPC has become an important research area. Multiple mechanisms have been proposed to explain the transition of androgen-dependent PCa to androgen-independent HRPC, and most of these focus on how PCa cells adapt to ADT by mutation in the androgen receptor. In addition to androgen-independence, NED is an emerging phenotype that was recently recognized as an underlying cause of HRPC. Identification of therapeutic strategies that prevent or delay NED during ADT treatment will likely increase the effectiveness of ADT treatment. However, due to the limited knowledge on the mechanisms leading to NED, no therapeutic strategies are currently available to prevent NED or eliminate NE-like cells.

Patients who receive longer-duration ADT have higher levels of NED [49, 50]. Supporting the observation among clinical specimens, androgen withdrawal induced NED of PCa cells both in *in vitro* cell culture [51, 52] and *in vivo* animal model [53]. In addition, increasing evidence shows that NED is also induced by IL-6, a cytokine that was significantly increased in the serum of patients undergoing ADT and clinically relevant to castration-resistant progression and metastasis of PCa [54–56]. Moreover, androgen withdraw and IL-6 showed a synergistic effect in induction of NED in PCa cells [23]. This result indicates that alteration of physiological conditions by ADT might create or even augment the severity of NED. Consistent with this concept, several recent reports showed that hypoxia, a common feature of PCa associated with malignant progression and resistance to therapy, also induced NED of PCa [8, 9]. These studies were based on the observation that HIF-1α, the master regulator of hypoxia response, is expressed in NE tumors [57]. Moreover, HIF-1α signaling showed a critical role in the neuroendocrine phenotype [46]. A reduction in blood flow in prostate following ADT treatment has been implicated in the induction of hypoxia [15, 16]. Although the phenomena of PCa hypoxia following ADT are conflicting, as some studies reported an increase in PCa oxygenation [58] and HIF-1α protein expression drop [59] following castration; this contradiction was considered a time-dependent heterogeneity of response [60]. Induction of hypoxia is seen in the early stage of ADT. The different effects observed along the timeline post-ADT may due to the latent revascularization of tumor. Altogether, pathways and mechanisms that promote NED by different inducers, including androgen withdraw, IL-6 and hypoxia, should be thoroughly investigated, since this may inform future prevention strategies.

Previous studies including ours showed that down-regulation of REST, a master repressor of neuronal genes, is essential for NED of PCa cells under androgen withdraw [37] and IL-6 treatment [23]. In addition, a recent report showed that REST is down-regulated by miR under hypoxia condition [9]. These results indicate that REST may be a common key regulator for NED of PCa cells. Our transcriptome analysis comparing gene expression profiles of hypoxia and REST knockdown cells provides insight into function of REST in regulation of hypoxia up-regulated but not down-regulated genes (Figure 3A). GO analysis point to a role for REST in activation AMPK pathway (Figure 3B), a signaling pathway known for activating autophagy in androgen-dependent PCa cells subject to androgen withdraw, IL-6 treatment and hypoxia [23, 30]. Consistent with our bioinformatics predictions, our data here showed the first time that the AMPK pathway is activated by knockdown of REST (Figure 4A and 4B). Autophagy is a regulated degradation pathway that eukaryotic cells use to recycle cytoplasmic contents in response to starvation or stress. Previous studies including ours showed that autophagy is activated concomitantly with induction of NED by IL-6 treatment [22, 23]. Both reports also confirmed that autophagy activation is essential for IL-6-induced NED of PCa cells [22, 23]. In this study, we demonstrate that autophagy activation is also essential for hypoxia-induced NED of PCa cells. Pharmacological inhibition of autophagy by CQ, an autophagy inhibitor (Figure 7), or biochemical inhibition of autophagy by shRNA-mediated knockdown of autophagy-related genes (Figures 8 and 9) both reduced hypoxia-induced NED. Together, these data suggest that REST may function as a common factor responsible for NED induction by androgen withdrawal, IL-6 treatment and hypoxia. Consistently, GSEA analysis also pointed to the role for REST in PCa relapse (Figure 3C). It is worth noting that though our experiments were carried out in a cell culture model using LNCaP cells, which may not be fully representative of the clinical situation, the NED nature of this model cell line has long helped to elucidate the underlying mechanisms that are responsible for the development of NE tumor of PCa. In summary, the major finding of the present study, in combination with previous reports, is that REST mediated repression of AMPK/mTOR signaling and autophagy may be a commonly disrupted pathway for NED of PCa cells under different stimuli.

Protein levels of REST are mainly regulated by ubiquitylation mediated proteasome degradation [37, 42, 44]. This regulation was also confirmed in androgen deprivation-induced NED of PCa [37]. As mentioned earlier, a recent report showed that REST is also down-regulated at mRNA level by miR-106b~25 during hypoxia [9]. However, our data here showed that there is no reduction of REST at the RNA level after hypoxia treatment (Figure 1D). One potential explanation for this discrepancy is the choice of internal control. Several housekeeping genes commonly employed as RNA internal standards vary widely with hypoxia. In particular, GAPDH mRNA was significantly increased under hypoxia conditions [61]. These data suggest that β-TrCP is the major ubiquitin ligase that responsible for down-regulation of REST during NED of PCa. Since down-regulation of REST is responsible for signaling the NED of PCa, the development of β-TrCP specific inhibitors may provide a new strategy to increase the effectiveness of ADT treatment by prevention NED during ADT treatment. In addition, a recent report from Gluschnaider *et al.* showed that β-TrCP inhibition enhances androgen ablation-induced growth suppression of PCa cells [62]. There are more than 600 human ubiquitin E3 ligases and each of them has specificity toward certain target proteins [63]. This provides specificity and reduces off-target side effect of drug development. Collectively, these results reveal the potential of targeting β-TrCP as a novel combined therapeutic regime for NE tumors.

Tumor hypoxia is a common feature of PCa associated with poor prognosis and resistance to therapy. Recent discoveries showed that hypoxia is capable of inducing autophagy, a survival mechanism protecting cells from hypoxia-induced cell death [27]. This process is mediated either by HIF-1-dependent pathway involving BNIP3/BNIP3L [64] or HIF-1-independent pathway involving AMPK [47]. In PCa, the AMPK-mediated autophagy activation was beneficial for survival in androgen-dependent PCa cells under androgen deprivation and hypoxia conditions [30]. In addition, emerging evidence has shown that hypoxia is also capable of inducing NED of PCa [8, 9]. Moreover, recent reports including ours indicated that autophagy is essential for IL-6-induced NED [22, 23]. However, the role of autophagy in NED of PCa under hypoxia condition is still largely unknown. The most important finding of the present study highlights the essential role of autophagy in hypoxia-induced NED of PCa. Our data showed that inhibition of autophagy by either chemical inhibitor or (shRNA) against autophagy-related genes abolished hypoxia-induced NED (Figures 7–9). Together with our previous report [23], we suggest that autophagy is a mechanism essential for NED induced by both IL-6 and hypoxia and targeting autophagy may potentially be a useful strategy for treating NE tumors.

## MATERIALS AND METHODS

### Cell culture

LNCaP cells were cultured in RPMI 1640 (Gibco/Invitrogen, 31800–014) supplemented with 10% fetal bovine serum (FBS) (Hyclone, SH30071.03), 0.3 mg/ml L-glutamine (Sigma-Aldrich, G7513), and 100 units/ml Penicillin and 100 μg/ml Streptomycin (Gibco, 15140–122) and maintained at 37°C incubator containing 5% CO_2_ and 95% humidified air. LNCaP-TR-shREST, LNCaP-TR-REST, LNCaP-TR-shBeclin1, and LNCaP-TR-shAtg5 cell lines generated previously [23] were maintained as for LNCaP, but containing 5 μg/ml of blasticidin S (InvivoGen, ant-bl-1) and 50 μg/ml of zeocin. LNCaP-eGFP-LC3 generated in a previous study [46] were maintained as for LNCaP, but containing 400 μg/mL of G418 (Amresco, E859). For hypoxia treatment, LNCaP cells were seeded in 6 cm Petri dishes (Falcon) and maintained at 37°C in a humidified incubator containing 2% O_2_ atmosphere for 1 to 4 days.

### Fluorescent microscopy

LNCaP cells were plated on poly-L-lysine-coated coverslips (Marienfeld, 0111530) two days before subject to treatment. After the treatments as indicated, cells were fixed with 4% paraformaldehyde in PBS for 20 minutes. The coverslips were stained with Hoechst 33258 (Invitrogen, H3569), mounted in mounting solution [20 mM n-propylgallate, 20% PBS, and 80% glycerol], and visualized by Leica DMI4000B fluorescence microscope (Leica, DMI4000B). The neurite length images were visualized by phase-contrast optical microscope system with 40× lens and analyzed by MetaMorph (Molecular Devices, Neurite Outgrowth). The average neurite length of control cells cultured in normoxia condition (5% CO_2_−95% air) or without doxycycline (Dox) treatment that showed low level of NED in each experiment was used as 1. A comparison was made for neurite length obtained after hypoxia or Dox treatments. The eGFP-LC3 images were visualized by fluorescence system with 63x lens and analyzed by MetaMorph (Molecular Devices, Transflour). Autophagy-positive cells were identified as cells that contains more than 50 intense GFP aggregates of 5~7.5 μm and 15 intense GFP aggregates of 7.5~20 μm. LNCaP-eGFP-LC3 cells show low basic level of GFP aggregates under normoxic culture conditions.

### Immunoblotting and antibodies

Cells were lysed in ice-cold RIPA lysis buffer (Roche, 04693132001). Protein concentration was measured using Bradford assay dye reagent (Bio-Rad, 500–0006) with BSA as standard as described in manufacturer's protocol. For immunoblotting, equivalent protein amounts were loaded and separated on a 6%, 8%, 10% or 15% SDS-polyacrylamide gels. The separated proteins were transferred to PVDF membranes with 0.45 μm pore size (GE Healthcare, RPN303F). After transfer, the PVDF membranes were blocked with blocking solution [5% BSA in TBST]. Primary antibodies for p-mTOR (Ser2248) (Cell signaling, #2976), mTOR (Cell signaling, #2983), p-AMPK (Thr172) (Cell signaling, #2535), AMPK (Cell signaling, #2793), LC3B (Cell signaling, #2775), beclin1 (Cell signaling, #3738), Atg5 (Cell signaling, #2630), β-tubulin III (Cell signaling, #5666), neuron specific enolase (NSE) (GTX101553), β-TrCP (GTX102667), REST (Millipore, 09–019), and GAPDH (GeneTex, GTX100118) were diluted in blocking solution, probed to the membranes, treated with appropriate secondary antibody, visualized using a Pierce ECL Western Blotting Substrate (Thermo Scientific, 34080) and imaged via a Luminescence/Fluorescence Imaging System (FUJIFILM, LAS-4000).

### Reverse transcription (RT) and quantitative PCR (qPCR)

Total RNAs were isolated from non-induced and hypoxia-induced LNCaP cells using TRIzol reagent (Invitrogen, 15596–018). 2 μg of total RNA was reverse transcribed with Oligo(dT)_12–18_ using SuperScript^III^ RT (Invitrogen, 18080–085). The relative expression level of each gene was quantified by iQ^™^ SYBR Green Supermix (BioRad, BP170–8882AP) using Bio-Rad CFX96 Real-Time PCR Detection System. β-2-microglobulin (B2M) was used to normalize gene expression levels under hypoxia treatment. Gene expression with all other treatments was normalized against GAPDH.

### RNA-seq and gene ontology (GO) analysis

Total RNA harvested from hypoxia-treated LNCaP and Dox-treated LNCaP-TR-shREST cells were analyzed by high-throughput sequencing at the Sequencing Core of National Research Program for Genomic Medicine at National Yang-Ming University VYM Genome Research Center using an Illumina Genome Analyzer_II_. Sequencing reads were first trimmed by Bowtie (version 1.0.0) with default parameters and then the high quality reads were aligned to human reference genome hg19 using TopHat (version 2.0.8b) by Bowtie version 2.1.0 and samtools (version 0.1.9). Transcriptome information was obtained from Ensembl Release 70. The transcript abundances were calculated by fragments per kilobase of transcript per million mapped reads (FPKM) using Cufflinks version 2.1.1. Genes from all samples with FPKM > 0.05 were considered as expressed in cells and were used for further analysis. Differential expression of genes in hypoxia verses normoxia and REST knockdown verses control samples was analyzed by comparing FPKM. The biological functions of the gene expression profiles were analyzed by Ingenuity Pathway Analysis (IPA) software (http://www.ingenuity.com) using IPA spring release 2015.

## SUPPLEMENTARY MATERIALS FIGURES AND TABLES


