# Supplementary material for: REST reduction is essential for hypoxia-induced neuroendocrine differentiation of prostate cancer cells by activating autophagy signaling
Source: Oncotarget. 2016 Mar 28;7(18):26137–51. doi: 10.18632/oncotarget.8433 (PMC5041970; doi:10.18632/oncotarget.8433)
Supplement: Supplementary file 1 [file oncotarget-07-26137-s001.pdf]

REST reduction is essential for hypoxia-induced neuroendocrine differentiation of prostate cancer cells by activating autophagy signaling

Supplementary Materials

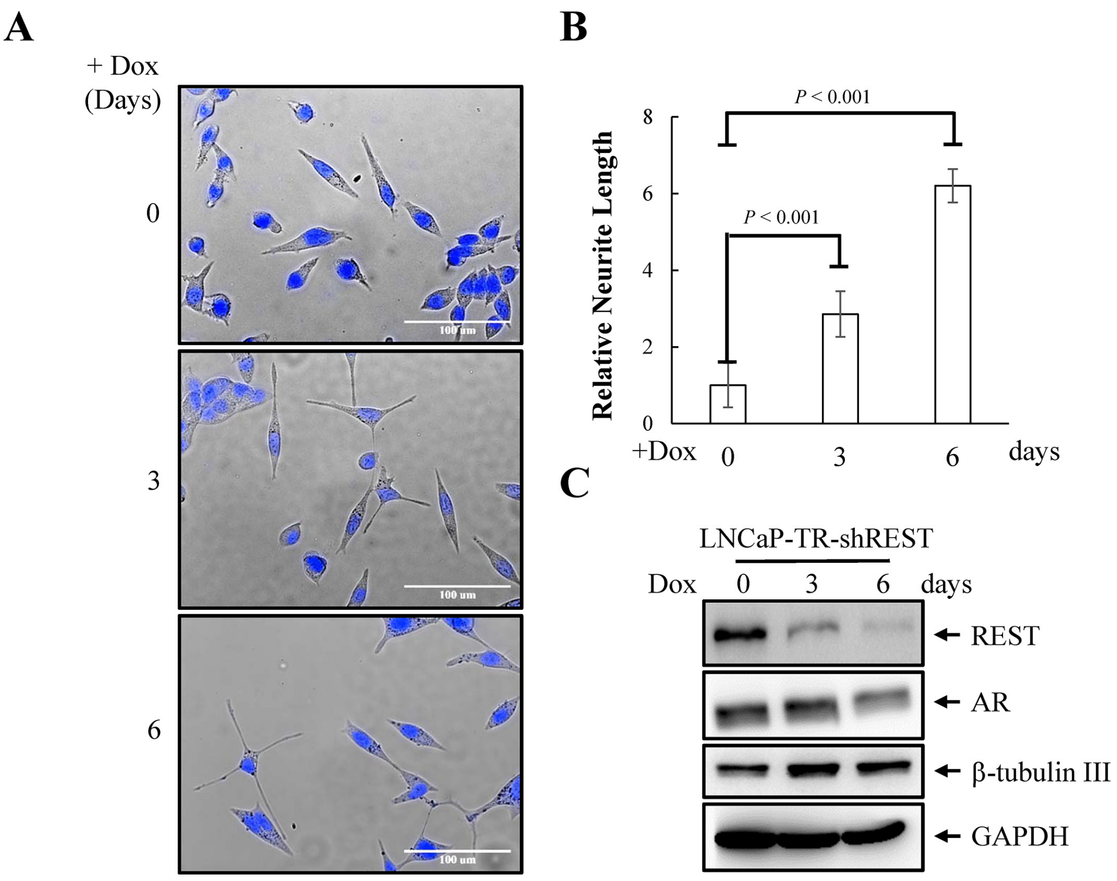

Supplementary Figure S1: REST down-regulation promotes NED progression. (**A**) LNCaP-TR-shREST cells were treated with 1 µg/ml Dox for 0, 3 and 6 days to knockdown REST. Representative photos of control and Dox-treated cells stained with Hoechst. (**B**) Neurite lengths were quantified by the average from 10 microscopic fields; bars, SD. (**C**) The expression of REST, AR and b-tubulin III in LNCaP-TR-shREST cells under Dox treatment as described in (A) was analyzed by immunoblotting. GAPDH was used as the loading control.

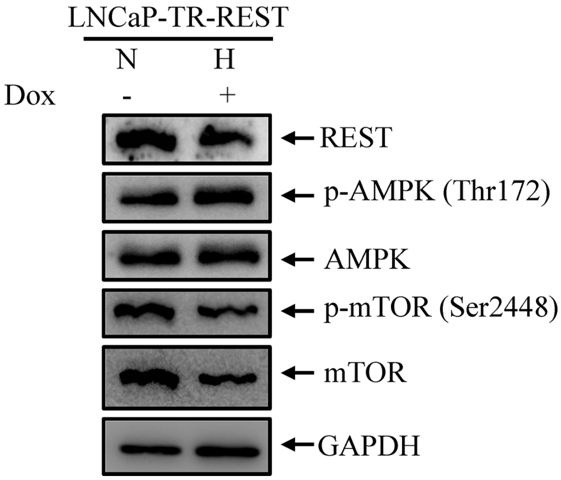

Supplementary Figure S2: Comparable phospho-AMPK and phospho-mTOR levels in hypoxia condition overexpressing REST and normoxia. Ectopic expression of REST during hypoxia restores phospho-AMPK and phospho-mTOR to normoxic levels. LNCaP-TR-REST cells were treated with 0.001 μg/ml Dox to overexpress REST in hypoxia for 3 days (right lane). Cells under normoxia conditions were also collected (left lane). TLCs were analyzed by immunoblotting using anti-REST, anti-p-AMPK, and anti-p-mTOR antibodies. Total AMPK and mTOR are used as controls. GAPDH was used as loading control.

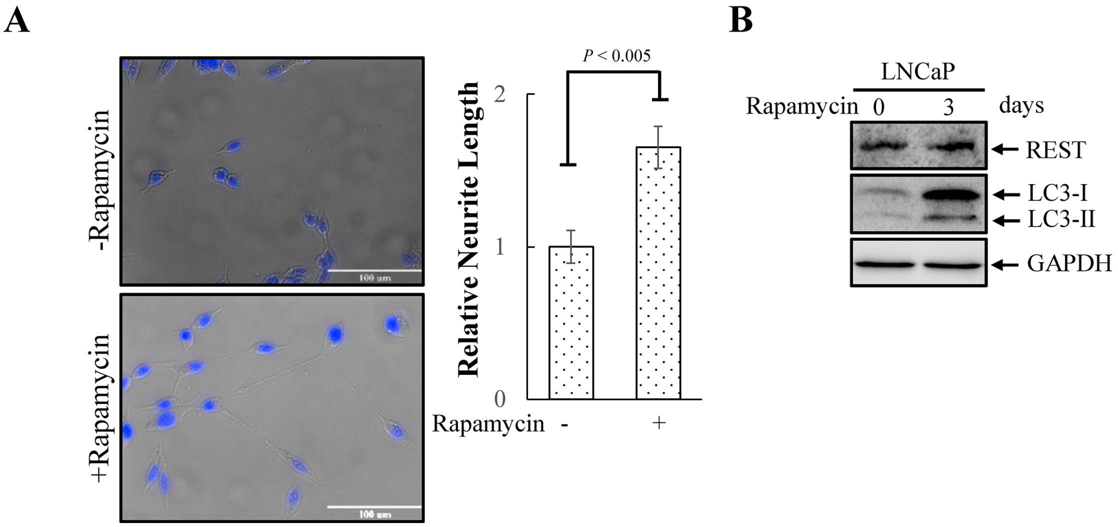

Supplementary Figure S3: Chemical inhibition of mTOR by rapamycin activated NED and autophagy but did not reduce REST expression in LNCaP cells. (**A**) LNCaP cells were treated with rapamycin for 3 days. Representative photos of control and rapamycin-treated cells were stained with Hoechst (left panel). The induced neurite length was assessed using brightfield microscopy images (40× magnification) and quantified by the average from 10 microscopic fields; bars, SD (right panel). (**B**) TLCs prepared from LNCaP cells treated as described in (A) were analyzed by immunoblotting using anti-REST and anti-LC3 antibodies. GAPDH was used as loading control.

Supplementary Table S1: Hypoxia up M

		LNCaP		
**Gene ID**	**Gene Symbol**	**10% FBS**	**Hypoxia**	**Fold change**
ENSG00000183044	ABAT	4.32	9.83	2.27
ENSG00000107331	ABCA2	7.35	11.20	1.52
ENSG00000115657	ABCB6	0.77	1.38	1.78
ENSG00000167315	ACAA2	0.78	2.22	2.86
ENSG00000131584	ACAP3	3.67	7.56	2.06
ENSG00000176244	ACBD7	1.99	4.45	2.23
ENSG00000114739	ACVR2B	2.13	3.72	1.75
ENSG00000119640	ACYP1	2.53	5.09	2.01
ENSG00000073670	ADAM11	1.55	2.64	1.70
ENSG00000154734	ADAMTS1	11.28	42.92	3.81
ENSG00000156218	ADAMTSL3	3.40	5.19	1.52
ENSG00000143382	ADAMTSL4	0.82	2.25	2.75
ENSG00000152990	GPR125	3.67	5.52	1.50
ENSG00000162688	AGL	4.43	6.84	1.54
ENSG00000134698	EIF2C4	8.28	15.88	1.92
ENSG00000155189	AGPAT5	19.14	47.77	2.50
ENSG00000186063	AIDA	45.73	70.86	1.55
ENSG00000162433	AK4	37.44	78.31	2.09
ENSG00000131016	AKAP12	0.89	39.69	44.80
ENSG00000127914	AKAP9	2.64	4.84	1.83
ENSG00000085662	AKR1B1	1.28	1.96	1.54
ENSG00000170017	ALCAM	32.16	52.79	1.64
ENSG00000149925	ALDOA	180.51	531.40	2.94
ENSG00000109107	ALDOC	13.42	51.27	3.82
ENSG00000139133	ALG10	3.83	6.95	1.81
ENSG00000175548	ALG10B	5.12	8.27	1.62
ENSG00000136383	ALPK3	2.38	7.40	3.11
ENSG00000242110	AMACR	12.11	33.69	2.78
ENSG00000176020	AMIGO3	0.00	1.62	1622541.30
ENSG00000145020	AMT	0.74	1.49	2.00
ENSG00000240038	AMY2B	0.79	1.23	1.57
ENSG00000110200	ANAPC15	1.54	2.62	1.70
ENSG00000091879	ANGPT2	3.49	33.97	9.72
ENSG00000166839	ANKDD1A	1.89	3.07	1.62
ENSG00000088448	ANKRD10	6.84	10.61	1.55
ENSG00000198720	ANKRD13B	5.40	9.24	1.71

ENSG00000163126	ANKRD23	0.47	1.15	2.45
ENSG00000135976	ANKRD36	0.89	1.96	2.21
ENSG00000186352	ANKRD37	3.48	11.36	3.26
ENSG00000163516	ANKZF1	9.81	28.69	2.93
ENSG00000011426	ANLN	6.12	16.90	2.76
ENSG00000146205	ANO7	0.60	1.96	3.28
ENSG00000182287	AP1S2	1.52	3.40	2.23
ENSG00000120868	APAF1	2.24	3.52	1.58
ENSG00000113108	APBB3	2.73	4.63	1.70
ENSG00000171388	APLN	4.72	41.36	8.76
ENSG00000105290	APLP1	14.56	29.76	2.04
ENSG00000198576	ARC	0.17	1.66	9.81
ENSG00000198826	ARHGAP11A	5.34	12.09	2.26
ENSG00000088756	ARHGAP28	2.65	7.94	3.00
ENSG00000004777	ARHGAP33	3.05	4.83	1.58
ENSG00000047648	ARHGAP6	6.22	11.13	1.79
ENSG00000242173	ARHGDIG	3.15	7.02	2.23
ENSG00000104728	ARHGEF10	3.35	7.16	2.14
ENSG00000240771	ARHGEF25	2.00	3.31	1.66
ENSG00000183111	ARHGEF37	9.40	19.17	2.04
ENSG00000137135	ARHGEF39	2.32	3.99	1.72
ENSG00000116017	ARID3A	3.75	7.34	1.96
ENSG00000179361	ARID3B	1.98	3.31	1.67
ENSG00000196843	ARID5A	7.39	11.92	1.61
ENSG00000172379	ARNT2	2.96	4.48	1.51
ENSG00000250151	ARPC4-TTLL3	5.86	10.55	1.80
ENSG00000105011	ASF1B	8.16	18.22	2.23
ENSG00000066279	ASPM	0.59	1.92	3.24
ENSG00000156802	ATAD2	4.84	10.42	2.15
ENSG00000176208	ATAD5	1.11	3.46	3.10
ENSG00000137343	ATAT1	4.11	8.44	2.06
ENSG00000101974	ATP11C	2.29	4.83	2.11
ENSG00000169020	ATP5I	0.96	2.88	3.01
ENSG00000143515	ATP8B2	2.42	4.73	1.96
ENSG00000085224	ATRX	4.82	8.41	1.75
ENSG00000178999	AURKB	3.24	7.34	2.26
ENSG00000158321	AUTS2	0.78	1.49	1.91
ENSG00000182272	B4GALNT4	20.07	30.63	1.53
ENSG00000043039	BARX2	0.36	1.01	2.79

ENSG00000103507	BCKDK	22.52	36.76	1.63
ENSG00000161267	BDH1	6.23	9.86	1.58
ENSG00000145734	BDP1	17.94	38.28	2.13
ENSG00000134107	BHLHE40	15.98	68.90	4.31
ENSG00000023445	BIRC3	1.28	1.99	1.56
ENSG00000089685	BIRC5	17.46	33.17	1.90
ENSG00000197299	BLM	0.86	2.04	2.38
ENSG00000138696	BMPR1B	12.76	22.83	1.79
ENSG00000102010	BMX	0.37	1.68	4.57
ENSG00000176171	BNIP3	49.55	244.89	4.94
ENSG00000104765	BNIP3L	48.02	145.80	3.04
ENSG00000012048	BRCA1	2.65	5.30	2.00
ENSG00000139618	BRCA2	0.87	2.61	2.99
ENSG00000136492	BRIP1	0.76	2.79	3.66
ENSG00000165288	BRWD3	1.15	1.95	1.70
ENSG00000133639	BTG1	192.29	304.90	1.59
ENSG00000159388	BTG2	22.54	37.08	1.64
ENSG00000169679	BUB1	1.33	2.29	1.73
ENSG00000156970	BUB1B	7.84	21.23	2.71
ENSG00000173088	C10orf131	0.65	1.12	1.71
ENSG00000168005	C11orf84	10.24	15.94	1.56
ENSG00000227051	C14orf132	1.81	4.77	2.64
ENSG00000162062	C16orf59	3.93	7.00	1.78
ENSG00000125319	C17orf53	0.94	1.46	1.56
ENSG00000214226	C17orf67	1.35	2.06	1.53
ENSG00000132016	C19orf57	0.71	2.07	2.94
ENSG00000130813	C19orf66	3.11	4.66	1.50
ENSG00000131591	C1orf159	1.60	2.92	1.82
ENSG00000118292	C1orf54	2.11	3.28	1.56
ENSG00000082196	C1QTNF3	0.67	3.27	4.85
ENSG00000133466	C1QTNF6	1.69	3.14	1.86
ENSG00000205863	C1QTNF9B	1.21	1.94	1.61
ENSG00000159403	C1R	0.95	1.62	1.71
ENSG00000160226	C21orf2	2.88	5.16	1.79
ENSG00000160298	C21orf58	2.14	4.79	2.24
ENSG00000172478	C2orf54	0.12	1.10	9.48
ENSG00000204128	C2orf72	6.62	17.93	2.71
ENSG00000042304	C2orf83	0.48	1.13	2.35
ENSG00000181744	C3orf58	3.32	8.28	2.50

ENSG00000123843	C4BPB	0.83	1.41	1.70
ENSG00000164096	C4orf3	74.40	218.75	2.94
ENSG00000205208	C4orf46	3.67	9.27	2.52
ENSG00000205129	C4orf47	0.63	2.75	4.38
ENSG00000134830	GPR77	0.75	1.16	1.55
ENSG00000204420	C6orf25	0.71	1.47	2.08
ENSG00000248801	RP11–664D7.4	1.46	3.16	2.16
ENSG00000241852	C8orf58	1.16	2.10	1.81
ENSG00000230185	C9orf147	0.44	1.02	2.30
ENSG00000063180	CA11	3.14	4.97	1.58
ENSG00000074410	CA12	4.32	9.43	2.18
ENSG00000107159	CA9	0.00	1.95	1952284.70
ENSG00000167535	CACNB3	3.48	7.06	2.03
ENSG00000129007	CALML4	4.93	8.21	1.67
ENSG00000058404	CAMK2B	1.94	4.19	2.16
ENSG00000153048	CARHSP1	8.92	15.95	1.79
ENSG00000137812	CASC5	1.45	5.58	3.86
ENSG00000166762	CATSPER2	1.65	2.52	1.53
ENSG00000141668	CBLN2	0.38	1.83	4.88
ENSG00000108468	CBX1	35.19	54.98	1.56
ENSG00000173894	CBX2	8.67	21.26	2.45
ENSG00000094916	CBX5	23.52	40.79	1.73
ENSG00000135736	CCDC102A	1.68	2.53	1.51
ENSG00000175455	CCDC14	4.40	7.63	1.74
ENSG00000149548	CCDC15	1.47	2.67	1.82
ENSG00000198865	CCDC152	16.90	28.28	1.67
ENSG00000122483	CCDC18	1.25	2.15	1.71
ENSG00000213213	KIAA1984	1.39	4.12	2.95
ENSG00000163040	CCDC74A	0.45	1.04	2.31
ENSG00000120647	CCDC77	3.46	5.61	1.62
ENSG00000186166	CCDC84	7.92	12.55	1.58
ENSG00000015133	CCDC88C	5.03	7.79	1.55
ENSG00000151882	CCL28	0.63	1.07	1.71
ENSG00000175305	CCNE2	0.52	1.59	3.07
ENSG00000162063	CCNF	4.23	7.40	1.75
ENSG00000138764	CCNG2	23.42	62.63	2.67
ENSG00000163660	CCNL1	7.40	11.96	1.62
ENSG00000103540	CCP110	3.46	9.35	2.70
ENSG00000154429	CCSAP	4.96	8.21	1.66

ENSG00000174950	CD164L2	0.53	1.04	1.96
ENSG00000120217	CD274	1.07	1.96	1.84
ENSG00000167775	CD320	21.28	32.63	1.53
ENSG00000104894	CD37	1.81	5.43	3.00
ENSG00000164045	CDC25A	2.72	5.61	2.06
ENSG00000093009	CDC45	6.85	13.72	2.00
ENSG00000094804	CDC6	13.43	20.95	1.56
ENSG00000097046	CDC7	5.58	10.79	1.93
ENSG00000184661	CDCA2	3.66	7.61	2.08
ENSG00000111665	CDCA3	3.24	6.28	1.93
ENSG00000170779	CDCA4	8.28	17.45	2.11
ENSG00000146670	CDCA5	7.50	16.10	2.15
ENSG00000144354	CDCA7	4.10	8.84	2.15
ENSG00000134690	CDCA8	12.93	20.57	1.59
ENSG00000139880	CDH24	1.08	2.46	2.27
ENSG00000170312	CDK1	20.16	57.29	2.84
ENSG00000058091	CDK14	6.89	11.49	1.67
ENSG00000117266	CDK18	0.41	1.13	2.74
ENSG00000123374	CDK2	10.92	27.76	2.54
ENSG00000176749	CDK5R1	4.33	11.97	2.77
ENSG00000136861	CDK5RAP2	2.93	4.48	1.53
ENSG00000129757	CDKN1C	1.08	4.78	4.44
ENSG00000123080	CDKN2C	5.25	11.13	2.12
ENSG00000129355	CDKN2D	8.00	12.89	1.61
ENSG00000167513	CDT1	9.41	17.90	1.90
ENSG00000186567	CEACAM19	0.90	1.38	1.53
ENSG00000099954	CECR2	0.72	1.24	1.73
ENSG00000143126	CELSR2	8.07	12.73	1.58
ENSG00000145241	CENPC1	0.84	1.48	1.77
ENSG00000138778	CENPE	0.38	1.08	2.84
ENSG00000117724	CENPF	2.94	10.60	3.61
ENSG00000153044	CENPH	15.03	26.26	1.75
ENSG00000102384	CENPI	1.52	4.37	2.88
ENSG00000151849	CENPJ	0.75	1.44	1.93
ENSG00000123219	CENPK	1.18	3.21	2.71
ENSG00000138092	CENPO	5.20	8.63	1.66
ENSG00000188312	CENPP	2.23	3.47	1.55
ENSG00000151725	MLF1IP	4.92	9.94	2.02
ENSG00000166582	CENPV	4.37	6.87	1.57

ENSG00000174799	CEP135	1.13	1.77	1.56
ENSG00000103995	CEP152	0.86	1.50	1.75
ENSG00000126001	CEP250	9.30	15.31	1.65
ENSG00000166004	KIAA1731	2.92	5.21	1.78
ENSG00000138180	CEP55	8.05	12.12	1.51
ENSG00000114107	CEP70	2.26	3.47	1.53
ENSG00000112877	CEP72	1.97	3.76	1.91
ENSG00000148019	CEP78	2.38	4.01	1.68
ENSG00000172824	CES4A	0.76	1.24	1.63
ENSG00000206530	WDR52	0.47	1.03	2.21
ENSG00000105792	C7orf63	3.82	9.87	2.58
ENSG00000167670	CHAF1A	12.94	20.19	1.56
ENSG00000159259	CHAF1B	5.25	8.20	1.56
ENSG00000171316	CHD7	2.26	3.43	1.52
ENSG00000149554	CHEK1	5.56	9.24	1.66
ENSG00000183765	CHEK2	1.90	3.07	1.62
ENSG00000159208	C1orf51	3.26	10.75	3.30
ENSG00000114737	CISH	0.63	1.16	1.83
ENSG00000122966	CIT	0.85	2.21	2.59
ENSG00000164442	CITED2	27.57	45.68	1.66
ENSG00000169607	CKAP2L	1.95	3.87	1.98
ENSG00000166165	CKB	120.14	268.04	2.23
ENSG00000254788	CKLF-CMTM1	0.44	1.32	2.99
ENSG00000157224	CLDN12	33.02	58.36	1.77
ENSG00000069493	CLEC2D	0.99	1.83	1.85
ENSG00000163815	CLEC3B	0.24	1.32	5.57
ENSG00000172243	CLEC7A	1.12	1.73	1.55
ENSG00000106665	CLIP2	0.54	1.18	2.16
ENSG00000105270	CLIP3	0.64	1.02	1.59
ENSG00000113240	CLK4	2.05	3.13	1.52
ENSG00000092853	CLSPN	0.76	2.29	3.02
ENSG00000120885	CLU	2.13	4.73	2.22
ENSG00000089505	CMTM1	0.49	1.13	2.32
ENSG00000174871	CNIH2	0.45	2.36	5.21
ENSG00000149970	CNKSR2	0.95	3.00	3.16
ENSG00000153721	CNKSR3	1.35	2.35	1.74
ENSG00000119946	CNNM1	1.96	3.70	1.89
ENSG00000119397	CNTRL	0.49	1.29	2.63
ENSG00000144810	COL8A1	0.73	1.21	1.66

ENSG00000196167	C11orf92	22.80	79.47	3.49
ENSG00000158270	COLEC12	6.54	14.99	2.29
ENSG00000135469	COQ10A	4.71	8.05	1.71
ENSG00000140848	CPNE2	2.52	4.15	1.65
ENSG00000178773	CPNE7	11.01	19.50	1.77
ENSG00000080819	CPOX	6.20	14.24	2.30
ENSG00000182809	CRIP2	3.73	9.38	2.51
ENSG00000072832	CRMP1	2.08	3.22	1.55
ENSG00000109846	CRYAB	1.13	11.37	10.05
ENSG00000114646	CSPG5	2.93	6.13	2.09
ENSG00000119326	CTNNAL1	3.90	6.57	1.68
ENSG00000162438	CTRC	0.75	1.47	1.97
ENSG00000143387	CTSK	1.51	3.14	2.08
ENSG00000107562	CXCL12	0.05	1.19	25.13
ENSG00000121966	CXCR4	0.07	5.12	72.11
ENSG00000166394	CYB5R2	0.57	1.53	2.69
ENSG00000108242	CYP2C18	1.14	1.94	1.71
ENSG00000138109	CYP2C9	4.31	6.50	1.51
ENSG00000073067	CYP2W1	0.66	1.07	1.61
ENSG00000036530	CYP46A1	0.64	1.01	1.58
ENSG00000142871	CYR61	3.62	13.59	3.75
ENSG00000035664	DAPK2	0.83	1.51	1.82
ENSG00000115866	DARS	13.26	21.77	1.64
ENSG00000006634	DBF4	4.18	6.93	1.66
ENSG00000156136	DCK	15.27	26.04	1.71
ENSG00000118655	DCLRE1B	3.82	6.52	1.71
ENSG00000134574	DDB2	21.20	31.89	1.50
ENSG00000085788	DDHD2	6.02	9.72	1.61
ENSG00000165490	C11orf82	1.82	3.35	1.84
ENSG00000145358	DDIT4L	0.59	1.73	2.92
ENSG00000013573	DDX11	5.47	10.53	1.93
ENSG00000067048	DDX3Y	15.33	35.33	2.30
ENSG00000024526	DEPDC1	1.81	4.14	2.28
ENSG00000035499	DEPDC1B	2.51	5.19	2.07
ENSG00000099958	DERL3	0.93	4.16	4.47
ENSG00000204311	DFNB59	0.52	1.17	2.24
ENSG00000128191	DGCR8	10.22	15.59	1.53
ENSG00000065357	DGKA	0.59	1.01	1.72
ENSG00000104808	DHDH	1.53	2.75	1.80

ENSG00000162496	DHRS3	9.25	14.07	1.52
ENSG00000139734	DIAPH3	1.21	1.85	1.53
ENSG00000211452	DIO1	2.52	6.68	2.65
ENSG00000197406	DIO3	3.19	37.72	11.82
ENSG00000176490	DIRAS1	6.12	10.09	1.65
ENSG00000126787	DLGAP5	2.15	5.27	2.46
ENSG00000144355	DLX1	1.13	2.15	1.91
ENSG00000197587	DMBX1	0.88	1.60	1.82
ENSG00000138346	DNA2	0.94	2.75	2.94
ENSG00000039139	DNAH5	0.86	1.35	1.56
ENSG00000137094	DNAJB5	2.17	4.23	1.95
ENSG00000178401	DNAJC22	5.25	8.61	1.64
ENSG00000213918	DNASE1	7.47	11.87	1.59
ENSG00000167968	DNASE1L2	1.28	1.98	1.54
ENSG00000106976	DNM1	0.71	1.33	1.87
ENSG00000115325	DOK1	1.31	2.24	1.71
ENSG00000146094	DOK3	0.87	1.71	1.98
ENSG00000159147	DONSON	1.62	2.76	1.70
ENSG00000092964	DPYSL2	2.17	6.48	2.98
ENSG00000113657	DPYSL3	0.76	1.73	2.29
ENSG00000151640	DPYSL4	3.87	24.56	6.35
ENSG00000134755	DSC2	21.49	42.87	1.99
ENSG00000136982	DSCC1	3.75	6.99	1.87
ENSG00000149636	DSN1	3.97	6.98	1.76
ENSG00000096696	DSP	32.91	111.15	3.38
ENSG00000143476	DTL	3.83	8.51	2.22
ENSG00000134769	DTNA	0.31	1.51	4.81
ENSG00000178498	DTX3	0.71	1.70	2.40
ENSG00000120875	DUSP4	21.71	40.40	1.86
ENSG00000138166	DUSP5	2.43	3.93	1.62
ENSG00000130829	DUSP9	3.62	7.81	2.16
ENSG00000007968	E2F2	1.19	2.66	2.24
ENSG00000165891	E2F7	1.52	4.87	3.21
ENSG00000129173	E2F8	0.71	3.17	4.43
ENSG00000088881	EBF4	0.65	1.66	2.57
ENSG00000114346	ECT2	7.17	17.98	2.51
ENSG00000127129	EDN2	0.95	2.37	2.49
ENSG00000102189	EEA1	2.31	3.76	1.63
ENSG00000172421	EFCAB3	0.93	3.20	3.43

ENSG00000143590	EFNA3	22.65	48.09	2.12
ENSG00000184349	EFNA5	31.65	57.88	1.83
ENSG00000146648	EGFR	10.96	47.58	4.34
ENSG00000135766	EGLN1	12.93	25.33	1.96
ENSG00000129521	EGLN3	1.18	14.04	11.93
ENSG00000161960	EIF4A1	1.44	2.27	1.58
ENSG00000156976	EIF4A2	76.17	119.79	1.57
ENSG00000102034	ELF4	1.39	2.15	1.54
ENSG00000166897	ELFN2	0.83	1.28	1.54
ENSG00000154920	EME1	2.78	5.34	1.92
ENSG00000213853	EMP2	8.32	13.73	1.65
ENSG00000074800	ENO1	122.04	343.37	2.81
ENSG00000111674	ENO2	5.52	65.10	11.79
ENSG00000120658	ENOX1	1.45	2.93	2.02
ENSG00000154269	ENPP3	0.50	1.30	2.62
ENSG00000054179	ENTPD2	2.49	6.19	2.48
ENSG00000079819	EPB41L2	0.70	1.42	2.04
ENSG00000196411	EPHB4	6.28	11.17	1.78
ENSG00000186871	ERCC6L	1.36	4.93	3.63
ENSG00000197930	ERO1L	5.25	23.39	4.46
ENSG00000171320	ESCO2	2.69	5.43	2.02
ENSG00000135476	ESPL1	1.91	5.61	2.94
ENSG00000187017	ESPN	0.59	1.48	2.52
ENSG00000158220	ESYT3	1.59	2.57	1.62
ENSG00000157557	ETS2	2.21	4.19	1.90
ENSG00000166979	EVA1C	0.71	1.52	2.16
ENSG00000072840	EVC	7.40	11.66	1.58
ENSG00000196405	EVL	6.77	12.41	1.83
ENSG00000174371	EXO1	2.05	4.38	2.13
ENSG00000181104	F2R	7.24	13.93	1.92
ENSG00000170231	FABP6	1.86	2.92	1.57
ENSG00000134824	FADS2	57.78	118.24	2.05
ENSG00000144199	FAHD2B	1.16	2.24	1.93
ENSG00000189057	FAM111B	3.56	13.48	3.79
ENSG00000185519	FAM131C	0.49	1.04	2.14
ENSG00000138640	FAM13A	6.00	12.51	2.08
ENSG00000114023	FAM162A	57.17	149.73	2.62
ENSG00000161682	FAM171A2	0.55	1.80	3.25
ENSG00000197520	FAM177B	0.44	3.61	8.16

ENSG00000189350	FAM179A	0.72	1.21	1.68
ENSG00000186973	FAM183A	1.23	1.86	1.51
ENSG00000122378	FAM213A	6.41	9.81	1.53
ENSG00000188732	FAM221A	3.53	5.67	1.61
ENSG00000184949	FAM227A	1.69	3.05	1.81
ENSG00000167695	FAM57A	18.50	34.06	1.84
ENSG00000129195	FAM64A	6.67	16.90	2.54
ENSG00000188610	FAM72B	5.78	8.73	1.51
ENSG00000176973	FAM89B	30.30	46.29	1.53
ENSG00000188343	FAM92A1	0.61	1.85	3.04
ENSG00000158169	FANCC	2.01	3.80	1.89
ENSG00000144554	FANCD2	5.11	10.80	2.11
ENSG00000140525	FANCI	6.95	19.55	2.81
ENSG00000187790	FANCM	0.95	1.83	1.92
ENSG00000152767	FARP1	1.33	2.05	1.55
ENSG00000141665	FBXO15	0.87	1.38	1.58
ENSG00000214050	FBXO16	2.55	4.64	1.82
ENSG00000112029	FBXO5	2.95	5.90	2.00
ENSG00000168496	FEN1	21.52	45.89	2.13
ENSG00000163497	FEV	1.55	2.70	1.75
ENSG00000126262	FFAR2	0.75	1.97	2.63
ENSG00000129682	FGF13	2.68	4.40	1.64
ENSG00000077782	FGFR1	0.67	1.44	2.14
ENSG00000132436	FIGNL1	5.83	11.71	2.01
ENSG00000079150	FKBP7	1.11	2.40	2.16
ENSG00000115414	FN1	3.85	12.90	3.35
ENSG00000141560	FN3KRP	12.86	19.81	1.54
ENSG00000137942	FNBP1L	20.84	32.12	1.54
ENSG00000170345	FOS	0.64	7.09	11.09
ENSG00000075426	FOSL2	6.91	12.37	1.79
ENSG00000111206	FOXM1	13.90	24.66	1.78
ENSG00000073910	FRY	2.00	3.15	1.57
ENSG00000075539	FRYL	1.19	2.43	2.03
ENSG00000075618	FSCN1	3.01	18.17	6.04
ENSG00000196968	FUT11	5.30	28.48	5.38
ENSG00000104290	FZD3	3.19	5.49	1.72
ENSG00000092140	G2E3	5.00	7.69	1.54
ENSG00000109458	GAB1	1.32	2.03	1.54
ENSG00000204681	GABBR1	1.55	2.33	1.51

ENSG00000099860	GADD45B	2.64	17.73	6.73
ENSG00000130222	GADD45G	16.52	25.19	1.52
ENSG00000108479	GALK1	4.66	8.01	1.72
ENSG00000111640	GAPDH	727.54	1606.61	2.21
ENSG00000136895	GARNL3	1.33	2.82	2.12
ENSG00000139354	GAS2L3	1.27	2.77	2.18
ENSG00000114480	GBE1	7.96	13.27	1.67
ENSG00000137880	GCHFR	25.69	39.03	1.52
ENSG00000001084	GCLC	1.96	2.98	1.52
ENSG00000187210	GCNT1	10.63	24.44	2.30
ENSG00000135414	GDF11	7.09	12.66	1.79
ENSG00000130513	GDF15	727.99	1434.35	1.97
ENSG00000178295	GEN1	2.04	3.71	1.81
ENSG00000137563	GGH	2.99	4.66	1.56
ENSG00000101003	GINS1	8.36	21.66	2.59
ENSG00000131153	GINS2	3.10	5.50	1.77
ENSG00000147536	GINS4	3.90	7.23	1.85
ENSG00000182963	GJC1	1.08	2.19	2.02
ENSG00000102393	GLA	3.76	7.06	1.88
ENSG00000149328	GLB1L2	6.98	11.74	1.68
ENSG00000156689	GLYATL2	2.56	4.39	1.71
ENSG00000185838	GNB1L	0.88	1.38	1.58
ENSG00000168243	GNG4	1.20	2.82	2.35
ENSG00000184206	RP11–671M22.1	0.87	1.30	1.50
ENSG00000175265	GOLGA8A	7.67	25.83	3.37
ENSG00000215252	GOLGA8B	5.05	8.49	1.68
ENSG00000063660	GPC1	4.14	6.28	1.52
ENSG00000213420	GPC2	1.27	3.47	2.74
ENSG00000147257	GPC3	2.23	4.72	2.12
ENSG00000164850	GPER	3.53	12.41	3.52
ENSG00000105220	GPI	102.92	381.99	3.71
ENSG00000136235	GPNMB	5.11	7.88	1.54
ENSG00000077585	GPR137B	8.99	14.59	1.62
ENSG00000164849	GPR146	0.69	3.17	4.59
ENSG00000163328	GPR155	1.56	2.56	1.63
ENSG00000171657	GPR82	0.65	1.06	1.65
ENSG00000204175	GPRIN2	14.25	22.50	1.58
ENSG00000160360	GPSM1	11.32	18.87	1.67
ENSG00000167701	GPT	1.29	2.41	1.87

ENSG00000176884	GRIN1	2.47	4.32	1.75
ENSG00000198785	GRIN3A	0.39	2.18	5.54
ENSG00000148180	GSN	1.76	2.91	1.66
ENSG00000134202	GSTM3	6.98	11.09	1.59
ENSG00000099984	GSTT2	2.08	5.26	2.53
ENSG00000133433	GSTT2B	1.44	3.75	2.61
ENSG00000145736	GTF2H2	6.88	10.95	1.59
ENSG00000213780	GTF2H4	0.26	3.00	11.32
ENSG00000075218	GTSE1	5.48	13.16	2.40
ENSG00000188486	H2AFX	86.91	144.85	1.67
ENSG00000138796	HADH	35.53	61.73	1.74
ENSG00000214367	HAUS3	3.09	5.31	1.72
ENSG00000147874	HAUS6	4.32	7.53	1.74
ENSG00000119969	HELLS	1.52	4.01	2.63
ENSG00000069812	HES2	1.46	2.85	1.96
ENSG00000144485	HES6	55.19	98.59	1.79
ENSG00000179111	HES7	0.28	1.14	4.13
ENSG00000135245	HILPDA	13.83	35.34	2.55
ENSG00000123485	HJURP	3.62	9.26	2.56
ENSG00000159399	HK2	16.29	74.27	4.56
ENSG00000206503	HLA-A	40.33	62.10	1.54
ENSG00000204632	HLA-G	0.65	1.55	2.38
ENSG00000071794	HLTF	16.54	28.65	1.73
ENSG00000164104	HMGB2	26.82	56.95	2.12
ENSG00000029993	HMGB3	12.72	22.99	1.81
ENSG00000072571	HMMR	11.67	21.39	1.83
ENSG00000100292	HMOX1	22.56	35.53	1.57
ENSG00000138668	HNRNPD	14.93	25.19	1.69
ENSG00000152795	HNRPDL	15.26	28.51	1.87
ENSG00000051128	HOMER3	2.56	4.17	1.63
ENSG00000106004	HOXA5	1.98	3.01	1.52
ENSG00000106006	HOXA6	1.05	1.92	1.82
ENSG00000115756	HPCAL1	6.52	11.94	1.83
ENSG00000164120	HPGD	0.93	1.70	1.82
ENSG00000165704	HPRT1	30.54	57.82	1.89
ENSG00000176387	HSD11B2	20.31	45.46	2.24
ENSG00000087076	HSD17B14	0.48	1.60	3.30
ENSG00000025423	HSD17B6	0.74	1.32	1.79
ENSG00000102878	HSF4	3.23	5.26	1.63

ENSG00000080824	HSP90AA1	137.24	224.15	1.63
ENSG00000204388	HSPA1B	60.12	103.19	1.72
ENSG00000204389	HSPA1A	47.19	82.03	1.74
ENSG00000106211	HSPB1	269.43	458.14	1.70
ENSG00000081870	HSPB11	4.50	7.38	1.64
ENSG00000152137	HSPB8	1.73	2.91	1.68
ENSG00000120694	HSPH1	12.74	21.69	1.70
ENSG00000157219	HTR5A	0.03	2.42	73.90
ENSG00000220575	AC093726.4	0.29	3.27	11.34
ENSG00000125968	ID1	3.07	10.18	3.32
ENSG00000117318	ID3	5.06	9.83	1.94
ENSG00000182054	IDH2	26.13	39.49	1.51
ENSG00000140443	IGF1R	7.38	11.98	1.62
ENSG00000126246	IGFLR1	1.60	2.96	1.85
ENSG00000085552	IGSF9	2.16	4.74	2.20
ENSG00000080854	IGSF9B	0.68	1.20	1.76
ENSG00000166130	IKBIP	1.04	2.17	2.09
ENSG00000124391	IL17C	0.34	1.30	3.81
ENSG00000056736	IL17RB	4.78	8.09	1.69
ENSG00000136689	IL1RN	2.12	4.64	2.19
ENSG00000008517	IL32	1.08	4.04	3.74
ENSG00000136695	IL36RN	1.30	2.27	1.75
ENSG00000149503	INCENP	8.65	17.25	1.99
ENSG00000125629	INSIG2	7.71	22.54	2.92
ENSG00000198700	IPO9	13.00	20.01	1.54
ENSG00000160051	IQCC	3.70	5.90	1.59
ENSG00000169047	IRS1	2.66	4.17	1.57
ENSG00000105655	ISYNA1	2.71	5.24	1.93
ENSG00000096433	ITPR3	7.42	14.49	1.95
ENSG00000116679	IVNS1ABP	3.14	9.02	2.87
ENSG00000099840	IZUMO4	0.75	1.84	2.45
ENSG00000171988	JMJD1C	5.64	9.11	1.61
ENSG00000197256	KANK2	5.70	9.42	1.65
ENSG00000144445	KANSL1L	1.83	3.21	1.75
ENSG00000131398	KCNC3	1.32	3.63	2.76
ENSG00000184408	KCND2	0.33	1.81	5.39
ENSG00000171385	KCND3	2.24	4.33	1.93
ENSG00000055118	KCNH2	0.63	1.49	2.37
ENSG00000183960	KCNH8	0.52	1.19	2.29

ENSG00000053918	KCNQ1	2.61	5.15	1.97
ENSG00000089094	KDM2B	2.55	3.84	1.51
ENSG00000115548	KDM3A	3.43	14.20	4.14
ENSG00000127663	KDM4B	14.15	31.24	2.21
ENSG00000166803	KIAA0101	4.46	11.66	2.61
ENSG00000196123	KIAA0895L	8.32	15.91	1.91
ENSG00000109265	KIAA1211	1.52	2.94	1.93
ENSG00000163507	KIAA1524	0.86	1.53	1.76
ENSG00000122778	KIAA1549	3.11	4.79	1.54
ENSG00000174718	KIAA1551	2.64	5.42	2.05
ENSG00000130518	KIAA1683	0.84	1.67	1.98
ENSG00000144320	KIAA1715	3.68	8.30	2.26
ENSG00000165185	KIAA1958	2.41	4.08	1.69
ENSG00000116685	KIAA2013	43.85	82.48	1.88
ENSG00000163808	KIF15	1.06	4.89	4.63
ENSG00000186185	KIF18B	2.32	6.71	2.89
ENSG00000112984	KIF20A	7.50	19.14	2.55
ENSG00000138182	KIF20B	1.40	3.13	2.23
ENSG00000137807	KIF23	3.16	6.59	2.08
ENSG00000186638	KIF24	1.45	3.10	2.14
ENSG00000142945	KIF2C	9.98	16.37	1.64
ENSG00000084731	KIF3C	1.09	1.98	1.81
ENSG00000090889	KIF4A	5.95	11.90	2.00
ENSG00000168280	KIF5C	5.41	8.17	1.51
ENSG00000166813	KIF7	0.68	1.09	1.61
ENSG00000116014	KISS1R	0.21	11.89	57.12
ENSG00000049130	KITLG	0.22	1.08	4.81
ENSG00000136826	KLF4	6.11	11.98	1.96
ENSG00000114796	KLHL24	7.49	12.35	1.65
ENSG00000183655	KLHL25	3.86	6.00	1.55
ENSG00000174562	KLK15	1.60	3.53	2.21
ENSG00000167751	KLK2	22.23	134.93	6.07
ENSG00000142515	KLK3	747.53	1313.96	1.76
ENSG00000167749	KLK4	83.39	134.62	1.61
ENSG00000184445	KNTC1	2.56	7.53	2.94
ENSG00000182481	KPNA2	38.28	64.41	1.68
ENSG00000171345	KRT19	1.67	5.23	3.13
ENSG00000167767	KRT80	2.09	4.89	2.35
ENSG00000126777	KTN1	9.14	13.74	1.50

ENSG00000213626	LBH	0.12	1.01	8.34
ENSG00000184925	LCN12	0.31	1.08	3.50
ENSG00000188501	LCTL	0.71	1.09	1.53
ENSG00000134333	LDHA	89.28	476.41	5.34
ENSG00000164406	LEAP2	1.25	2.83	2.27
ENSG00000106003	LFNG	0.86	5.39	6.31
ENSG00000100097	LGALS1	0.28	1.83	6.46
ENSG00000128342	LIF	3.50	6.92	1.98
ENSG00000105486	LIG1	0.45	1.20	2.63
ENSG00000111052	LIN7A	1.55	3.13	2.02
ENSG00000082929	C4orf6	0.06	1.12	18.51
ENSG00000220008	LINGO3	0.36	1.07	2.98
ENSG00000113368	LMNB1	28.16	66.05	2.35
ENSG00000228804	RP11–211G3.3	1.36	2.08	1.53
ENSG00000257545	RP11–144F15.1	0.80	1.86	2.32
ENSG00000179240	RP11–111M22.2	0.77	1.44	1.87
ENSG00000005189	AC004381.6	4.13	6.88	1.66
ENSG00000175556	LONRF3	2.00	3.28	1.64
ENSG00000113083	LOX	2.93	15.87	5.41
ENSG00000176454	LPCAT4	1.78	3.13	1.76
ENSG00000132793	LPIN3	5.45	10.49	1.92
ENSG00000139263	LRIG3	1.93	3.33	1.73
ENSG00000123384	LRP1	1.96	3.99	2.04
ENSG00000109771	LRP2BP	0.70	1.50	2.13
ENSG00000100068	LRP5L	2.33	3.62	1.55
ENSG00000157193	LRP8	2.66	4.54	1.70
ENSG00000169683	LRRC45	4.10	7.89	1.93
ENSG00000133739	LRRCC1	2.04	3.36	1.65
ENSG00000007392	LUC7L	3.70	6.95	1.88
ENSG00000108848	LUC7L3	19.88	30.36	1.53
ENSG00000187398	LUZP2	5.55	8.62	1.55
ENSG00000183833	MAATS1	0.35	1.13	3.21
ENSG00000133315	MACROD1	3.55	6.20	1.75
ENSG00000164109	MAD2L1	4.03	10.49	2.60
ENSG00000185022	MAFF	1.18	5.06	4.28
ENSG00000154545	MAGED4	0.93	1.49	1.60
ENSG00000172175	MALT1	9.34	37.82	4.05
ENSG00000117643	MAN1C1	0.77	1.28	1.66
ENSG00000172469	MANEA	12.20	18.82	1.54

ENSG00000078018	MAP2	1.43	2.60	1.82
ENSG00000108984	MAP2K6	1.17	2.34	2.00
ENSG00000139625	MAP3K12	2.53	3.89	1.54
ENSG00000142733	MAP3K6	1.00	1.81	1.81
ENSG00000012983	MAP4K5	2.60	4.10	1.58
ENSG00000188130	MAPK12	4.94	7.88	1.60
ENSG00000181085	MAPK15	0.79	2.26	2.87
ENSG00000186868	MAPT	3.96	6.09	1.54
ENSG00000145416	41699	1.19	1.97	1.66
ENSG00000009724	MASP2	0.71	1.18	1.67
ENSG00000151224	MAT1A	0.89	1.60	1.80
ENSG00000143384	MCL1	70.21	107.22	1.53
ENSG00000065328	MCM10	2.45	7.56	3.08
ENSG00000073111	MCM2	17.20	42.21	2.45
ENSG00000112118	MCM3	6.83	13.31	1.95
ENSG00000104738	MCM4	13.95	31.93	2.29
ENSG00000100297	MCM5	7.97	14.13	1.77
ENSG00000076003	MCM6	5.64	8.92	1.58
ENSG00000166508	MCM7	12.52	25.69	2.05
ENSG00000125885	MCM8	5.38	9.44	1.75
ENSG00000137337	MDC1	11.52	18.78	1.63
ENSG00000110492	MDK	49.28	86.50	1.76
ENSG00000165304	MELK	9.90	18.78	1.90
ENSG00000176845	METRNL	3.50	6.55	1.87
ENSG00000185432	METTL7A	15.60	26.24	1.68
ENSG00000254726	MEX3A	3.17	12.88	4.07
ENSG00000183496	MEX3B	3.76	7.94	2.11
ENSG00000181588	MEX3D	12.40	33.45	2.70
ENSG00000140545	MFGE8	2.16	3.33	1.55
ENSG00000163975	MFI2	0.82	1.45	1.77
ENSG00000168389	MFSD2A	1.35	2.28	1.68
ENSG00000125871	C20orf72	14.98	24.65	1.65
ENSG00000135596	MICAL1	1.27	2.29	1.81
ENSG00000133816	MICAL2	3.57	7.04	1.97
ENSG00000243156	MICAL3	4.21	7.82	1.86
ENSG00000240972	MIF	599.86	1116.65	1.86
ENSG00000197182	FLJ27365	1.65	2.54	1.54
ENSG00000129534	MIS18BP1	1.68	3.12	1.86
ENSG00000148773	MKI67	5.48	16.01	2.92

ENSG00000196549	MME	73.53	114.49	1.56
ENSG00000123342	MMP19	0.06	1.34	21.14
ENSG00000146263	MMS22L	0.66	1.06	1.59
ENSG00000121211	MND1	3.98	6.94	1.74
ENSG00000138587	MNS1	2.26	3.77	1.67
ENSG00000105926	MPP6	4.32	6.56	1.52
ENSG00000149573	MPZL2	4.07	9.35	2.30
ENSG00000184350	MRGPRE	0.32	3.31	10.49
ENSG00000095002	MSH2	1.99	3.26	1.63
ENSG00000204410	MSH5	1.73	3.82	2.21
ENSG00000255152	MSH5-SAPCD1	2.08	4.58	2.21
ENSG00000135097	MSI1	2.39	4.22	1.76
ENSG00000173531	MST1	1.63	3.27	2.01
ENSG00000187193	MT1X	21.15	40.87	1.93
ENSG00000125148	MT2A	75.07	165.17	2.20
ENSG00000172167	MTBP	0.84	1.49	1.78
ENSG00000143033	MTF2	4.55	7.21	1.59
ENSG00000242114	MTFP1	7.79	21.83	2.80
ENSG00000146410	FAM54A	0.63	1.30	2.07
ENSG00000116984	MTR	7.74	12.29	1.59
ENSG00000176945	MUC20	1.34	3.01	2.25
ENSG00000119950	MXI1	19.92	39.42	1.98
ENSG00000101057	MYBL2	17.50	29.63	1.69
ENSG00000266714	MYO15B	0.73	1.23	1.70
ENSG00000157483	MYO1E	1.63	3.87	2.38
ENSG00000162601	MYSM1	6.76	11.87	1.75
ENSG00000170476	MZB1	1.45	2.97	2.05
ENSG00000172766	NAA16	3.75	5.83	1.55
ENSG00000138386	NAB1	1.70	3.15	1.86
ENSG00000249437	NAIP	2.87	6.76	2.35
ENSG00000141562	NARF	8.28	17.64	2.13
ENSG00000132780	NASP	16.52	31.59	1.91
ENSG00000134369	NAV1	3.66	9.15	2.50
ENSG00000010292	NCAPD2	24.84	40.73	1.64
ENSG00000151503	NCAPD3	3.81	6.39	1.68
ENSG00000109805	NCAPG	5.30	11.35	2.14
ENSG00000146918	NCAPG2	1.92	3.82	1.99
ENSG00000121152	NCAPH	5.49	13.61	2.48
ENSG00000184454	NCMAP	0.70	1.09	1.56

ENSG00000058804	TMEM48	9.58	15.85	1.65
ENSG00000080986	NDC80	8.03	13.58	1.69
ENSG00000104419	NDRG1	8.93	80.78	9.04
ENSG00000185633	NDUFA4L2	6.59	203.72	30.90
ENSG00000111859	NEDD9	0.93	2.22	2.38
ENSG00000140398	NEIL1	3.35	5.02	1.50
ENSG00000109674	NEIL3	2.24	4.76	2.13
ENSG00000117650	NEK2	6.22	10.73	1.73
ENSG00000166881	TMEM194A	6.59	11.49	1.74
ENSG00000173848	NET1	24.19	38.34	1.58
ENSG00000166342	NETO1	16.54	32.76	1.98
ENSG00000214357	NEURL1B	2.44	4.78	1.96
ENSG00000170322	NFRKB	10.73	18.88	1.76
ENSG00000257108	NHLRC4	0.92	2.23	2.43
ENSG00000084628	NKAIN1	0.20	1.23	6.26
ENSG00000114857	NKTR	8.32	14.40	1.73
ENSG00000167034	NKX3–1	168.57	256.94	1.52
ENSG00000169992	NLGN2	2.02	4.56	2.26
ENSG00000196338	NLGN3	0.81	1.67	2.07
ENSG00000140853	NLRC5	0.61	1.04	1.71
ENSG00000197696	NMB	2.65	8.46	3.19
ENSG00000053438	NNAT	0.94	2.37	2.51
ENSG00000164867	NOS3	8.69	16.68	1.92
ENSG00000074181	NOTCH3	3.03	6.08	2.01
ENSG00000255346	NOX5	1.23	1.87	1.53
ENSG00000149308	NPAT	2.11	3.69	1.74
ENSG00000183979	NPB	1.47	2.75	1.87
ENSG00000215440	NPEPL1	2.13	4.15	1.95
ENSG00000163273	NPPC	1.98	4.59	2.32
ENSG00000183971	NPW	2.97	5.00	1.68
ENSG00000143257	NR1I3	0.44	1.09	2.50
ENSG00000123358	NR4A1	3.10	6.00	1.94
ENSG00000185189	NRBP2	0.92	3.32	3.61
ENSG00000134986	NREP	4.46	14.86	3.33
ENSG00000154146	NRGN	2.31	3.98	1.72
ENSG00000137404	NRM	1.98	6.33	3.19
ENSG00000168824	NSG1	0.67	1.07	1.59
ENSG00000101188	NTSR1	0.73	1.30	1.77
ENSG00000106268	NUDT1	5.35	12.41	2.32

ENSG00000143228	NUF2	3.32	9.14	2.75
ENSG00000198088	NUP62CL	1.69	3.06	1.81
ENSG00000137804	NUSAP1	22.58	49.42	2.19
ENSG00000182379	NXPH4	35.25	70.12	1.99
ENSG00000111331	OAS3	3.27	5.61	1.71
ENSG00000154358	OBSCN	1.52	2.69	1.77
ENSG00000109205	ODAM	0.19	1.43	7.54
ENSG00000115758	ODC1	77.41	119.17	1.54
ENSG00000046651	OFD1	2.75	4.52	1.64
ENSG00000105088	OLFM2	0.87	2.92	3.37
ENSG00000180785	OR51E1	1.34	16.18	12.05
ENSG00000085840	ORC1	1.88	4.73	2.52
ENSG00000091039	OSBPL8	15.77	26.00	1.65
ENSG00000169914	OTUD3	4.08	6.64	1.63
ENSG00000180914	OXTR	0.47	1.12	2.40
ENSG00000090530	LEPREL1	0.45	1.09	2.44
ENSG00000122884	P4HA1	10.70	65.96	6.16
ENSG00000124507	PACSIN1	3.22	4.85	1.50
ENSG00000076641	PAG1	1.30	3.10	2.38
ENSG00000112941	PAPD7	9.84	15.26	1.55
ENSG00000162073	PAQR4	11.86	21.51	1.81
ENSG00000137819	PAQR5	0.61	1.08	1.75
ENSG00000178184	PARD6G	4.45	7.34	1.65
ENSG00000267270	AC139100.2	0.87	1.47	1.70
ENSG00000185480	PARPBP	4.17	6.60	1.58
ENSG00000159086	GCFC1	2.94	5.50	1.87
ENSG00000168078	PBK	6.45	16.03	2.49
ENSG00000197479	PCDHB11	1.14	1.82	1.60
ENSG00000132646	PCNA	30.32	54.50	1.80
ENSG00000106333	PCOLCE	0.41	1.18	2.86
ENSG00000174788	PCP2	0.32	1.10	3.49
ENSG00000099139	PCSK5	0.33	1.06	3.19
ENSG00000152256	PDK1	5.48	24.00	4.38
ENSG00000067992	PDK3	7.68	17.24	2.24
ENSG00000083642	PDS5B	8.10	13.50	1.67
ENSG00000241360	PDXP	13.56	21.18	1.56
ENSG00000067840	PDZD4	0.81	1.97	2.41
ENSG00000121440	PDZRN3	2.68	5.02	1.87
ENSG00000134020	PEBP4	0.33	1.82	5.45

ENSG00000179094	PER1	1.76	3.63	2.06
ENSG00000049246	PER3	4.06	6.66	1.64
ENSG00000170525	PFKFB3	4.16	57.22	13.77
ENSG00000114268	PFKFB4	2.09	22.40	10.70
ENSG00000141959	PFKL	18.84	31.42	1.67
ENSG00000067057	PFKP	24.51	126.89	5.18
ENSG00000171314	PGAM1	210.09	461.41	2.20
ENSG00000096088	PGC	0.88	1.70	1.93
ENSG00000102144	PGK1	111.95	657.28	5.87
ENSG00000079739	PGM1	25.95	46.47	1.79
ENSG00000130517	PGPEP1	13.61	20.69	1.52
ENSG00000130024	PHF10	8.70	16.65	1.91
ENSG00000078043	PIAS2	4.48	14.39	3.21
ENSG00000083535	PIBF1	0.67	1.05	1.56
ENSG00000177595	PIDD	2.46	3.77	1.54
ENSG00000140451	PIF1	1.07	1.85	1.73
ENSG00000137193	PIM1	2.56	8.23	3.22
ENSG00000102096	PIM2	7.99	13.92	1.74
ENSG00000067225	PKM	65.83	187.14	2.84
ENSG00000127564	PKMYT1	2.36	4.37	1.85
ENSG00000160447	PKN3	0.83	1.48	1.77
ENSG00000057294	PKP2	1.48	2.29	1.54
ENSG00000188257	PLA2G2A	4.45	24.23	5.45
ENSG00000159337	PLA2G4D	0.19	7.37	38.01
ENSG00000124181	PLCG1	12.95	22.64	1.75
ENSG00000169499	PLEKHA2	2.91	4.57	1.57
ENSG00000106086	PLEKHA8	2.79	4.43	1.59
ENSG00000090924	PLEKHG2	1.62	2.66	1.64
ENSG00000126822	PLEKHG3	5.08	7.66	1.51
ENSG00000196155	PLEKHG4	2.87	4.58	1.59
ENSG00000023902	PLEKHO1	0.59	2.07	3.54
ENSG00000183281	PLGLB1	0.95	1.85	1.95
ENSG00000145632	PLK2	2.51	6.03	2.41
ENSG00000142731	PLK4	1.92	6.84	3.57
ENSG00000083444	PLOD1	46.64	164.23	3.52
ENSG00000152952	PLOD2	0.08	9.73	128.36
ENSG00000102007	PLP2	10.00	15.21	1.52
ENSG00000187838	PLSCR3	1.53	2.31	1.51
ENSG00000164050	PLXNB1	2.28	4.10	1.80

ENSG00000185664	PMEL	0.72	1.42	1.98
ENSG00000132424	PNISR	5.83	10.35	1.78
ENSG00000183837	PNMA3	0.77	1.69	2.18
ENSG00000101868	POLA1	4.89	7.62	1.56
ENSG00000014138	POLA2	4.84	9.15	1.89
ENSG00000062822	POLD1	5.03	10.62	2.11
ENSG00000051341	POLQ	1.17	3.85	3.29
ENSG00000204531	POU5F1	0.86	2.09	2.43
ENSG00000143847	PPFIA4	0.16	6.33	40.26
ENSG00000104881	PPP1R13L	1.21	2.33	1.92
ENSG00000087074	PPP1R15A	5.85	10.40	1.78
ENSG00000146112	PPP1R18	0.80	1.40	1.74
ENSG00000173281	PPP1R3B	7.02	14.27	2.03
ENSG00000235194	PPP1R3E	4.68	7.84	1.68
ENSG00000167393	PPP2R3B	1.01	1.74	1.73
ENSG00000198901	PRC1	5.66	13.05	2.31
ENSG00000198056	PRIM1	1.10	2.04	1.85
ENSG00000188191	PRKAR1B	1.38	2.60	1.89
ENSG00000154229	PRKCA	2.33	3.92	1.68
ENSG00000115825	PRKD3	1.99	3.81	1.91
ENSG00000183943	PRKX	7.46	14.91	2.00
ENSG00000135406	PRPH	1.63	3.98	2.44
ENSG00000068489	PRR11	7.81	14.31	1.83
ENSG00000184838	PRR16	1.40	3.96	2.83
ENSG00000183248	FLJ22184	8.75	20.80	2.38
ENSG00000103023	PRSS54	0.00	1.45	385.37
ENSG00000204264	PSMB8	0.62	1.21	1.96
ENSG00000131470	PSMC3IP	1.19	2.67	2.25
ENSG00000134222	PSRC1	3.76	7.21	1.92
ENSG00000117569	PTBP2	1.15	1.72	1.50
ENSG00000171522	PTGER4	0.83	5.39	6.50
ENSG00000213413	PVRIG	1.30	2.80	2.15
ENSG00000130508	PXDN	2.46	5.85	2.38
ENSG00000167964	RAB26	19.15	33.27	1.74
ENSG00000137502	RAB30	2.19	3.79	1.73
ENSG00000168461	RAB31	1.35	4.42	3.28
ENSG00000105649	RAB3A	15.55	26.01	1.67
ENSG00000161800	RACGAP1	5.98	11.64	1.95
ENSG00000070950	RAD18	1.83	3.38	1.85

ENSG00000051180	RAD51	3.14	6.38	2.03
ENSG00000111247	RAD51AP1	6.82	13.97	2.05
ENSG00000197275	RAD54B	0.83	1.26	1.51
ENSG00000085999	RAD54L	4.08	9.95	2.44
ENSG00000160271	RALGDS	7.65	13.40	1.75
ENSG00000136237	RAPGEF5	1.20	1.95	1.63
ENSG00000108352	RAPGEFL1	2.52	3.79	1.50
ENSG00000077092	RARB	1.25	2.01	1.61
ENSG00000106538	RARRES2	0.61	1.00	1.65
ENSG00000155903	RASA2	2.26	3.43	1.52
ENSG00000101265	RASSF2	0.68	3.97	5.80
ENSG00000102054	RBBP7	8.47	12.90	1.52
ENSG00000080839	RBL1	2.14	3.66	1.71
ENSG00000147274	RBMX	28.63	45.07	1.57
ENSG00000139194	RBP5	1.49	2.44	1.64
ENSG00000168214	RBPJ	15.78	25.61	1.62
ENSG00000166831	RBPMS2	0.66	1.71	2.60
ENSG00000166965	RCCD1	6.31	10.24	1.62
ENSG00000142552	RCN3	5.35	12.09	2.26
ENSG00000054967	RELT	0.66	1.64	2.48
ENSG00000169891	REPS2	8.80	18.91	2.15
ENSG00000133119	RFC3	16.28	36.91	2.27
ENSG00000163918	RFC4	4.32	7.44	1.72
ENSG00000111445	RFC5	12.00	20.32	1.69
ENSG00000168411	RFWD3	4.20	7.67	1.83
ENSG00000080298	RFX3	1.37	2.50	1.83
ENSG00000242732	RGAG4	1.30	3.07	2.35
ENSG00000159496	RGL4	1.14	1.95	1.72
ENSG00000076344	RGS11	0.61	1.03	1.68
ENSG00000159788	RGS12	1.96	3.09	1.58
ENSG00000164292	RHOBTB3	0.75	2.42	3.24
ENSG00000166405	RIC3	1.27	1.92	1.51
ENSG00000080345	RIF1	3.00	4.80	1.60
ENSG00000188026	RILPL1	1.77	3.30	1.87
ENSG00000177181	RIMKLA	3.51	14.73	4.20
ENSG00000166532	RIMKLB	2.97	4.76	1.61
ENSG00000132669	RIN2	2.69	7.61	2.83
ENSG00000175643	RMI2	4.17	8.42	2.02
ENSG00000258818	RNASE4	6.82	12.92	1.89

ENSG00000104889	RNASEH2A	19.26	40.19	2.09
ENSG00000151692	RNF144A	5.40	9.61	1.78
ENSG00000141622	RNF165	0.57	1.09	1.90
ENSG00000101236	RNF24	8.75	13.18	1.51
ENSG00000204618	RNF39	0.55	1.10	2.00
ENSG00000108375	RNF43	2.91	7.78	2.67
ENSG00000069667	RORA	0.27	1.01	3.75
ENSG00000129824	RPS4Y1	160.31	243.86	1.52
ENSG00000083750	RRAGB	4.64	9.01	1.94
ENSG00000025039	RRAGD	3.64	5.60	1.54
ENSG00000167325	RRM1	27.36	54.81	2.00
ENSG00000171848	RRM2	13.11	56.94	4.34
ENSG00000081019	RSBN1	6.13	9.45	1.54
ENSG00000182010	RTKN2	0.91	2.22	2.45
ENSG00000197747	S100A10	0.32	1.94	6.02
ENSG00000213694	S1PR3	1.32	6.33	4.79
ENSG00000151835	SACS	1.85	6.76	3.65
ENSG00000164105	SAP30	3.34	7.08	2.12
ENSG00000228727	SAPCD1	0.58	1.53	2.66
ENSG00000156876	SASS6	1.68	3.60	2.15
ENSG00000073060	SCARB1	9.73	17.27	1.77
ENSG00000145284	SCD5	5.33	10.23	1.92
ENSG00000132330	SCLY	1.15	2.25	1.95
ENSG00000168447	SCNN1B	0.25	3.47	13.74
ENSG00000169439	SDC2	2.08	8.87	4.26
ENSG00000100003	SEC14L2	1.58	2.65	1.67
ENSG00000075223	SEMA3C	2.31	5.39	2.33
ENSG00000185033	SEMA4B	6.40	10.16	1.59
ENSG00000168758	SEMA4C	1.65	3.54	2.15
ENSG00000095539	SEMA4G	0.85	1.59	1.87
ENSG00000100167	41885	9.18	28.82	3.14
ENSG00000183569	SERHL2	2.14	4.03	1.88
ENSG00000168528	SERINC2	41.08	62.59	1.52
ENSG00000163536	SERPINI1	1.68	2.69	1.60
ENSG00000149212	SESN3	20.46	37.04	1.81
ENSG00000187231	SESTD1	1.16	2.06	1.77
ENSG00000152217	SETBP1	3.92	6.02	1.53
ENSG00000116560	SFPQ	18.11	33.74	1.86
ENSG00000107819	SFXN3	10.78	29.47	2.73

ENSG00000129810	SGOL1	1.34	3.00	2.24
ENSG00000163535	SGOL2	1.82	4.24	2.33
ENSG00000160999	SH2B2	2.01	3.86	1.92
ENSG00000100092	SH3BP1	7.39	11.15	1.51
ENSG00000214193	SH3D21	2.03	6.27	3.08
ENSG00000251322	SHANK3	3.55	6.13	1.73
ENSG00000171241	SHCBP1	3.93	6.52	1.66
ENSG00000116991	SIPA1L2	17.40	33.96	1.95
ENSG00000126778	SIX1	1.85	3.20	1.74
ENSG00000165480	SKA3	2.07	3.55	1.72
ENSG00000145604	SKP2	9.74	18.21	1.87
ENSG00000064651	SLC12A2	8.84	13.77	1.56
ENSG00000112394	SLC16A10	1.94	3.17	1.64
ENSG00000141526	SLC16A3	0.51	8.26	16.27
ENSG00000106688	SLC1A1	3.73	8.13	2.18
ENSG00000153291	SLC25A27	3.31	5.03	1.52
ENSG00000197119	SLC25A29	10.96	22.54	2.06
ENSG00000075303	SLC25A40	4.17	6.41	1.54
ENSG00000112759	SLC29A1	4.31	9.74	2.26
ENSG00000164638	SLC29A4	4.37	6.78	1.55
ENSG00000117394	SLC2A1	16.54	49.30	2.98
ENSG00000181856	SLC2A4	1.32	2.70	2.05
ENSG00000138821	SLC39A8	2.57	9.79	3.81
ENSG00000138449	SLC40A1	0.64	1.39	2.18
ENSG00000033867	SLC4A7	5.16	8.08	1.57
ENSG00000063127	SLC6A16	0.34	1.04	3.02
ENSG00000131389	SLC6A6	1.85	4.68	2.52
ENSG00000130821	SLC6A8	48.52	101.82	2.10
ENSG00000101187	SLCO4A1	11.42	17.45	1.53
ENSG00000137571	SLCO5A1	1.06	2.36	2.23
ENSG00000133302	ANKRD32	1.61	2.60	1.61
ENSG00000121871	SLITRK3	0.67	1.07	1.59
ENSG00000137834	SMAD6	3.19	5.98	1.87
ENSG00000136824	SMC2	5.43	13.89	2.56
ENSG00000108055	SMC3	19.19	29.51	1.54
ENSG00000113810	SMC4	7.49	17.30	2.31
ENSG00000183963	SMTN	4.88	7.44	1.52
ENSG00000143499	SMYD2	2.06	5.08	2.46
ENSG00000164975	SNAPC3	10.59	16.84	1.59

ENSG00000214265	SNURF	2.81	5.22	1.86
ENSG00000109762	SNX25	8.40	13.56	1.61
ENSG00000134243	SORT1	3.72	6.20	1.66
ENSG00000124766	SOX4	34.13	149.50	4.38
ENSG00000189120	SP6	1.91	4.06	2.13
ENSG00000076382	SPAG5	4.71	8.49	1.80
ENSG00000161888	SPC24	8.57	25.38	2.96
ENSG00000152253	SPC25	3.22	7.24	2.25
ENSG00000205238	SPDYE2	1.40	2.65	1.89
ENSG00000176170	SPHK1	1.47	3.11	2.12
ENSG00000269404	SPIB	0.75	1.31	1.75
ENSG00000163611	SPICE1	1.49	2.49	1.67
ENSG00000164056	SPRY1	4.01	8.58	2.14
ENSG00000184402	SS18L1	15.03	22.63	1.51
ENSG00000117155	SSX2IP	5.94	9.68	1.63
ENSG00000110080	ST3GAL4	6.29	14.04	2.23
ENSG00000185482	STAC3	0.77	1.53	1.99
ENSG00000164211	STARD4	5.36	9.21	1.72
ENSG00000173757	STAT5B	6.89	10.62	1.54
ENSG00000118804	STBD1	4.71	7.61	1.62
ENSG00000159167	STC1	0.15	13.69	89.67
ENSG00000123473	STIL	1.73	3.33	1.92
ENSG00000117632	STMN1	9.64	23.34	2.42
ENSG00000015592	STMN4	0.19	1.22	6.62
ENSG00000261052	SULT1A3	0.67	1.12	1.66
ENSG00000088002	SULT2B1	5.72	8.81	1.54
ENSG00000099994	SUSD2	1.05	1.94	1.85
ENSG00000101945	SUV39H1	3.63	5.96	1.64
ENSG00000152455	SUV39H2	4.16	6.61	1.59
ENSG00000133247	SUV420H2	4.34	6.64	1.53
ENSG00000161860	SYCE2	1.36	2.84	2.09
ENSG00000132872	SYT4	1.88	7.12	3.79
ENSG00000011347	SYT7	11.26	21.20	1.88
ENSG00000137501	SYTL2	0.67	1.48	2.20
ENSG00000013810	TACC3	18.68	31.32	1.68
ENSG00000149591	TAGLN	2.58	4.07	1.58
ENSG00000185418	TARSL2	1.81	2.93	1.62
ENSG00000177565	TBL1XR1	35.47	58.19	1.64
ENSG00000028839	TBPL1	8.94	13.74	1.54

ENSG00000170379	FAM115C	0.56	2.35	4.22
ENSG00000196507	TCEAL3	17.34	29.28	1.69
ENSG00000137310	TCF19	9.32	16.55	1.78
ENSG00000042088	TDP1	7.03	11.11	1.58
ENSG00000074219	TEAD2	1.60	5.09	3.17
ENSG00000009694	TENM1	3.85	8.12	2.11
ENSG00000164362	TERT	0.27	1.53	5.75
ENSG00000219435	C11orf20	1.03	2.44	2.37
ENSG00000160180	TFF3	1.57	4.64	2.96
ENSG00000003436	TFPI	10.37	16.12	1.55
ENSG00000069702	TGFBR3	2.05	3.66	1.79
ENSG00000118707	TGIF2	12.61	20.26	1.61
ENSG00000173451	THAP2	0.77	1.30	1.70
ENSG00000051596	THOC3	5.87	9.59	1.63
ENSG00000116001	TIA1	5.30	8.23	1.55
ENSG00000140534	TICRR	1.34	5.44	4.07
ENSG00000145365	TIFA	4.30	9.21	2.14
ENSG00000035862	TIMP2	15.54	24.78	1.59
ENSG00000167900	TK1	23.42	42.51	1.82
ENSG00000065717	TLE2	0.97	1.62	1.67
ENSG00000169908	TM4SF1	0.28	12.70	44.60
ENSG00000144339	TMEFF2	5.44	11.60	2.13
ENSG00000166292	TMEM100	0.12	2.54	21.03
ENSG00000179029	TMEM107	4.66	7.92	1.70
ENSG00000152558	TMEM123	75.50	130.05	1.72
ENSG00000006118	TMEM132A	27.52	46.58	1.69
ENSG00000138111	TMEM180	2.55	3.91	1.53
ENSG00000214128	TMEM213	0.73	1.29	1.78
ENSG00000151715	TMEM45B	7.94	14.95	1.88
ENSG00000105696	TMEM59L	1.73	4.66	2.70
ENSG00000125895	TMEM74B	0.29	1.97	6.84
ENSG00000109084	TMEM97	33.92	67.65	1.99
ENSG00000120802	TMPO	18.21	31.11	1.71
ENSG00000087128	TMPRSS11E	1.88	6.08	3.24
ENSG00000184012	TMPRSS2	52.81	101.93	1.93
ENSG00000205542	TMSB4X	18.60	37.98	2.04
ENSG00000185215	TNFAIP2	0.53	1.28	2.41
ENSG00000006327	TNFRSF12A	15.18	23.11	1.52
ENSG00000127863	TNFRSF19	14.31	38.47	2.69

ENSG00000215788	TNFRSF25	0.60	1.11	1.86
ENSG00000121858	TNFSF10	0.52	1.38	2.66
ENSG00000160949	TONSL	2.57	4.24	1.65
ENSG00000131747	TOP2A	4.42	13.31	3.01
ENSG00000177302	TOP3A	3.73	6.58	1.76
ENSG00000163781	TOPBP1	3.99	7.38	1.85
ENSG00000175274	TP53I11	14.88	25.49	1.71
ENSG00000078900	TP73	0.99	1.68	1.69
ENSG00000111669	TPI1	266.90	632.56	2.37
ENSG00000088325	TPX2	20.29	36.06	1.78
ENSG00000183763	TRAIP	1.19	1.95	1.65
ENSG00000173626	BET3L	1.03	1.81	1.75
ENSG00000132274	TRIM22	2.23	3.43	1.54
ENSG00000038382	TRIO	4.95	8.43	1.70
ENSG00000125733	TRIP10	6.43	10.25	1.60
ENSG00000071539	TRIP13	8.14	17.74	2.18
ENSG00000135451	TROAP	6.65	11.34	1.71
ENSG00000144481	TRPM8	1.21	10.04	8.28
ENSG00000162526	TSSK3	1.47	2.41	1.64
ENSG00000168026	TTC21A	0.75	1.25	1.66
ENSG00000182670	TTC3	11.43	17.52	1.53
ENSG00000112742	TTK	1.91	4.86	2.55
ENSG00000214021	TTLL3	2.75	4.67	1.70
ENSG00000137941	TTLL7	1.11	1.95	1.75
ENSG00000136295	TTYH3	13.55	21.48	1.59
ENSG00000167552	TUBA1A	1.69	7.91	4.68
ENSG00000123416	TUBA1B	81.72	138.89	1.70
ENSG00000075886	TUBA3D	2.37	4.99	2.11
ENSG00000196230	TUBB	257.21	488.59	1.90
ENSG00000258947	TUBB3	3.50	14.43	4.12
ENSG00000104833	TUBB4A	1.93	3.30	1.70
ENSG00000176014	TUBB6	2.11	4.83	2.29
ENSG00000122691	TWIST1	3.13	5.17	1.65
ENSG00000087301	TXNDC16	3.49	5.37	1.54
ENSG00000176890	TYMS	21.91	71.89	3.28
ENSG00000176912	C18orf56	7.57	14.05	1.86
ENSG00000175063	UBE2C	23.88	51.75	2.17
ENSG00000156587	UBE2L6	1.11	2.00	1.81
ENSG00000077152	UBE2T	7.13	16.84	2.36

ENSG00000109775	UFSP2	2.91	5.93	2.04
ENSG00000162738	VANGL2	1.53	3.05	1.99
ENSG00000143494	VASH2	1.40	4.40	3.14
ENSG00000112715	VEGFA	23.20	50.43	2.17
ENSG00000173511	VEGFB	7.52	44.42	5.91
ENSG00000026025	VIM	3.58	13.57	3.79
ENSG00000114812	VIPR1	5.34	8.04	1.51
ENSG00000167397	VKORC1	18.10	34.75	1.92
ENSG00000147852	VLDLR	4.09	7.55	1.84
ENSG00000197969	VPS13A	5.57	9.75	1.75
ENSG00000139722	VPS37B	20.36	31.94	1.57
ENSG00000100749	VRK1	6.35	10.34	1.63
ENSG00000132821	VSTM2L	1.77	6.01	3.39
ENSG00000185222	WBP5	39.35	60.08	1.53
ENSG00000198554	WDHD1	1.54	2.98	1.93
ENSG00000184465	WDR27	1.06	1.92	1.82
ENSG00000005448	WDR54	5.23	9.31	1.78
ENSG00000075702	WDR62	1.45	2.69	1.85
ENSG00000158023	WDR66	0.83	1.72	2.05
ENSG00000092470	WDR76	3.10	5.56	1.79
ENSG00000166483	WEE1	6.62	13.97	2.11
ENSG00000109685	WHSC1	9.32	15.35	1.65
ENSG00000116729	WLS	3.03	5.51	1.82
ENSG00000109046	WSB1	19.45	35.72	1.84
ENSG00000196584	XRCC2	3.56	7.78	2.18
ENSG00000163872	YEATS2	10.18	18.06	1.77
ENSG00000100027	YPEL1	1.42	5.30	3.74
ENSG00000179059	ZFP42	0.57	1.05	1.84
ENSG00000138658	C4orf21	0.36	1.16	3.21
ENSG00000108175	ZMIZ1	20.22	33.16	1.64
ENSG00000197020	ZNF100	3.35	5.28	1.58
ENSG00000196418	ZNF124	2.54	4.43	1.74
ENSG00000131127	ZNF141	3.28	6.28	1.92
ENSG00000170949	ZNF160	4.12	8.06	1.96
ENSG00000096654	ZNF184	2.93	4.57	1.56
ENSG00000005801	ZNF195	6.12	9.79	1.60
ENSG00000149050	ZNF214	1.15	1.84	1.60
ENSG00000159905	ZNF221	1.07	1.96	1.83
ENSG00000167840	ZNF232	7.00	11.36	1.62

ENSG00000196150	ZNF250	1.30	2.07	1.60
ENSG00000213096	ZNF254	6.46	9.74	1.51
ENSG00000198039	ZNF273	1.85	3.32	1.79
ENSG00000249459	ZNF286B	1.32	2.14	1.62
ENSG00000188994	ZNF292	1.07	3.79	3.53
ENSG00000130684	ZNF337	6.41	11.29	1.76
ENSG00000178338	ZNF354B	3.80	6.55	1.72
ENSG00000144331	ZNF385B	0.85	1.43	1.69
ENSG00000186918	ZNF395	4.60	19.03	4.14
ENSG00000198466	ZNF587	14.12	21.92	1.55
ENSG00000198521	ZNF43	4.13	6.62	1.60
ENSG00000118620	ZNF430	2.62	3.96	1.51
ENSG00000196705	ZNF431	4.29	7.03	1.64
ENSG00000249087	C1orf213	0.94	1.44	1.53
ENSG00000180855	ZNF443	2.09	3.18	1.52
ENSG00000178187	ZNF454	1.75	3.16	1.81
ENSG00000173258	ZNF483	1.33	2.23	1.68
ENSG00000175322	ZNF519	0.46	1.08	2.33
ENSG00000203326	ZNF525	3.98	7.07	1.78
ENSG00000183647	ZNF530	2.33	3.69	1.58
ENSG00000074657	ZNF532	10.17	16.00	1.57
ENSG00000213020	ZNF611	2.28	3.47	1.52
ENSG00000160229	ZNF66P	1.30	2.43	1.88
ENSG00000198046	ZNF667	3.83	6.33	1.65
ENSG00000188295	ZNF669	4.26	6.62	1.55
ENSG00000197372	ZNF675	2.41	3.85	1.59
ENSG00000164684	ZNF704	1.58	2.39	1.51
ENSG00000182141	ZNF708	5.05	8.37	1.66
ENSG00000196081	ZNF724P	1.48	3.17	2.14
ENSG00000183850	ZNF730	1.87	3.58	1.91
ENSG00000237440	ZNF737	5.81	9.28	1.60
ENSG00000172687	ZNF738	2.81	5.91	2.11
ENSG00000214189	ZNF788	0.72	1.22	1.70
ENSG00000167766	ZNF83	9.03	14.35	1.59
ENSG00000198040	ZNF84	8.78	16.55	1.89
ENSG00000178917	ZNF852	4.70	7.57	1.61
ENSG00000214029	ZNF891	2.74	4.36	1.59
ENSG00000146757	ZNF92	4.34	8.49	1.95
ENSG00000184635	ZNF93	0.92	2.15	2.34

ENSG00000149506	ZP1	0.29	1.18	4.13
ENSG00000176371	ZSCAN2	4.88	10.31	2.11
ENSG00000187987	ZSCAN23	1.84	3.84	2.09
ENSG00000132003	ZSWIM4	2.50	3.88	1.55
ENSG00000174442	ZWILCH	8.53	13.06	1.53
ENSG00000122952	ZWINT	9.59	25.75	2.69
ENSG00000203995	ZYG11A	0.71	1.07	1.51
ENSG00000196644	GPR89C	3.11	4.76	1.53
ENSG00000255054	RP1–317E23.6	1.85	3.29	1.78
ENSG00000255062	RP11–712L6.5	0.13	3.19	23.68
ENSG00000187951	ARHGAP11B	1.64	2.65	1.61
ENSG00000255439	VKORC1	5.70	11.00	1.93
ENSG00000017373	SRCIN1	1.56	2.71	1.73
ENSG00000187456	RDM1	1.11	2.04	1.84
ENSG00000197813	AC011450.1	1.25	3.86	3.08
ENSG00000223802	CERS1	4.32	9.26	2.14
ENSG00000266953	RP11–618P17.4	45.08	134.74	2.99
ENSG00000267740	AC024592.12	0.16	4.11	26.37
ENSG00000268193	AC002985.3	0.76	1.87	2.48
ENSG00000249590	RP4–539M6.19	8.63	15.46	1.79
ENSG00000206532	RP11–553A10.1	1.16	2.01	1.74
ENSG00000229729	RP11–159G9.5	3.36	5.73	1.70
ENSG00000214597	TMEM249	0.78	1.51	1.93
ENSG00000225885	AC023590.1	0.85	1.54	1.81
ENSG00000157950	SSX2B	1.09	1.90	1.75
ENSG00000205664	RP11–706O15.1	6.00	9.61	1.60

Supplementary Table S2: HypoxiaXshREST up M

		LNCaP-shREST			Fold change	
**Gene ID**	**Gene Symbol**	**0 day**	**3 day**	**6 day**	**3/0**	**6/0**
ENSG00000009724	MASP2	1.14	1.74	1.84	1.53	1.61
ENSG00000069812	HES2	1.24	1.67	1.86	1.35	1.50
ENSG00000077585	GPR137B	4.18	3.75	6.92	0.90	1.66
ENSG00000116729	WLS	5.88	9.11	9.06	1.55	1.54
ENSG00000116991	SIPA1L2	14.23	22.92	21.54	1.61	1.51
ENSG00000118292	C1orf54	0.41	0.89	1.41	2.17	3.44
ENSG00000134698	EIF2C4	4.43	7.28	7.23	1.64	1.63
ENSG00000135766	EGLN1	9.93	15.42	14.15	1.55	1.42
ENSG00000143387	CTSK	1.76	2.82	3.59	1.60	2.04
ENSG00000154358	OBSCN	1.41	2.66	3.45	1.89	2.45
ENSG00000159388	BTG2	14.95	29.85	25.03	2.00	1.67
ENSG00000162496	DHRS3	0.97	2.85	3.48	2.94	3.59
ENSG00000162526	TSSK3	1.84	2.55	3.16	1.39	1.72
ENSG00000162738	VANGL2	1.81	2.95	3.07	1.63	1.70
ENSG00000168243	GNG4	1.12	1.63	2.18	1.46	1.95
ENSG00000171385	KCND3	1.17	2.51	3.61	2.15	3.09
ENSG00000177181	RIMKLA	3.85	4.68	6.57	1.22	1.71
ENSG00000186973	FAM183A	0.48	0.79	1.00	1.65	2.08
ENSG00000188257	PLA2G2A	0.34	0.62	1.38	1.82	4.06
ENSG00000197520	FAM177B	0.83	1.19	1.51	1.43	1.82
ENSG00000214193	SH3D21	1.18	1.83	3.35	1.55	2.84
ENSG00000215788	TNFRSF25	0.87	1.19	2.04	1.37	2.34
ENSG00000240038	AMY2B	0.72	1.13	1.28	1.57	1.78
ENSG00000026025	VIM	0.68	0.80	1.21	1.18	1.78
ENSG00000108175	ZMIZ1	16.51	22.80	29.19	1.38	1.77
ENSG00000108242	CYP2C18	0.95	1.92	1.98	2.02	2.08
ENSG00000119946	CNNM1	1.23	1.65	2.53	1.34	2.06
ENSG00000119950	MXI1	8.07	16.29	16.25	2.02	2.01
ENSG00000122884	P4HA1	15.70	15.41	29.65	0.98	1.89
ENSG00000138109	CYP2C9	4.06	9.22	9.77	2.27	2.41
ENSG00000138111	TMEM180	2.57	3.50	4.75	1.36	1.85
ENSG00000138166	DUSP5	0.70	1.38	1.40	1.97	2.00
ENSG00000151640	DPYSL4	2.36	6.56	9.26	2.78	3.92
ENSG00000171988	JMJD1C	7.08	13.91	12.61	1.96	1.78
ENSG00000011347	SYT7	7.65	7.59	12.18	0.99	1.59
ENSG00000023445	BIRC3	0.81	1.37	1.84	1.69	2.27

ENSG00000080854	IGSF9B	1.06	1.56	2.04	1.47	1.92
ENSG00000110492	MDK	33.75	52.40	55.47	1.55	1.64
ENSG00000132274	TRIM22	1.71	3.61	3.77	2.11	2.20
ENSG00000137501	SYTL2	0.84	2.04	2.03	2.43	2.42
ENSG00000137502	RAB30	1.64	2.64	2.73	1.61	1.66
ENSG00000149050	ZNF214	1.15	1.80	1.98	1.57	1.72
ENSG00000149212	SESN3	20.35	39.35	44.26	1.93	2.17
ENSG00000149591	TAGLN	1.53	2.27	2.98	1.48	1.95
ENSG00000151715	TMEM45B	2.35	2.21	3.59	0.94	1.53
ENSG00000166405	RIC3	1.65	3.80	4.12	2.30	2.50
ENSG00000173511	VEGFB	5.87	8.40	12.51	1.43	2.13
ENSG00000175274	TP53I11	7.67	11.30	13.56	1.47	1.77
ENSG00000179240	RP11–111M22.2	1.45	2.02	2.51	1.39	1.73
ENSG00000187398	LUZP2	4.19	6.97	7.29	1.66	1.74
ENSG00000196167	C11orf92	7.12	11.13	16.36	1.56	2.30
ENSG00000111674	ENO2	3.52	5.17	10.18	1.47	2.89
ENSG00000123384	LRP1	1.06	2.36	2.83	2.23	2.67
ENSG00000135406	PRPH	1.18	1.40	2.45	1.19	2.08
ENSG00000135469	COQ10A	3.31	4.77	5.89	1.44	1.78
ENSG00000139194	RBP5	1.20	1.83	2.11	1.53	1.76
ENSG00000139625	MAP3K12	2.69	4.78	6.64	1.78	2.47
ENSG00000158023	WDR66	0.73	1.15	1.22	1.58	1.67
ENSG00000167767	KRT80	0.39	1.06	0.75	2.72	1.92
ENSG00000173451	THAP2	0.88	1.45	1.31	1.65	1.49
ENSG00000178498	DTX3	1.39	2.81	3.45	2.02	2.48
ENSG00000185482	STAC3	0.73	1.93	1.68	2.64	2.30
ENSG00000185633	NDUFA4L2	1.34	5.54	19.00	4.13	14.18
ENSG00000188026	RILPL1	1.71	3.30	3.75	1.93	2.19
ENSG00000198040	ZNF84	9.61	17.29	17.84	1.80	1.86
ENSG00000214029	ZNF891	2.97	4.82	5.25	1.62	1.77
ENSG00000240771	ARHGEF25	1.47	3.09	3.98	2.10	2.71
ENSG00000120658	ENOX1	6.81	11.15	13.31	1.64	1.95
ENSG00000127863	TNFRSF19	11.66	21.14	18.80	1.81	1.61
ENSG00000152767	FARP1	0.86	1.33	1.39	1.55	1.62
ENSG00000036530	CYP46A1	1.36	2.10	2.29	1.54	1.68
ENSG00000185215	TNFAIP2	0.38	0.92	1.26	2.42	3.32
ENSG00000196405	EVL	3.95	6.00	7.27	1.52	1.84
ENSG00000197119	SLC25A29	7.00	12.57	15.97	1.80	2.28
ENSG00000197406	DIO3	0.03	1.64	3.72	54.67	124.00

ENSG00000227051	C14orf132	1.34	1.71	2.10	1.28	1.57
ENSG00000258818	RNASE4	2.41	4.11	4.58	1.71	1.90
ENSG00000140398	NEIL1	3.08	6.07	8.25	1.97	2.68
ENSG00000156218	ADAMTSL3	6.68	10.09	9.59	1.51	1.44
ENSG00000166762	CATSPER2	1.68	3.14	3.28	1.87	1.95
ENSG00000166839	ANKDD1A	1.87	3.75	3.30	2.01	1.76
ENSG00000172379	ARNT2	1.77	2.68	2.92	1.51	1.65
ENSG00000175265	GOLGA8A	11.33	33.56	37.37	2.96	3.30
ENSG00000215252	GOLGA8B	5.05	16.46	16.68	3.26	3.30
ENSG00000006327	TNFRSF12A	4.31	9.01	8.84	2.09	2.05
ENSG00000102878	HSF4	3.80	5.35	8.05	1.41	2.12
ENSG00000167968	DNASE1L2	1.83	2.32	3.09	1.27	1.69
ENSG00000178773	CPNE7	11.93	15.04	19.92	1.26	1.67
ENSG00000183971	NPW	0.93	1.53	2.22	1.65	2.39
ENSG00000196123	KIAA0895L	10.04	12.22	15.96	1.22	1.59
ENSG00000196155	PLEKHG4	1.77	2.48	3.53	1.40	1.99
ENSG00000242173	ARHGDIG	3.42	4.95	7.05	1.45	2.06
ENSG00000257108	NHLRC4	0.92	1.08	1.86	1.17	2.02
ENSG00000017373	SRCIN1	0.69	1.48	1.91	2.14	2.77
ENSG00000035862	TIMP2	3.67	4.59	6.61	1.25	1.80
ENSG00000176749	CDK5R1	5.16	6.85	8.38	1.33	1.62
ENSG00000176845	METRNL	0.52	0.87	1.10	1.67	2.12
ENSG00000183979	NPB	0.08	1.17	0.03	14.63	0.38
ENSG00000186868	MAPT	0.75	1.27	1.74	1.69	2.32
ENSG00000198720	ANKRD13B	4.53	7.37	8.79	1.63	1.94
ENSG00000132872	SYT4	1.19	3.51	17.64	2.95	14.82
ENSG00000141665	FBXO15	2.31	3.62	3.93	1.57	1.70
ENSG00000152217	SETBP1	3.57	5.99	6.34	1.68	1.78
ENSG00000063180	CA11	2.68	4.60	5.47	1.72	2.04
ENSG00000087074	PPP1R15A	1.45	2.99	2.68	2.06	1.85
ENSG00000087076	HSD17B14	0.44	2.44	3.46	5.55	7.86
ENSG00000104808	DHDH	0.85	0.87	1.54	1.02	1.81
ENSG00000105088	OLFM2	0.32	1.17	2.03	3.66	6.34
ENSG00000105270	CLIP3	0.53	1.39	1.51	2.62	2.85
ENSG00000105290	APLP1	6.10	6.74	10.16	1.10	1.67
ENSG00000105696	TMEM59L	0.80	2.38	4.22	2.98	5.28
ENSG00000116014	KISS1R	0.19	0.27	1.17	1.42	6.16
ENSG00000130513	GDF15	44.87	311.01	365.37	6.93	8.14
ENSG00000130517	PGPEP1	5.11	11.47	12.48	2.24	2.44

ENSG00000130518	KIAA1683	0.48	1.26	1.54	2.63	3.21
ENSG00000130813	C19orf66	1.19	2.27	2.80	1.91	2.35
ENSG00000131398	KCNC3	1.29	2.91	3.61	2.26	2.80
ENSG00000132003	ZSWIM4	0.90	1.42	1.62	1.58	1.80
ENSG00000167766	ZNF83	8.55	12.75	14.88	1.49	1.74
ENSG00000170949	ZNF160	2.98	4.26	4.70	1.43	1.58
ENSG00000174788	PCP2	0.21	1.17	1.41	5.57	6.71
ENSG00000176490	DIRAS1	4.98	7.00	8.06	1.41	1.62
ENSG00000183248	FLJ22184	4.56	5.52	7.20	1.21	1.58
ENSG00000186567	CEACAM19	1.04	2.07	2.03	1.99	1.95
ENSG00000198046	ZNF667	3.23	4.49	5.07	1.39	1.57
ENSG00000223802	CERS1	1.59	1.98	2.64	1.25	1.66
ENSG00000267740	AC024592.12	0.00	7.41	0.07	7410000.00	70000.00
ENSG00000003436	TFPI	21.25	37.71	34.52	1.77	1.62
ENSG00000115325	DOK1	0.68	0.92	1.19	1.35	1.75
ENSG00000115548	KDM3A	1.96	3.61	4.14	1.84	2.11
ENSG00000125629	INSIG2	5.67	8.94	8.84	1.58	1.56
ENSG00000135976	ANKRD36	1.24	1.49	2.06	1.20	1.66
ENSG00000144199	FAHD2B	0.60	1.35	1.78	2.25	2.97
ENSG00000144339	TMEFF2	36.93	46.20	57.05	1.25	1.54
ENSG00000144485	HES6	23.53	59.16	59.18	2.51	2.52
ENSG00000152256	PDK1	8.32	10.33	12.52	1.24	1.50
ENSG00000163328	GPR155	1.92	3.21	3.10	1.67	1.61
ENSG00000163497	FEV	3.03	5.16	7.00	1.70	2.31
ENSG00000163516	ANKZF1	7.12	8.42	12.16	1.18	1.71
ENSG00000168280	KIF5C	3.39	6.58	6.70	1.94	1.98
ENSG00000169047	IRS1	1.34	3.05	2.96	2.28	2.21
ENSG00000183281	PLGLB1	1.07	2.21	1.98	2.07	1.85
ENSG00000118707	TGIF2	5.82	8.78	9.40	1.51	1.62
ENSG00000130684	ZNF337	5.41	12.20	13.23	2.26	2.45
ENSG00000132793	LPIN3	3.19	3.95	5.17	1.24	1.62
ENSG00000132821	VSTM2L	0.10	0.70	2.35	7.00	23.50
ENSG00000184402	SS18L1	15.21	23.96	24.29	1.58	1.60
ENSG00000215440	NPEPL1	1.32	1.99	2.44	1.51	1.85
ENSG00000160180	TFF3	0.67	2.43	4.33	3.63	6.46
ENSG00000184012	TMPRSS2	57.44	56.14	112.41	0.98	1.96
ENSG00000100027	YPEL1	1.30	2.03	2.76	1.56	2.12
ENSG00000100292	HMOX1	4.87	20.46	21.32	4.20	4.38
ENSG00000128342	LIF	1.38	3.84	3.72	2.78	2.70

ENSG00000133466	C1QTNF6	1.17	2.84	3.11	2.43	2.66
ENSG00000159496	RGL4	0.89	1.15	1.43	1.29	1.61
ENSG00000166897	ELFN2	0.26	0.80	1.00	3.08	3.85
ENSG00000185022	MAFF	0.61	0.86	1.14	1.41	1.87
ENSG00000188130	MAPK12	5.94	6.90	10.73	1.16	1.81
ENSG00000197182	FLJ27365	3.05	4.11	4.95	1.35	1.62
ENSG00000251322	SHANK3	2.06	2.79	3.60	1.35	1.75
ENSG00000114796	KLHL24	3.10	5.49	6.04	1.77	1.95
ENSG00000145020	AMT	0.69	1.93	2.90	2.80	4.20
ENSG00000152952	PLOD2	0.20	0.28	1.55	1.40	7.75
ENSG00000156976	EIF4A2	63.94	92.81	105.20	1.45	1.65
ENSG00000163536	SERPINI1	1.08	1.18	2.04	1.09	1.89
ENSG00000168026	TTC21A	0.51	1.06	1.08	2.08	2.12
ENSG00000170017	ALCAM	14.99	31.93	29.00	2.13	1.93
ENSG00000173531	MST1	1.83	4.98	8.23	2.72	4.50
ENSG00000176945	MUC20	0.96	1.72	1.51	1.79	1.57
ENSG00000177565	TBL1XR1	39.62	60.96	62.42	1.54	1.58
ENSG00000214021	TTLL3	2.40	4.38	4.69	1.83	1.95
ENSG00000228804	RP11–211G3.3	0.07	1.72	0.12	24.57	1.71
ENSG00000250151	ARPC4-TTLL3	3.59	6.74	9.65	1.88	2.69
ENSG00000072832	CRMP1	1.22	2.39	2.63	1.96	2.16
ENSG00000072840	EVC	4.09	5.84	7.05	1.43	1.72
ENSG00000109458	GAB1	1.17	2.26	1.99	1.93	1.70
ENSG00000109771	LRP2BP	0.80	1.09	1.22	1.36	1.53
ENSG00000138696	BMPR1B	3.96	4.76	7.32	1.20	1.85
ENSG00000145284	SCD5	5.05	9.42	11.74	1.87	2.32
ENSG00000168824	NSG1	0.54	1.00	0.97	1.85	1.80
ENSG00000205129	C4orf47	0.57	0.45	1.31	0.79	2.30
ENSG00000039139	DNAH5	1.21	2.49	2.77	2.06	2.29
ENSG00000113083	LOX	2.24	4.65	5.52	2.08	2.46
ENSG00000113108	APBB3	4.00	5.72	8.03	1.43	2.01
ENSG00000113240	CLK4	3.26	6.82	6.01	2.09	1.84
ENSG00000145632	PLK2	5.71	8.76	8.76	1.53	1.53
ENSG00000164406	LEAP2	2.00	3.20	2.38	1.60	1.19
ENSG00000249437	NAIP	1.78	2.57	3.27	1.44	1.84
ENSG00000025039	RRAGD	3.57	4.43	5.67	1.24	1.59
ENSG00000112715	VEGFA	5.93	22.96	25.38	3.87	4.28
ENSG00000131016	AKAP12	2.16	1.40	3.28	0.65	1.52
ENSG00000132424	PNISR	8.54	12.95	12.82	1.52	1.50

ENSG00000153291	SLC25A27	3.74	10.44	11.77	2.79	3.15
ENSG00000173626	BET3L	2.09	2.94	3.37	1.41	1.61
ENSG00000184465	WDR27	1.68	2.29	2.76	1.36	1.64
ENSG00000187987	ZSCAN23	2.38	3.14	3.87	1.32	1.63
ENSG00000204420	C6orf25	0.64	1.50	1.81	2.34	2.83
ENSG00000204618	RNF39	0.40	0.87	1.10	2.18	2.75
ENSG00000204681	GABBR1	1.35	4.53	4.90	3.36	3.63
ENSG00000213780	GTF2H4	0.79	3.09	0.84	3.91	1.06
ENSG00000055118	KCNH2	0.88	1.28	1.83	1.45	2.08
ENSG00000058404	CAMK2B	3.07	3.37	5.48	1.10	1.79
ENSG00000075618	FSCN1	1.39	1.98	2.09	1.42	1.50
ENSG00000105792	C7orf63	2.66	4.41	6.19	1.66	2.33
ENSG00000106004	HOXA5	1.74	2.35	3.22	1.35	1.85
ENSG00000106333	PCOLCE	0.45	0.76	1.16	1.69	2.58
ENSG00000136235	GPNMB	1.68	4.27	5.05	2.54	3.01
ENSG00000157224	CLDN12	20.48	35.89	40.57	1.75	1.98
ENSG00000164867	NOS3	0.80	2.99	4.99	3.74	6.24
ENSG00000188732	FAM221A	3.95	5.41	6.66	1.37	1.69
ENSG00000213420	GPC2	1.63	2.69	3.12	1.65	1.91
ENSG00000214128	TMEM213	0.93	1.43	1.48	1.54	1.59
ENSG00000091879	ANGPT2	0.86	3.20	4.67	3.72	5.43
ENSG00000104419	NDRG1	9.41	6.31	15.46	0.67	1.64
ENSG00000104765	BNIP3L	23.66	33.71	43.07	1.42	1.82
ENSG00000120875	DUSP4	8.90	16.00	18.55	1.80	2.08
ENSG00000167701	GPT	1.47	2.32	3.75	1.58	2.55
ENSG00000169499	PLEKHA2	1.02	1.83	2.19	1.79	2.15
ENSG00000185189	NRBP2	1.21	1.45	2.42	1.20	2.00
ENSG00000241852	C8orf58	0.57	1.04	0.86	1.82	1.51
ENSG00000248801	RP11–664D7.4	1.42	1.61	2.28	1.13	1.61
ENSG00000080298	RFX3	1.14	2.03	1.93	1.78	1.69
ENSG00000099139	PCSK5	2.63	3.11	4.46	1.18	1.70
ENSG00000106688	SLC1A1	2.12	2.92	4.02	1.38	1.90
ENSG00000106976	DNM1	0.58	1.35	1.64	2.33	2.83
ENSG00000107331	ABCA2	9.28	8.63	14.82	0.93	1.60
ENSG00000120217	CD274	1.70	3.95	7.26	2.32	4.27
ENSG00000136826	KLF4	2.39	3.97	4.03	1.66	1.69
ENSG00000136895	GARNL3	0.80	1.42	1.55	1.78	1.94
ENSG00000148180	GSN	0.54	0.99	1.20	1.83	2.22
ENSG00000160360	GPSM1	7.10	9.84	10.99	1.39	1.55

ENSG00000173258	ZNF483	1.72	2.47	2.95	1.44	1.72
ENSG00000230185	C9orf147	1.20	1.88	1.75	1.57	1.46
ENSG00000009694	TENM1	5.01	8.82	11.34	1.76	2.26
ENSG00000046651	OFD1	2.48	4.30	4.45	1.73	1.79
ENSG00000067840	PDZD4	0.74	0.94	1.58	1.27	2.14
ENSG00000102007	PLP2	1.72	2.34	3.26	1.36	1.90
ENSG00000129682	FGF13	0.82	3.15	2.73	3.84	3.33
ENSG00000165288	BRWD3	1.60	2.40	2.37	1.50	1.48
ENSG00000169891	REPS2	4.27	11.17	15.16	2.62	3.55
ENSG00000205542	TMSB4X	0.80	3.43	3.31	4.29	4.14
ENSG00000242732	RGAG4	1.61	2.90	2.83	1.80	1.76

Supplementary Table S3: Hypoxia down M

		LNCaP		
Gene ID	Gene Symbol	10% FBS	Hypoxia	Fold change
ENSG00000090861	AARS	113.63	56.69	0.50
ENSG00000197150	ABCB8	10.50	6.13	0.58
ENSG00000101986	ABCD1	45.00	27.34	0.61
ENSG00000114779	ABHD14B	54.72	26.37	0.48
ENSG00000100439	ABHD4	43.49	23.68	0.54
ENSG00000114626	ABTB1	1.78	1.16	0.65
ENSG00000115170	ACVR1	2.83	1.79	0.63
ENSG00000170634	ACYP2	4.41	2.57	0.58
ENSG00000063761	ADCK1	8.03	4.96	0.62
ENSG00000205336	GPR56	21.87	12.23	0.56
ENSG00000128165	ADM2	70.77	19.49	0.28
ENSG00000153531	ADPRHL1	18.71	10.72	0.57
ENSG00000130706	ADRM1	92.59	59.20	0.64
ENSG00000038002	AGA	10.04	6.23	0.62
ENSG00000106546	AHR	2.33	1.24	0.53
ENSG00000146416	AIG1	21.82	13.43	0.62
ENSG00000112297	AIM1	5.03	2.70	0.54
ENSG00000106305	AIMP2	75.78	41.92	0.55
ENSG00000129474	AJUBA	16.41	10.26	0.63
ENSG00000179841	AKAP5	4.13	2.10	0.51
ENSG00000106948	AKNA	2.72	1.74	0.64
ENSG00000072210	ALDH3A2	60.18	34.10	0.57
ENSG00000119523	ALG2	35.60	22.68	0.64
ENSG00000137760	ALKBH8	2.39	1.14	0.48
ENSG00000073331	ALPK1	3.09	1.96	0.64
ENSG00000167522	ANKRD11	23.53	14.50	0.62
ENSG00000089847	ANKRD24	2.39	1.21	0.51
ENSG00000154065	ANKRD29	1.74	0.92	0.53
ENSG00000137494	ANKRD42	4.29	2.79	0.65
ENSG00000138279	ANXA7	31.17	20.32	0.65
ENSG00000042753	AP2S1	63.31	38.56	0.61
ENSG00000163697	APBB2	3.37	1.72	0.51
ENSG00000184831	APOO	7.79	4.97	0.64
ENSG00000143761	ARF1	390.94	236.06	0.60
ENSG00000168374	ARF4	122.07	71.21	0.58
ENSG00000081181	ARG2	115.50	64.87	0.56

ENSG00000145819	ARHGAP26	4.63	2.69	0.58
ENSG00000116584	ARHGEF2	11.81	5.60	0.47
ENSG00000125962	ARMCX5	10.33	6.52	0.63
ENSG00000133794	ARNTL	3.46	2.19	0.63
ENSG00000130429	ARPC1B	31.16	11.65	0.37
ENSG00000136950	ARPC5L	63.73	35.52	0.56
ENSG00000140450	ARRDC4	14.20	3.27	0.23
ENSG00000204147	ASAH2B	8.15	5.34	0.66
ENSG00000088280	ASAP3	3.97	2.01	0.51
ENSG00000198356	ASNA1	103.43	66.67	0.64
ENSG00000070669	ASNS	52.30	15.02	0.29
ENSG00000148219	ASTN2	2.13	1.16	0.54
ENSG00000162772	ATF3	46.61	26.99	0.58
ENSG00000128272	ATF4	513.68	268.56	0.52
ENSG00000169136	ATF5	48.73	19.46	0.40
ENSG00000118217	ATF6	23.53	12.82	0.55
ENSG00000175224	ATG13	25.03	15.92	0.64
ENSG00000130734	ATG4D	22.88	14.41	0.63
ENSG00000070961	ATP2B1	16.87	8.44	0.50
ENSG00000064270	ATP2C2	16.81	7.77	0.46
ENSG00000124788	ATXN1	5.92	1.68	0.28
ENSG00000086062	B4GALT1	91.60	47.64	0.52
ENSG00000127152	BCL11B	1.13	0.14	0.13
ENSG00000105829	BET1	8.24	5.10	0.62
ENSG00000177951	BET1L	41.25	22.98	0.56
ENSG00000133134	BEX2	106.82	41.44	0.39
ENSG00000090013	BLVRB	79.39	41.86	0.53
ENSG00000113734	BNIP1	23.45	13.52	0.58
ENSG00000165714	LOH12CR1	8.89	5.52	0.62
ENSG00000106245	BUD31	64.50	36.08	0.56
ENSG00000112276	BVES	2.00	1.22	0.61
ENSG00000168070	C11orf85	1.16	0.75	0.64
ENSG00000189227	C15orf61	17.35	9.63	0.56
ENSG00000166246	C16orf71	1.71	0.87	0.51
ENSG00000174109	C16orf91	39.86	26.06	0.65
ENSG00000205710	C17orf107	5.84	2.15	0.37
ENSG00000224877	C17orf89	105.80	68.80	0.65
ENSG00000187624	C17orf97	3.00	1.96	0.65
ENSG00000197982	C1orf122	62.32	34.84	0.56

ENSG00000203724	C1orf53	17.43	7.51	0.43
ENSG00000171984	C20orf196	5.80	3.71	0.64
ENSG00000159079	C21orf59	10.76	7.10	0.66
ENSG00000128346	C22orf23	6.26	2.92	0.47
ENSG00000237651	C2orf74	3.12	1.91	0.61
ENSG00000151881	C5orf28	15.97	6.41	0.40
ENSG00000164241	C5orf63	4.40	2.89	0.66
ENSG00000204387	C6orf48	236.13	150.77	0.64
ENSG00000137434	C6orf52	1.66	0.74	0.45
ENSG00000104205	SGK3	6.01	3.48	0.58
ENSG00000238227	C9orf69	45.95	29.76	0.65
ENSG00000165698	C9orf9	10.05	6.31	0.63
ENSG00000136436	CALCOCO2	25.44	16.36	0.64
ENSG00000112186	CAP2	22.79	9.79	0.43
ENSG00000149260	CAPN5	22.61	5.52	0.24
ENSG00000187796	CARD9	4.92	3.19	0.65
ENSG00000110619	CARS	6.95	4.01	0.58
ENSG00000147044	CASK	5.35	3.19	0.60
ENSG00000196954	CASP4	3.72	2.05	0.55
ENSG00000121691	CAT	79.85	46.45	0.58
ENSG00000139899	CBLN3	3.19	2.03	0.63
ENSG00000141582	CBX4	124.51	66.78	0.54
ENSG00000048342	CC2D2A	4.45	2.77	0.62
ENSG00000103021	CCDC113	16.77	9.54	0.57
ENSG00000166946	CCNDBP1	15.68	10.20	0.65
ENSG00000163249	CCNYL1	6.01	3.85	0.64
ENSG00000196352	CD55	8.76	1.82	0.21
ENSG00000176386	CDC26	1.90	1.17	0.61
ENSG00000128283	CDC42EP1	12.03	7.89	0.66
ENSG00000147883	CDKN2B	1.58	1.02	0.65
ENSG00000153879	CEBPG	59.63	28.36	0.48
ENSG00000093072	CECR1	1.19	0.64	0.54
ENSG00000160401	C9orf117	5.09	2.90	0.57
ENSG00000128965	CHAC1	77.56	7.07	0.09
ENSG00000250479	CHCHD10	77.47	48.89	0.63
ENSG00000163528	CHCHD4	30.98	18.48	0.60
ENSG00000123989	CHPF	49.12	25.56	0.52
ENSG00000109572	CLCN3	55.35	34.85	0.63
ENSG00000073464	CLCN4	5.44	1.97	0.36

ENSG00000106404	CLDN15	6.87	3.81	0.55
ENSG00000181885	CLDN7	196.09	124.98	0.64
ENSG00000153132	CLGN	27.28	10.21	0.37
ENSG00000169504	CLIC4	66.96	32.65	0.49
ENSG00000175416	CLTB	22.99	14.03	0.61
ENSG00000125246	CLYBL	1.27	0.64	0.50
ENSG00000091317	CMTM6	44.41	28.95	0.65
ENSG00000205423	CNEP1R1	4.17	2.63	0.63
ENSG00000100528	CNIH	74.15	44.03	0.59
ENSG00000136152	COG3	28.39	18.07	0.64
ENSG00000084636	COL16A1	1.06	0.57	0.53
ENSG00000163359	COL6A3	23.38	11.14	0.48
ENSG00000173163	COMMD1	11.61	6.56	0.57
ENSG00000110442	COMMD9	11.92	7.28	0.61
ENSG00000165644	COMTD1	13.17	7.44	0.56
ENSG00000129083	COPB1	20.38	9.91	0.49
ENSG00000105669	COPE	28.08	16.06	0.57
ENSG00000181789	COPG1	139.56	73.81	0.53
ENSG00000203667	COX20	15.76	9.69	0.61
ENSG00000115944	COX7A2L	28.72	17.58	0.61
ENSG00000137449	CPEB2	2.49	1.08	0.43
ENSG00000107864	CPEB3	2.61	1.53	0.58
ENSG00000110090	CPT1A	39.04	24.20	0.62
ENSG00000157184	CPT2	45.29	29.85	0.66
ENSG00000107175	CREB3	50.53	28.15	0.56
ENSG00000182158	CREB3L2	15.45	10.10	0.65
ENSG00000095794	CREM	4.89	2.44	0.50
ENSG00000150938	CRIM1	4.58	2.55	0.56
ENSG00000121552	CSTA	2.67	0.63	0.24
ENSG00000126890	CTAG2	1.43	0.75	0.53
ENSG00000150527	CTAGE5	12.17	4.53	0.37
ENSG00000117151	CTBS	17.39	11.25	0.65
ENSG00000175215	CTDSP2	49.93	31.20	0.62
ENSG00000116761	CTH	34.66	8.30	0.24
ENSG00000064601	CTSA	9.58	6.28	0.66
ENSG00000117984	CTSD	104.73	69.10	0.66
ENSG00000135047	CTSL1	59.57	34.67	0.58
ENSG00000140465	CYP1A1	1.86	0.92	0.49
ENSG00000120306	CYSTM1	37.48	20.01	0.53

ENSG00000189186	DCAF8L2	1.30	0.85	0.66
ENSG00000204843	DCTN1	8.94	5.44	0.61
ENSG00000132437	DDC	10.63	3.63	0.34
ENSG00000175197	DDIT3	114.66	39.34	0.34
ENSG00000168209	DDIT4	472.74	278.31	0.59
ENSG00000105671	DDX49	43.90	25.58	0.58
ENSG00000155792	DEPTOR	10.44	4.13	0.40
ENSG00000116133	DHCR24	211.42	132.49	0.63
ENSG00000100867	DHRS2	1.90	0.77	0.40
ENSG00000187630	DHRS4L2	9.64	6.14	0.64
ENSG00000086189	DIMT1	8.83	5.56	0.63
ENSG00000268471	DKFZP434I0714	14.69	8.76	0.60
ENSG00000103423	DNAJA3	43.14	25.29	0.59
ENSG00000128590	DNAJB9	31.75	17.26	0.54
ENSG00000136770	DNAJC1	10.98	7.17	0.65
ENSG00000077232	DNAJC10	13.44	7.94	0.59
ENSG00000120675	DNAJC15	4.70	2.80	0.60
ENSG00000105612	DNASE2	44.71	25.40	0.57
ENSG00000137976	DNASE2B	8.92	5.83	0.65
ENSG00000213221	DNLZ	3.64	2.15	0.59
ENSG00000129932	DOHH	14.31	9.06	0.63
ENSG00000173852	DPY19L1	1.40	0.71	0.51
ENSG00000125821	DTD1	61.84	39.23	0.63
ENSG00000198842	DUSP27	11.25	1.18	0.10
ENSG00000145088	EAF2	1.50	0.55	0.37
ENSG00000167969	ECI1	27.09	17.73	0.65
ENSG00000088298	EDEM2	9.47	5.35	0.56
ENSG00000114654	CCDC48	7.18	3.59	0.50
ENSG00000138798	EGF	7.96	4.45	0.56
ENSG00000113790	EHHADH	2.31	1.45	0.63
ENSG00000119718	EIF2B2	29.08	17.65	0.61
ENSG00000163412	EIF4E3	1.60	1.04	0.65
ENSG00000187840	EIF4EBP1	328.85	154.09	0.47
ENSG00000075151	EIF4G3	8.05	4.89	0.61
ENSG00000141642	ELAC1	4.79	2.11	0.44
ENSG00000111145	ELK3	3.32	1.89	0.57
ENSG00000138080	EMILIN1	1.27	0.60	0.47
ENSG00000165675	ENOX2	4.53	2.95	0.65
ENSG00000168032	ENTPD3	6.13	2.08	0.34

ENSG00000187097	ENTPD5	13.78	8.89	0.65
ENSG00000115109	EPB41L5	14.56	8.35	0.57
ENSG00000086289	EPDR1	2.26	0.98	0.43
ENSG00000142627	EPHA2	1.85	1.15	0.62
ENSG00000044524	EPHA3	5.48	3.24	0.59
ENSG00000135333	EPHA7	2.07	1.35	0.65
ENSG00000143819	EPHX1	217.72	94.02	0.43
ENSG00000172031	EPHX4	2.12	1.34	0.63
ENSG00000136628	EPRS	31.43	17.54	0.56
ENSG00000068912	ERLEC1	30.46	18.27	0.60
ENSG00000164010	ERMAP	8.81	5.78	0.66
ENSG00000178607	ERN1	23.37	9.04	0.39
ENSG00000089248	ERP29	131.70	74.48	0.57
ENSG00000104413	ESRP1	67.23	28.39	0.42
ENSG00000139641	ESYT1	68.26	44.56	0.65
ENSG00000105672	ETV2	1.76	0.58	0.33
ENSG00000142459	EVI5L	8.55	5.24	0.61
ENSG00000138190	EXOC6	5.21	3.34	0.64
ENSG00000120699	EXOSC8	22.33	14.33	0.64
ENSG00000182197	EXT1	1.83	1.01	0.55
ENSG00000169122	FAM110B	4.13	2.34	0.57
ENSG00000135842	FAM129A	22.19	6.93	0.31
ENSG00000196227	FAM217B	8.80	5.01	0.57
ENSG00000203778	C6orf225	5.69	3.30	0.58
ENSG00000204805	FAM27E4P	11.47	7.12	0.62
ENSG00000168672	FAM84B	49.41	25.69	0.52
ENSG00000107872	FBXL15	13.88	8.23	0.59
ENSG00000197361	FBXL22	4.60	1.91	0.42
ENSG00000037637	FBXO42	11.47	6.87	0.60
ENSG00000162746	FCRLB	1.29	0.74	0.58
ENSG00000161513	FDXR	6.77	4.44	0.66
ENSG00000105550	FGF21	2.18	0.48	0.22
ENSG00000198855	FICD	9.55	4.94	0.52
ENSG00000100442	FKBP3	5.51	3.36	0.61
ENSG00000119686	FLVCR2	4.41	2.43	0.55
ENSG00000139445	FOXN4	1.18	0.63	0.54
ENSG00000150907	FOXO1	2.10	1.16	0.55
ENSG00000070601	FRMPD1	6.79	3.27	0.48
ENSG00000150667	FSIP1	1.65	0.88	0.53

ENSG00000226124	AC073043.2	1.10	0.68	0.62
ENSG00000140564	FURIN	34.27	14.30	0.42
ENSG00000174951	FUT1	8.17	4.33	0.53
ENSG00000171124	FUT3	2.83	1.52	0.54
ENSG00000165060	FXN	12.40	8.13	0.66
ENSG00000156958	GALK2	10.95	7.15	0.65
ENSG00000141441	FAM59A	2.34	1.41	0.60
ENSG00000106105	GARS	202.98	61.74	0.30
ENSG00000059691	PET112	1.97	1.30	0.66
ENSG00000107862	GBF1	43.02	28.25	0.66
ENSG00000104381	GDAP1	6.97	3.60	0.52
ENSG00000198380	GFPT1	39.27	17.23	0.44
ENSG00000168237	GLYCTK	2.56	1.02	0.40
ENSG00000144591	GMPPA	12.46	6.49	0.52
ENSG00000127955	GNAI1	73.23	37.83	0.52
ENSG00000156052	GNAQ	2.12	1.39	0.66
ENSG00000066455	GOLGA5	35.55	23.10	0.65
ENSG00000135052	GOLM1	81.82	51.78	0.63
ENSG00000120053	GOT1	45.24	21.63	0.48
ENSG00000152642	GPD1L	13.17	8.31	0.63
ENSG00000183671	GPR1	2.63	0.31	0.12
ENSG00000112218	GPR63	3.70	2.03	0.55
ENSG00000164294	GPX8	8.67	3.50	0.40
ENSG00000106070	GRB10	32.11	16.67	0.52
ENSG00000030582	GRN	39.38	23.68	0.60
ENSG00000164284	GRPEL2	41.42	25.57	0.62
ENSG00000100983	GSS	35.04	22.34	0.64
ENSG00000065621	GSTO2	1.78	1.08	0.61
ENSG00000172432	GTPBP2	28.16	13.21	0.47
ENSG00000246705	H2AFJ	92.28	55.94	0.61
ENSG00000131373	HACL1	2.20	1.43	0.65
ENSG00000143575	HAX1	136.75	72.87	0.53
ENSG00000163517	HDAC11	10.26	6.55	0.64
ENSG00000184508	HDDC3	19.18	11.65	0.61
ENSG00000138642	HERC6	4.44	2.32	0.52
ENSG00000051108	HERPUD1	104.84	40.81	0.39
ENSG00000113924	HGD	1.52	1.00	0.66
ENSG00000235173	FAM203A	10.19	6.27	0.62
ENSG00000137133	HINT2	38.30	19.91	0.52

ENSG00000180573	HIST1H2AC	106.58	54.79	0.51
ENSG00000180596	HIST1H2BC	41.58	13.29	0.32
ENSG00000124635	HIST1H2BJ	18.16	5.22	0.29
ENSG00000233822	HIST1H2BN	10.91	5.60	0.51
ENSG00000197409	HIST1H3D	22.56	12.78	0.57
ENSG00000197153	HIST1H3J	3.23	1.31	0.41
ENSG00000203814	HIST2H2BF	14.18	8.73	0.62
ENSG00000159267	HLCS	14.06	7.51	0.53
ENSG00000101294	HM13	63.12	41.18	0.65
ENSG00000221887	HMSD	1.21	0.35	0.29
ENSG00000108511	HOXB6	4.21	2.52	0.60
ENSG00000170689	HOXB9	9.83	4.08	0.41
ENSG00000198353	HOXC4	2.29	1.39	0.61
ENSG00000002587	HS3ST1	1.43	0.54	0.38
ENSG00000204228	HSD17B8	59.31	32.94	0.56
ENSG00000155304	HSPA13	26.98	14.05	0.52
ENSG00000149428	HYOU1	47.18	25.26	0.54
ENSG00000196305	IARS	24.47	14.11	0.58
ENSG00000138413	IDH1	103.90	35.52	0.34
ENSG00000068079	IFI35	10.47	3.42	0.33
ENSG00000115267	IFIH1	2.23	1.47	0.66
ENSG00000185745	IFIT1	2.33	0.90	0.38
ENSG00000006652	IFRD1	21.54	12.43	0.58
ENSG00000119650	IFT43	11.66	6.33	0.54
ENSG00000197081	IGF2R	66.79	37.87	0.57
ENSG00000183067	IGSF5	1.15	0.34	0.29
ENSG00000134470	IL15RA	1.06	0.57	0.54
ENSG00000172458	IL17D	4.01	2.52	0.63
ENSG00000174564	IL20RB	1.06	0.48	0.46
ENSG00000132376	INPP5K	6.20	3.34	0.54
ENSG00000136003	ISCU	32.18	14.69	0.46
ENSG00000159556	ISL2	2.95	1.21	0.41
ENSG00000091409	ITGA6	20.61	13.07	0.63
ENSG00000086544	ITPKC	12.22	7.62	0.62
ENSG00000150995	ITPR1	6.61	2.50	0.38
ENSG00000077684	PHF17	7.01	4.07	0.58
ENSG00000188385	JAKMIP3	3.50	2.22	0.63
ENSG00000153814	JAZF1	1.26	0.65	0.51
ENSG00000140044	JDP2	9.07	4.00	0.44

ENSG00000161677	JOSD2	13.38	8.15	0.61
ENSG00000143543	JTB	114.58	70.40	0.61
ENSG00000026559	KCNG1	1.72	1.13	0.65
ENSG00000235750	KIAA0040	3.96	1.75	0.44
ENSG00000164659	KIAA1324L	3.50	2.30	0.66
ENSG00000165757	KIAA1462	1.01	0.64	0.63
ENSG00000102554	KLF5	2.53	1.09	0.43
ENSG00000162755	KLHDC9	20.01	12.97	0.65
ENSG00000171798	KNDC1	1.79	0.82	0.46
ENSG00000147592	LACTB2	18.31	9.37	0.51
ENSG00000053747	LAMA3	29.71	16.95	0.57
ENSG00000185896	LAMP1	60.47	39.69	0.66
ENSG00000078081	LAMP3	1.38	0.64	0.47
ENSG00000166173	LARP6	27.27	12.06	0.44
ENSG00000205629	LCMT1	20.14	12.81	0.64
ENSG00000148346	LCN2	16.05	1.13	0.07
ENSG00000136167	LCP1	54.25	28.36	0.52
ENSG00000166816	LDHD	14.40	7.05	0.49
ENSG00000205213	LGR4	4.42	2.86	0.65
ENSG00000183250	C21orf67	3.55	1.87	0.53
ENSG00000107798	LIPA	12.37	6.39	0.52
ENSG00000168216	LMBRD1	5.55	3.47	0.63
ENSG00000143013	LMO4	18.02	8.42	0.47
ENSG00000136944	LMX1B	2.55	1.65	0.65
ENSG00000206535	LNP1	5.57	3.46	0.62
ENSG00000232671	RP11–126K1.2	3.11	1.79	0.58
ENSG00000196365	LONP1	43.11	25.45	0.59
ENSG00000167419	LPO	1.10	0.52	0.47
ENSG00000197324	LRP10	50.04	28.45	0.57
ENSG00000079691	LRRC16A	4.84	2.69	0.56
ENSG00000010626	LRRC23	7.26	4.66	0.64
ENSG00000141294	LRRC46	1.83	0.92	0.50
ENSG00000108829	LRRC59	100.49	53.07	0.53
ENSG00000171492	LRRC8D	46.39	17.38	0.37
ENSG00000181817	LSM10	40.60	25.36	0.62
ENSG00000160886	LY6K	1.01	0.48	0.48
ENSG00000205707	LYRM5	21.99	11.64	0.53
ENSG00000163818	LZTFL1	2.63	1.66	0.63
ENSG00000099866	MADCAM1	1.11	0.68	0.61

ENSG00000102316	MAGED2	103.55	49.11	0.47
ENSG00000187391	MAGI2	1.23	0.77	0.63
ENSG00000112893	MAN2A1	3.85	2.53	0.66
ENSG00000140941	MAP1LC3B	34.41	13.43	0.39
ENSG00000047849	MAP4	29.70	19.54	0.66
ENSG00000116871	MAP7D1	42.46	24.29	0.57
ENSG00000069956	MAPK6	93.70	59.88	0.64
ENSG00000173926	41701	1.16	0.67	0.57
ENSG00000116141	MARK1	4.78	2.39	0.50
ENSG00000166986	MARS	66.96	28.66	0.43
ENSG00000180398	MCFD2	34.96	21.95	0.63
ENSG00000140563	MCTP2	2.16	1.32	0.61
ENSG00000065833	ME1	25.31	12.21	0.48
ENSG00000156603	MED19	15.56	9.66	0.62
ENSG00000160563	MED27	9.99	6.55	0.66
ENSG00000159479	MED8	16.70	10.87	0.65
ENSG00000143995	MEIS1	1.02	0.47	0.46
ENSG00000172878	METAP1D	5.41	3.54	0.65
ENSG00000214756	METTL12	14.23	9.26	0.65
ENSG00000101574	METTL4	3.36	2.18	0.65
ENSG00000198948	MFAP3L	3.68	1.93	0.52
ENSG00000118855	MFSD1	24.73	15.92	0.64
ENSG00000167700	MFSD3	31.85	19.20	0.60
ENSG00000008394	MGST1	75.30	48.79	0.65
ENSG00000100139	MICALL1	26.30	12.07	0.46
ENSG00000165175	MID1IP1	140.27	61.22	0.44
ENSG00000080561	MID2	9.15	5.55	0.61
ENSG00000125457	MIF4GD	14.57	7.58	0.52
ENSG00000167965	MLST8	58.72	32.80	0.56
ENSG00000139428	MMAB	11.78	7.47	0.63
ENSG00000130675	MNX1	11.64	4.95	0.43
ENSG00000075643	MOCOS	38.51	11.12	0.29
ENSG00000164077	MON1A	4.94	3.01	0.61
ENSG00000171160	MORN4	18.81	11.10	0.59
ENSG00000073146	MOV10L1	1.18	0.47	0.40
ENSG00000060762	MPC1	9.63	6.11	0.63
ENSG00000254858	MPV17L2	16.72	9.56	0.57
ENSG00000153029	MR1	3.98	2.22	0.56
ENSG00000175110	MRPS22	18.47	11.72	0.63

ENSG00000147586	MRPS28	22.73	14.30	0.63
ENSG00000061794	MRPS35	143.07	84.45	0.59
ENSG00000175806	MSRA	3.69	1.60	0.43
ENSG00000148450	MSRB2	32.32	21.09	0.65
ENSG00000174099	MSRB3	1.43	0.75	0.52
ENSG00000163132	MSX1	11.03	6.32	0.57
ENSG00000120149	MSX2	2.89	1.77	0.61
ENSG00000125144	MT1G	41.88	25.57	0.61
ENSG00000168502	SOGA2	2.72	1.63	0.60
ENSG00000066855	MTFR1	28.36	17.55	0.62
ENSG00000120254	MTHFD1L	13.73	8.59	0.63
ENSG00000065911	MTHFD2	111.69	45.01	0.40
ENSG00000104643	MTMR9	10.53	6.86	0.65
ENSG00000172732	MUS81	11.60	6.84	0.59
ENSG00000074842	C19orf10	57.90	36.89	0.64
ENSG00000152620	NADKD1	28.79	18.85	0.65
ENSG00000108784	NAGLU	37.01	22.14	0.60
ENSG00000186462	NAP1L2	10.76	3.59	0.33
ENSG00000134440	NARS	71.63	42.57	0.59
ENSG00000020129	NCDN	22.57	14.75	0.65
ENSG00000124151	NCOA3	7.92	4.79	0.60
ENSG00000111912	NCOA7	10.00	5.24	0.52
ENSG00000137806	NDUFAF1	15.50	9.82	0.63
ENSG00000164182	NDUFAF2	56.71	32.04	0.56
ENSG00000109390	NDUFC1	3.97	2.38	0.60
ENSG00000114670	NEK11	1.20	0.68	0.57
ENSG00000160602	NEK8	5.41	3.20	0.59
ENSG00000082641	NFE2L1	148.50	90.31	0.61
ENSG00000169599	NFU1	2.06	0.87	0.42
ENSG00000101004	NINL	1.67	0.52	0.31
ENSG00000104361	NIPAL2	8.08	5.00	0.62
ENSG00000136783	NIPSNAP3A	57.60	34.37	0.60
ENSG00000112981	NME5	3.90	2.22	0.57
ENSG00000141458	NPC1	8.52	4.21	0.49
ENSG00000181019	NQO1	23.85	10.00	0.42
ENSG00000025434	NR1H3	6.24	4.01	0.64
ENSG00000106459	NRF1	19.61	8.14	0.42
ENSG00000073969	NSF	36.57	23.58	0.64
ENSG00000070081	NUCB2	22.86	14.81	0.65

ENSG00000168101	NUDT16L1	35.94	23.13	0.64
ENSG00000176046	NUPR1	284.14	167.66	0.59
ENSG00000135114	OASL	1.78	0.47	0.26
ENSG00000065154	OAT	3.35	1.86	0.56
ENSG00000087263	OGFOD1	22.13	13.18	0.60
ENSG00000162600	OMA1	9.01	5.82	0.65
ENSG00000079156	OSBPL6	1.70	0.59	0.35
ENSG00000116885	OSCP1	4.43	2.79	0.63
ENSG00000140961	OSGIN1	2.98	1.52	0.51
ENSG00000089723	OTUB2	5.32	2.94	0.55
ENSG00000101104	PABPC1L	21.86	10.97	0.50
ENSG00000100266	PACSIN2	37.74	23.95	0.63
ENSG00000217930	PAM16	3.49	2.23	0.64
ENSG00000073150	PANX2	5.52	2.24	0.41
ENSG00000167081	PBX3	2.92	1.37	0.47
ENSG00000156453	PCDH1	15.45	4.02	0.26
ENSG00000100889	PCK2	206.33	40.43	0.20
ENSG00000156973	PDE6D	3.64	2.11	0.58
ENSG00000104213	PDGFRL	2.18	0.59	0.27
ENSG00000164494	PDSS2	9.32	6.09	0.65
ENSG00000198300	PEG3	20.67	12.04	0.58
ENSG00000124299	PEPD	4.16	2.61	0.63
ENSG00000157911	PEX10	44.42	23.60	0.53
ENSG00000162928	PEX13	16.71	10.62	0.64
ENSG00000092621	PHGDH	202.48	101.92	0.50
ENSG00000008710	PKD1	10.85	7.14	0.66
ENSG00000135549	PKIB	14.75	9.04	0.61
ENSG00000165495	PKNOX2	4.02	1.27	0.32
ENSG00000168907	PLA2G4F	5.85	3.49	0.60
ENSG00000170965	PLAC1	1.48	0.22	0.15
ENSG00000187091	PLCD1	1.94	1.16	0.60
ENSG00000115762	PLEKHB2	46.45	30.59	0.66
ENSG00000152527	PLEKHH2	1.18	0.55	0.47
ENSG00000120594	PLXDC2	5.74	3.24	0.56
ENSG00000141682	PMAIP1	19.25	11.89	0.62
ENSG00000013503	POLR3B	14.31	8.39	0.59
ENSG00000188242	CTD-2228K2.5	26.71	16.56	0.62
ENSG00000112033	PPARD	11.05	7.22	0.65
ENSG00000168938	PPIC	9.17	4.13	0.45

ENSG00000164088	PPM1M	6.57	3.09	0.47
ENSG00000066027	PPP2R5A	20.08	11.91	0.59
ENSG00000126432	PRDX5	457.53	273.14	0.60
ENSG00000138078	PREPL	10.30	5.32	0.52
ENSG00000005249	PRKAR2B	13.14	6.78	0.52
ENSG00000184500	PROS1	2.30	1.29	0.56
ENSG00000148426	C10orf47	9.40	5.88	0.63
ENSG00000205352	PRR13	38.17	24.86	0.65
ENSG00000204983	PRSS1	6.94	1.66	0.24
ENSG00000112812	PRSS16	9.71	6.17	0.64
ENSG00000010438	PRSS3	1.08	0.44	0.41
ENSG00000135069	PSAT1	439.75	113.42	0.26
ENSG00000161057	PSMC2	50.71	31.59	0.62
ENSG00000146733	PSPH	38.86	14.23	0.37
ENSG00000165983	PTER	15.82	9.66	0.61
ENSG00000184007	PTP4A2	37.15	23.78	0.64
ENSG00000158079	PTPDC1	15.84	6.00	0.38
ENSG00000163629	PTPN13	9.12	5.31	0.58
ENSG00000070778	PTPN21	2.76	1.43	0.52
ENSG00000187024	PTRH1	2.08	1.32	0.64
ENSG00000150787	PTS	25.74	16.01	0.62
ENSG00000185129	PURA	8.41	5.20	0.62
ENSG00000168297	PXK	6.35	3.85	0.61
ENSG00000183010	PYCR1	117.57	74.20	0.63
ENSG00000170473	WIBG	30.03	19.32	0.64
ENSG00000116260	QSOX1	46.07	26.32	0.57
ENSG00000169228	RAB24	9.83	6.30	0.64
ENSG00000172007	RAB33B	4.33	2.18	0.50
ENSG00000172794	RAB37	1.39	0.81	0.58
ENSG00000167994	RAB3IL1	3.69	2.24	0.61
ENSG00000172780	RAB43	24.54	14.77	0.60
ENSG00000171570	RAB4B	28.70	16.25	0.57
ENSG00000105404	RABAC1	10.97	5.66	0.52
ENSG00000154710	RABGEF1	14.08	8.61	0.61
ENSG00000155918	RAET1L	2.80	1.53	0.55
ENSG00000184672	RALYL	1.17	0.50	0.42
ENSG00000123728	RAP2C	7.40	4.86	0.66
ENSG00000173166	RAPH1	5.67	2.67	0.47
ENSG00000125826	RBCK1	139.76	62.58	0.45

ENSG00000171174	RBKS	1.31	0.70	0.53
ENSG00000112183	RBM24	24.40	11.54	0.47
ENSG00000155636	RBM45	4.25	2.24	0.53
ENSG00000121039	RDH10	5.08	2.62	0.52
ENSG00000072042	RDH11	120.08	71.19	0.59
ENSG00000104856	RELB	1.28	0.72	0.56
ENSG00000189056	RELN	2.62	0.98	0.37
ENSG00000139890	REM2	1.77	1.14	0.65
ENSG00000042445	RETSAT	28.93	14.45	0.50
ENSG00000076043	REXO2	42.62	26.72	0.63
ENSG00000175449	RFESD	1.06	0.40	0.38
ENSG00000144468	RHBDD1	11.07	5.52	0.50
ENSG00000100263	RHBDD3	14.98	9.88	0.66
ENSG00000079841	RIMS1	3.59	2.25	0.63
ENSG00000249884	RNF103-CHMP3	17.82	11.18	0.63
ENSG00000137393	RNF144B	7.48	3.30	0.44
ENSG00000168159	RNF187	383.82	188.93	0.49
ENSG00000163481	RNF25	5.37	3.55	0.66
ENSG00000105982	RNF32	2.09	1.29	0.62
ENSG00000181852	RNF41	58.46	29.65	0.51
ENSG00000189050	RNFT1	3.35	2.09	0.62
ENSG00000067836	ROGDI	13.93	9.01	0.65
ENSG00000185483	ROR1	3.16	1.37	0.43
ENSG00000124787	RPP40	5.89	3.21	0.55
ENSG00000156990	RPUSD3	7.42	4.60	0.62
ENSG00000137996	RTCA	35.92	22.84	0.64
ENSG00000115310	RTN4	49.19	31.49	0.64
ENSG00000156253	RWDD2B	7.59	4.93	0.65
ENSG00000189334	S100A14	1.37	0.79	0.57
ENSG00000163993	S100P	200.60	13.20	0.07
ENSG00000031698	SARS	194.54	84.79	0.44
ENSG00000092108	SCFD1	16.22	9.90	0.61
ENSG00000121064	SCPEP1	18.89	12.19	0.65
ENSG00000128228	SDF2L1	75.59	48.38	0.64
ENSG00000139410	SDSL	32.49	9.43	0.29
ENSG00000166562	SEC11C	46.66	29.15	0.62
ENSG00000150961	SEC24D	6.16	2.97	0.48
ENSG00000106803	SEC61B	292.65	193.02	0.66
ENSG00000091490	SEL1L3	20.89	8.44	0.40

ENSG00000143416	SELENBP1	49.57	29.04	0.59
ENSG00000135622	SEMA4F	3.82	2.43	0.64
ENSG00000166192	SENP8	4.92	3.00	0.61
ENSG00000179918	SEPHS2	160.92	64.95	0.40
ENSG00000120742	SERP1	94.03	61.86	0.66
ENSG00000196136	SERPINA3	2.17	0.33	0.15
ENSG00000166401	SERPINB8	2.15	1.19	0.55
ENSG00000166224	SGPL1	40.03	22.52	0.56
ENSG00000197860	SGTB	6.85	3.92	0.57
ENSG00000185437	SH3BGR	10.52	2.89	0.27
ENSG00000147010	SH3KBP1	2.90	1.57	0.54
ENSG00000125089	SH3TC1	1.76	1.12	0.64
ENSG00000196470	SIAH1	13.93	6.80	0.49
ENSG00000165449	SLC16A9	5.70	3.24	0.57
ENSG00000115902	SLC1A4	13.02	4.42	0.34
ENSG00000105281	SLC1A5	105.95	32.03	0.30
ENSG00000146477	SLC22A3	56.35	31.00	0.55
ENSG00000178537	SLC25A20	10.98	4.50	0.41
ENSG00000136868	SLC31A1	92.22	34.73	0.38
ENSG00000164414	SLC35A1	3.45	1.75	0.51
ENSG00000102100	SLC35A2	23.70	13.84	0.58
ENSG00000213699	C2orf18	30.61	18.54	0.61
ENSG00000176273	SLC35G1	3.44	1.93	0.56
ENSG00000157637	SLC38A10	23.15	14.57	0.63
ENSG00000141873	SLC39A3	22.84	15.06	0.66
ENSG00000112473	SLC39A7	134.36	79.45	0.59
ENSG00000168003	SLC3A2	107.37	47.84	0.45
ENSG00000167703	SLC43A2	8.17	3.84	0.47
ENSG00000158715	SLC45A3	122.83	74.23	0.60
ENSG00000211584	SLC48A1	11.78	6.79	0.58
ENSG00000088836	SLC4A11	1.07	0.43	0.41
ENSG00000196517	SLC6A9	16.86	3.37	0.20
ENSG00000139514	SLC7A1	88.53	50.30	0.57
ENSG00000151012	SLC7A11	65.83	18.28	0.28
ENSG00000103257	SLC7A5	66.63	19.30	0.29
ENSG00000090020	SLC9A1	17.28	10.22	0.59
ENSG00000197818	SLC9A8	9.87	5.32	0.54
ENSG00000170365	SMAD1	6.65	4.21	0.63
ENSG00000224531	C6orf228	19.93	12.55	0.63

ENSG00000163683	C4orf34	40.59	21.19	0.52
ENSG00000250317	C4orf52	50.20	31.55	0.63
ENSG00000088826	SMOX	8.37	2.80	0.33
ENSG00000166311	SMPD1	33.33	19.08	0.57
ENSG00000135587	SMPD2	15.00	9.69	0.65
ENSG00000019549	SNAI2	1.52	0.67	0.44
ENSG00000174446	SNAPC5	19.84	12.66	0.64
ENSG00000128739	SNRPN	16.75	11.02	0.66
ENSG00000002919	SNX11	12.33	7.77	0.63
ENSG00000064652	SNX24	3.60	1.70	0.47
ENSG00000112320	SOBP	1.97	1.00	0.51
ENSG00000120669	SOHLH2	12.09	7.91	0.65
ENSG00000137642	SORL1	5.97	3.01	0.50
ENSG00000104450	SPAG1	4.99	2.35	0.47
ENSG00000134278	SPIRE1	15.31	8.67	0.57
ENSG00000167778	SPRYD3	21.04	12.51	0.59
ENSG00000161011	SQSTM1	85.07	55.84	0.66
ENSG00000174780	SRP72	83.62	54.59	0.65
ENSG00000182934	SRPR	49.16	30.69	0.62
ENSG00000144867	SRPRB	56.42	32.95	0.58
ENSG00000114850	SSR3	87.37	49.35	0.56
ENSG00000138134	STAMBPL1	1.65	0.96	0.58
ENSG00000072786	STK10	1.53	0.90	0.59
ENSG00000144589	STK11IP	3.46	2.14	0.62
ENSG00000081320	STK17B	9.65	5.16	0.54
ENSG00000169302	STK32A	1.28	0.54	0.42
ENSG00000196182	STK40	23.01	11.86	0.52
ENSG00000162236	STX5	17.26	10.75	0.62
ENSG00000166263	STXBP4	3.16	1.96	0.62
ENSG00000164506	STXBP5	4.45	2.73	0.61
ENSG00000129103	SUMF2	74.67	42.81	0.57
ENSG00000148291	SURF2	36.39	20.16	0.55
ENSG00000148248	SURF4	157.88	103.78	0.66
ENSG00000197321	SVIL	3.52	1.56	0.44
ENSG00000175854	SWI5	28.42	17.74	0.62
ENSG00000138162	TACC2	15.76	7.06	0.45
ENSG00000136463	TACO1	43.62	28.66	0.66
ENSG00000197780	TAF13	38.21	24.35	0.64
ENSG00000187325	TAF9B	24.57	13.97	0.57

ENSG00000204267	TAP2	8.20	4.47	0.55
ENSG00000113407	TARS	83.60	51.66	0.62
ENSG00000095383	TBC1D2	1.32	0.76	0.57
ENSG00000136111	TBC1D4	12.51	7.22	0.58
ENSG00000154144	TBRG1	37.18	18.35	0.49
ENSG00000187735	TCEA1	62.29	34.99	0.56
ENSG00000196116	TDRD7	4.00	2.59	0.65
ENSG00000166848	TERF2IP	60.08	34.74	0.58
ENSG00000135269	TES	47.68	30.13	0.63
ENSG00000088992	TESC	1.06	0.68	0.65
ENSG00000068323	TFE3	24.87	14.29	0.57
ENSG00000092295	TGM1	2.39	0.57	0.24
ENSG00000113272	THG1L	7.48	4.58	0.61
ENSG00000078237	C12orf5	13.74	8.45	0.62
ENSG00000104980	TIMM44	13.16	6.85	0.52
ENSG00000143155	TIPRL	53.60	31.95	0.60
ENSG00000149809	TM7SF2	74.36	46.17	0.62
ENSG00000150403	TMCO3	95.54	46.17	0.48
ENSG00000170006	TMEM154	2.43	1.30	0.54
ENSG00000168701	TMEM208	49.10	30.68	0.62
ENSG00000169964	TMEM42	22.74	14.36	0.63
ENSG00000103978	TMEM87A	16.40	10.64	0.65
ENSG00000167920	TMEM99	24.64	15.05	0.61
ENSG00000067182	TNFRSF1A	21.22	11.87	0.56
ENSG00000117586	TNFSF4	2.24	1.18	0.53
ENSG00000111907	TPD52L1	17.25	6.84	0.40
ENSG00000141933	TPGS1	10.60	6.79	0.64
ENSG00000166340	TPP1	28.66	18.39	0.64
ENSG00000102871	TRADD	11.79	6.95	0.59
ENSG00000167515	TRAPPC2L	11.78	7.24	0.61
ENSG00000182400	TRAPPC6B	10.44	6.62	0.63
ENSG00000101255	TRIB3	234.09	59.59	0.25
ENSG00000154370	TRIM11	15.00	9.45	0.63
ENSG00000221926	TRIM16	12.47	7.92	0.64
ENSG00000136932	C9orf156	5.12	3.32	0.65
ENSG00000163467	TSACC	3.64	1.96	0.54
ENSG00000157514	TSC22D3	55.22	19.69	0.36
ENSG00000198860	TSEN15	40.15	21.93	0.55
ENSG00000106537	TSPAN13	131.66	81.77	0.62

ENSG00000048140	TSPAN17	18.35	11.47	0.63
ENSG00000258992	TSPY1	2.21	1.44	0.65
ENSG00000032389	TSSC1	3.86	2.52	0.65
ENSG00000113312	TTC1	49.78	29.97	0.60
ENSG00000018699	TTC27	11.38	6.71	0.59
ENSG00000172425	TTC36	4.80	3.11	0.65
ENSG00000155158	TTC39B	4.30	1.66	0.39
ENSG00000100271	TTLL1	4.83	2.88	0.60
ENSG00000074935	TUBE1	4.01	2.60	0.65
ENSG00000247596	TWF2	22.86	14.37	0.63
ENSG00000115514	TXNDC9	6.65	4.11	0.62
ENSG00000025708	TYMP	4.80	3.02	0.63
ENSG00000161265	U2AF1L4	4.32	2.21	0.51
ENSG00000118939	UCHL3	43.53	18.28	0.42
ENSG00000109814	UGDH	19.98	11.17	0.56
ENSG00000135226	UGT2B28	5.60	3.53	0.63
ENSG00000065060	UHRF1BP1	21.89	12.86	0.59
ENSG00000107731	UNC5B	1.60	1.04	0.65
ENSG00000183696	UPP1	4.88	1.41	0.29
ENSG00000126088	UROD	27.96	17.96	0.64
ENSG00000168899	VAMP5	1.25	0.68	0.55
ENSG00000128218	VPREB3	2.97	1.79	0.60
ENSG00000139719	VPS33A	5.37	3.50	0.65
ENSG00000140105	WARS	40.34	13.22	0.33
ENSG00000139668	WDFY2	3.90	2.50	0.64
ENSG00000065268	WDR18	34.62	22.05	0.64
ENSG00000176473	WDR25	3.69	2.34	0.63
ENSG00000196998	WDR45	6.98	3.71	0.53
ENSG00000135925	WNT10A	3.64	2.12	0.58
ENSG00000151718	WWC2	1.01	0.55	0.55
ENSG00000186153	WWOX	2.92	1.85	0.63
ENSG00000198373	WWP2	18.04	10.32	0.57
ENSG00000100219	XBP1	128.64	59.28	0.46
ENSG00000184575	XPOT	141.60	80.52	0.57
ENSG00000134684	YARS	28.94	12.03	0.42
ENSG00000166913	YWHAB	154.54	98.85	0.64
ENSG00000104219	ZDHHC2	8.57	5.53	0.64
ENSG00000174165	ZDHHC24	19.74	12.28	0.62
ENSG00000104231	ZFAND1	26.27	17.16	0.65

ENSG00000156639	ZFAND3	59.65	33.65	0.56
ENSG00000220201	ZGLP1	1.57	0.98	0.63
ENSG00000066185	ZMYND12	2.58	1.67	0.65
ENSG00000147394	ZNF185	1.91	0.85	0.45
ENSG00000085644	ZNF213	9.81	5.80	0.59
ENSG00000159915	ZNF233	1.94	1.04	0.54
ENSG00000138311	ZNF365	1.05	0.44	0.42
ENSG00000105136	ZNF419	18.57	8.51	0.46
ENSG00000268107	AC003005.4	5.30	3.05	0.58
ENSG00000198342	ZNF442	1.44	0.95	0.66
ENSG00000180035	ZNF48	4.18	2.75	0.66
ENSG00000083814	ZNF671	3.91	2.30	0.59
ENSG00000152439	ZNF773	4.78	2.98	0.62
ENSG00000223547	ZNF844	10.12	6.28	0.62
ENSG00000174276	ZNHIT2	34.73	21.21	0.61
ENSG00000170044	ZPLD1	1.66	0.18	0.11
ENSG00000117289	TXNIP	158.42	5.85	0.04
ENSG00000136653	RASSF5	4.96	3.04	0.61
ENSG00000182217	HIST2H4B	35.73	8.76	0.25
ENSG00000183941	HIST2H4A	79.94	25.95	0.32
ENSG00000258466	RP11–1012A1.4	48.10	26.02	0.54
ENSG00000258941	MIA2	16.74	5.94	0.35
ENSG00000205456	TP53TG3	1.04	0.59	0.57
ENSG00000161326	DUSP14	38.11	22.24	0.58
ENSG00000267618	RAD51L3-RFFL	7.97	4.67	0.59
ENSG00000167807	FDX1L	7.94	4.90	0.62
ENSG00000258674	YJEFN3	1.76	0.95	0.54
ENSG00000268434	AC011530.4	1.38	0.79	0.57
ENSG00000234949	AC104667.3	10.13	4.82	0.48
ENSG00000247121	CTD-2260A17.2	3.03	1.96	0.65
ENSG00000234432	AC092171.1	12.03	5.67	0.47
ENSG00000255046	RP11–297N6.4	1.78	1.08	0.61
ENSG00000185554	NXF2	16.29	8.12	0.50

Supplementary Table S4: HypoxiaXshREST down M

		LNCaP-shREST			Fold change	
**Gene ID**	**Gene Symbol**	**0 day**	**3 day**	**6 day**	**3/0**	**6/0**
ENSG00000157911	PEX10	69.68	37.98	41.78	0.55	0.60
ENSG00000165644	COMTD1	14.60	8.94	9.80	0.61	0.67
ENSG00000176273	SLC35G1	3.78	2.58	2.29	0.68	0.61
ENSG00000246705	H2AFJ	4.70	2.68	3.36	0.57	0.71
ENSG00000102554	KLF5	2.43	2.22	1.51	0.91	0.62
ENSG00000100867	DHRS2	4.17	0.45	0.49	0.11	0.12
ENSG00000168907	PLA2G4F	11.49	4.48	6.15	0.39	0.54
ENSG00000103257	SLC7A5	33.10	21.45	23.44	0.65	0.71
ENSG00000140961	OSGIN1	1.73	0.96	1.24	0.55	0.72
ENSG00000181019	NQO1	24.03	11.81	12.56	0.49	0.52
ENSG00000108829	LRRC59	53.39	32.58	31.89	0.61	0.60
ENSG00000042753	AP2S1	72.32	38.13	40.63	0.53	0.56
ENSG00000254858	MPV17L2	13.28	8.75	9.08	0.66	0.68
ENSG00000064601	CTSA	9.70	5.62	7.54	0.58	0.78
ENSG00000128228	SDF2L1	51.78	19.30	24.63	0.37	0.48
ENSG00000145088	EAF2	4.55	1.12	1.12	0.25	0.25
ENSG00000206535	LNP1	1.87	1.20	1.48	0.64	0.79
ENSG00000164182	NDUFAF2	40.38	31.55	26.13	0.78	0.65
ENSG00000124635	HIST1H2BJ	27.64	4.93	8.72	0.18	0.32
ENSG00000086289	EPDR1	2.26	1.49	1.98	0.66	0.88
ENSG00000127955	GNAI1	2.64	3.00	1.44	1.14	0.55
ENSG00000147586	MRPS28	32.72	21.48	21.88	0.66	0.67
ENSG00000235173	FAM203A	8.50	5.52	6.74	0.65	0.79
ENSG00000101986	ABCD1	16.04	9.21	11.54	0.57	0.72
ENSG00000184831	APOO	36.71	23.68	24.88	0.65	0.68
